# Health service access and delivery for people living with rare disorders: a scoping review

**DOI:** 10.1186/s13023-026-04455-7

**Published:** 2026-06-27

**Authors:** Tara N. Officer, Michael Roguski, Lucy Bennett, Bruce C. Officer, Lisa Ye, Gretchen Good, Karen McBride-Henry

**Affiliations:** 1 Te Puna Hauora | School of Health, Te Pukenga Wai | Faculty of Education, Health, and Psychological Sciences, Te Herenga Waka | Victoria University of Wellington, Wellington, New Zealand; 2 Andcer Consulting Ltd., Wellington, New Zealand; 3 Kaitiaki Research and Evaluation, Wellington, New Zealand; 4 Research Centre on Public Health, University of Milano-Bicocca, Monza, Italy; 5 School of Health Sciences, Massey University, Palmerston North, New Zealand

**Keywords:** Rare disorders, Rare diseases, Scoping review, Health service delivery, Healthcare experiences, Healthcare access, Qualitative methods

## Abstract

**Background:**

Rare disorders contribute significant collective health system costs; individuals living with rare disorders frequently encounter diagnostic, treatment, and management barriers. Despite international recognition of these challenges, there remains limited research addressing systemic health service delivery and access barriers for those with rare disorders, or identification of how such research informs policymaking.

**Methods:**

Dimensions, PubMed, Scopus, ProQuest, CINAHL, and Ovid platform databases were employed in a search of qualitative research documenting the health service experiences of people living with rare disorders and their primary support networks. A total of 4,615 records were identified and after de-duplication, screened by title, keywords, and abstract. Seventy-eight publications met the inclusion criteria after full text screening, these were then reviewed. Policy impact in the form of citations was determined by whether an Overton search identified a link between the reviewed studies and policy documents.

**Results:**

Two primary findings were identified. (1) Health service access and delivery barriers are pervasive. People living with rare disorders and their support networks manage emotional, financial, and social challenges. There is an urgent need for improved service delivery, including better access, education, psychological, and peer support. (2) Reviewed studies, covering only a small subset of identified rare disorders, are published largely in specialised rare disorders journals and often failed to adopt inclusive methods. Few included publications were cited in policy-related documents retrieved through Overton, and several identified citations were incidental rather than substantive.

**Interpretation:**

Despite growing awareness, health systems fall short of addressing structural barriers faced by people living with rare disorders. The findings support development of a global coordinated action plan for rare disorder management and its subsequent implementation following adoption. For researchers, this could include adoption of inclusive research methods. For policymakers, there is a need for stronger inclusion of equity-priority populations and coordinated policy frameworks that recognise the collective impact of disparate and poorly managed care. Critical to supporting change is investment in the upskilling of the health workforce and clinicians’ active engagement in diagnosis, coordination, long-term management, and treatment.

## Introduction

Rare disorders (RDs) are low-prevalence health conditions [1], often defined as affecting fewer than one in 2,000 people. There are over 10,000 different RDs; collectively they affect over 300 million people worldwide [2]. Notably, 50% of RDs affect children and, amongst these children, there is a 30% mortality rate before five years old [2]. Diagnosing RDs is challenging; individuals often experience delayed diagnoses, leading to underreporting [1]. Rare disorders are commonly associated with concomitant impairment and/ or disability and the need for additional health services [3, 4]. Stressors associated with RDs also extend across family and social support networks, constraining social, employment, and broader vocational participation for individuals and their families, influencing economic security and long-term wellbeing [5]. Unsurprisingly, treatment and management research has identified significant direct and indirect health system costs [6, 7]. For example, the total 2019 economic burden in the United States was placed at close to USD1 trillion [8] for 379 RDs, and in 2016, RDs accounted for nearly half of that nation’s estimated national inpatient hospital charges, with adult and paediatric RD populations having longer hospital stays, more charges per each admission, substantial readmissions, and greater in hospital mortality [7].

The COVID-19 pandemic exacerbated challenges for disabled people and people living with RDs (PLWRDs) [4], with significant disruptions to health services reported [9–13]. While emerging international research emphasises additional burdens faced by those with RDs during the pandemic [14, 15], it can take years, and numerous consultations with various healthcare professionals before an accurate RD diagnosis is made. Rather than providing an understanding of collective access-related experiences of PLWRDs, empirical research has been largely limited by small sample sizes, a focus on specific conditions, or has been undertaken within particular clinics [16]. What is recognised is that PLWRDs have varying success in accessing healthcare professionals with condition-specific knowledge [3, 17–19], and face gaps in health services, disability support, and social support integration [16, 20, 21]. Given the rarity of disorders, PLWRDs tend to become condition-specific experts. However, self-agency is often thwarted by dismissive attitudes and discrimination, leaving individuals feeling invalidated and societally isolated [18, 22].

United Nations General Assembly Resolution A/RES/76/132, Addressing the challenges of persons living with a rare disease and their families, [4] recognises challenges associated with RDs and calls for countries to develop strategies to alleviate inequities the RDs community faces. More recently, World Health Assembly Resolution WHA78.11, Rare diseases: a global health priority for equity and inclusion, recognised the complex, multifaceted needs of PLWRDs and their families and requested the development of a comprehensive ten-year global action plan on rare diseases [5]. The resolution highlighted discrimination, psychosocial and economic vulnerability, inequitable access to timely diagnosis and treatment, disability amongst some PLWRDs, and the importance of universal health coverage, integrated care, and social inclusion. Concerningly, recent reviews [23, 24] emphasise geographic differences in healthcare policy impact and implementation, but also barriers to universal health coverage in the RD community [25]. Against a backdrop of uneven implementation and sustainability of national rare disorder strategies/ plans [26], it is important to understand how RD research is being conducted and used internationally.

This scoping review examines the nature of published literature on RD care, with a focus on experiences of health service access and delivery. In addition, the review considers whose voices are included in the evidence base, recommendations made to support equitable outcomes, and how this research informs policymaking.

## Methods

Our review was guided by the scoping review methodology established by Arksey and O’Malley, and reported in accordance with the PRISMA extension designed for scoping reviews [27, 28]. The search protocol was made available before the review commenced [29] and was reviewed by a health subject librarian. When beginning the review, the planned approach was adapted from an Application Programming Interface-based search to a search across six platforms or interfaces to improve feasibility and completeness. These changes were pragmatic and did not alter review aims. We applied predefined criteria (Table 1) to our search of Dimensions, PubMed, Scopus, ProQuest, CINAHL, and Ovid (Embase, Emcare, Global Health, JBI EBP Database, MEDLINE) databases. The search was conducted during the week of 20 May 2024 and included studies published from 1 January 2020. Grey literature was explicitly excluded, and the review was limited to peer-reviewed journal articles. This decision reflected our focus on understanding research-generated evidence, where methods, recruitment strategies, and analytical approaches should be explicitly described, enabling more consistent interpretation across studies. Given one of our aims was examining how the reviewed studies informed policy, our approach also allowed clearer delineation between evidence production and translational outputs.

**Table 1:** Inclusion and exclusion criteria

**Inclusion**
• Full text peer-reviewed journal articles published in English covering healthcare access and service delivery from the perspectives of PLWRDs and/ or their primary support networks (family, parents/ guardians, partners, informal carers) • Published between 1 January 2020 and 20 May 2024 • Included sufficient methodological detail to undergo a JBI quality review process • Involved qualitative designs or self-reported accounts
**Exclusion**
• Non-English language publications • Published before 1 January 2020 • Non-human studies • Clinical trials or intervention studies • Protocols • Studies addressing RD genetic research or epidemiology • Non-qualitative outcomes • Surveys with limited consideration of open-ended question responses • Conference proceedings • Expert opinion • Editorials • Grey literature • Books or book chapters

**Table 2:** Search term variations and filters

Rare disorders	(orphan OR rare) AND (disorder* OR disease* OR illness* OR condition* OR malignan* OR case* OR anomaly* OR malform* OR complication* OR diagnos* OR presentation* OR tumo* OR type* OR neoplasm*)
Health service	health* AND (deliver* OR access*)
Experience	attitude* OR experience* OR perspective* OR satisf*
Population	support* OR famil* OR parent* OR care* OR spouse* OR mother* OR father* OR network* OR sibling* OR patient* OR “service user*” OR customer* OR client* OR “lived experience*” OR people OR person*
Filters	Published after 01/01/2020 English Peer-reviewed research Publication type: Article Document type: Research Article

Search terms and relevant variations (Table 2) included terms like “orphan”, “rare”, “health*”, “deliver*”, “access”, and “experience*”. The search strategy was intentionally broad to capture variations in how RDs are described in the literature.

All study titles, keywords, and abstracts were screened by KMH and TNO against the inclusion and exclusion criteria. Where eligibility was unclear, the full texts were screened according to our protocol guidelines [29] and the two reviewers collaborated to address screening conflicts. Rayyan (Rayyan Systems Inc, 2022) was used to facilitate study screening, including identification and removal of duplicates and resolution of conflicts between reviewers for screened articles. 

For each study, the team identified RDs noted in the reviewed study. Using the Orphanet inventory classification, disease classification hierarchy, and encyclopaedia of rare diseases (https://www.orpha.net/en/disease) [30], the team determined the prevalence of RD subtypes and whether studies, described by the original authors as being about an RD, were recognised more widely as being about an RD. Where at least one condition included in the study could be identified as an RD, the study was included in our review. In some cases, it was not possible to identify the prevalence of the RD studied. This occurred in situations where authors did not specify a particular RD or instead referred to overarching groups of disorders, rather than individual RDs. Reviewed studies may have also referred to RDs by alternative names/ abbreviations, further compounding difficulty in comparing studies. In these cases, where possible, alternative names/ abbreviations were cross-referenced with Orphanet classifications to support documentation of RD prevalence across studies.

**Fig. 1:**
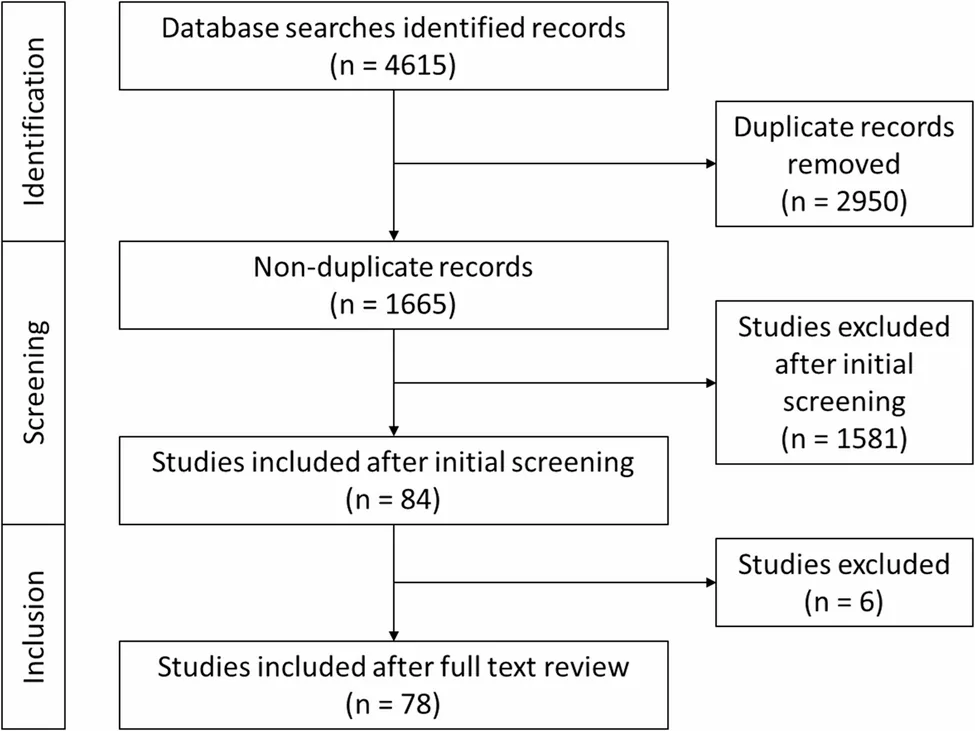
Literature review search, screening, and inclusion process

For a study to be considered for inclusion in the review, it needed to discuss topics related to RDs and access to healthcare or health service delivery, this typically required a detailed examination of the entire document. This thorough review was conducted by TNO and KMH following the conflict resolution stage. Fig. 1 lays out the process of study identification, screening, and inclusion.

### Data extraction and analysis

The data were extracted into and held in a Microsoft Excel spreadsheet (Microsoft Corporation, 2022) after the research team reached consensus on the evaluation criteria. These criteria included the study title, authors, research design, study focus, objectives, participant descriptions (including, for example, age and ethnicity), and outcomes. The research team sought to assess the nature of inclusive methods employed by RD researchers. Research around inclusive methods in RD health research was limited. While there was acknowledgement of the importance of inclusive approaches [31], no study suggested what an inclusive RDs research approach might encompass. Consequently, in assessing limitations of the reviewed studies, we considered participation diversity and engagement (e.g., were research team members living with an RD and was there diversity in the recruited population); data collection and analysis approaches and research design (including whether data collection approaches were appropriate and accessible and whether analysis approaches were clearly reported); and anti-ableist and equity-oriented framing potentially assessed through a study’s theoretical positioning, attention to structural inequities, and how it placed marginalised voices. These principles were developed and drawn from work in learning disabilities^1^ [32, 33] and with disabled children that centres their experiences [34].

GG, KMH, MR, and TNO undertook a first review of the studies. KMH, LY, and TNO then reviewed included studies and analysed them for themes around health service access and delivery, which they discussed to shape preliminary themes. Following discussions, identified themes were systematically refined, examined, critically reviewed, and presented to the broader research team for critique and validation. At this point, BCO independently re-reviewed the data extraction spreadsheet and preliminary thematic structure to identify any omitted themes.

### Policy impact

Countries represented in the included studies were identified. Following this, RD policy presence in these countries was determined using Google (Alphabet Inc.) searches. In addition, general country characteristics (income level [35], health system expenditure [36]) were also recorded. A March 2025 search of the Overton database (Open Policy Ltd.) was also undertaken to identify the policy impact of reviewed studies, i.e., to evidence the contribution the reviewed research had on policy development locally, regionally, or globally. The “search scholarly articles” function was used to identify study citations in policy documents. Each reviewed study was input into this search using the study title and the digital object identifier (DOI). Following identification of this visibility, individual documents were reviewed to understand how the originally cited study informed the policy document (Table 4).

## Results

Seventy-eight publications published between January 2020 and May 2024 were included in our review after full text screening, with a relatively even distribution across the date range. Twenty-one studies were published in the *Orphanet Journal of Rare Diseases* (Springer Nature), with the remainder distributed across a range of journals. Springer Nature published the bulk of reviewed studies (27) across six journals. Elsevier published 13 articles in 11 journals, while Wiley published 12 articles in eight journals.

The review included a diverse range of studies examining healthcare experiences of individuals with RDs and those supporting their care. Thirty studies included the voices of PLWRDs and those caring for them (largely family) [37–66], yet, 27 focused on carer voice without including those living with an RD [67–93]. Few studies included racially, ethnically, or socioeconomically diverse populations, but often featured female and middle-class participants. Studies often explicitly excluded/ did not include children living with RDs or those with learning disabilities [66, 75, 94] or linguistic or language barriers [40, 56, 59, 61, 74, 86, 91, 94–97]. Only one study explicitly reported including Indigenous populations [84]. Similarly, there was only limited consideration of how disability, gender, ethnicity, or rurality influenced RD health service experiences [89, 90, 98–100].

Two Johnston-Devin publications reported findings from the same sample of 17 participants with complex regional pain syndrome [95, 106]. Kerr et al. and Sisk et al. appeared to report partially overlapping vascular-malformation samples, with the same cohort of 24 caregivers apparently included in both studies [51, 88]. Two further publications, Simpson et al. and Walton et al., arose from the CONCORD programme [62, 64]. These represented separate components of the programme, an exploratory interview study and the development of a care-coordination taxonomy, and participant-level overlap was not established. Other publications arose from shared programme-level recruitment settings, including the Rare Dementia Support Impact Study [39, 41, 55] and the Undiagnosed Diseases Network [61, 76].

Nineteen studies focused on PLWRDs as a broad population, this included studies investigating populations with rare neurological disorders, undiagnosed disorders, or rare genetic disorders [38, 45, 48, 54, 61, 62, 64, 66, 68, 72, 73, 76, 81–83, 90, 101–103]. Several studies reported on extremely low-prevalence diseases/ disease subtypes (<1/ 1,000,000), including primary immunodeficiency diseases [67], congenital disorders of glycosylation [69], spinal muscular atrophy [40, 91, 93], 16p11.2 deletion syndrome [80], PIK3CA-related overgrowth spectrum (PROS) [60, 88], congenital ectopia lentis [53], and sarcoma [65]. Studies covered only a minority of RDs; more commonly, hereditary angioedema [50, 104, 105], spinal muscular atrophy [40, 91, 93], rare dementias [39, 41, 63], complex regional pain syndrome [95, 106], lysosomal storage diseases [37, 107], and PROS [60, 88].

Studies showed generally well-reported qualitative methods, though with limitations, particularly around inclusivity (Table 3). Commonly adopted data collection methods included semi-structured interviews, focus groups, and mixed methods studies that combined qualitative and survey-based data. Sample sizes varied from small in-depth studies (the smallest had six participants [78]) to surveys of over 700 respondents [101]. Reliance on support or advocacy groups to recruit participants [40, 43, 51, 55–57, 59, 67, 73, 74, 78, 86, 88, 92, 108, 109] may have introduced selection or engagement biases, while few studies applied methods to mitigate recall bias, such as a longitudinal design [38], video diaries [58], review of social media threads over time [50, 52, 97], or accessing alternative data sources [58, 106].

Some studies described more participatory approaches, such as research-informed poetry [39], creative writing [45], journalling [38], social media thread monitoring [50, 52, 97], drawing [110], or maintaining research advisory/ support groups [38, 45, 60, 62–64, 86, 87, 111] to capture narratives or support analysis. Stamou et al. [63] and Piercy and Nutting [86] adapted data collection methods to participant recall or engagement capacity, Maxfield et al. [109] provided an Easy-English consent form, while Rosenfeld et al. [61] provided plain-language summaries, and Loizidou et al. [55] used a slide presentation with accessible images and text formatting during data collection. Notably, use of research-informed poetry, participatory journalling, and writing demonstrated approaches that enabled diverse expressions of lived experiences, with the authors specifically commenting on the study being an opportunity to trial methods that inform clinician training/ practice [38, 39, 45]. Despite several studies emphasising the value of accessible information sources to support PLWRDs (see later), no studies noted Easy Read translations of research findings.

### Health service access and delivery

A recurrent theme across studies was the prolonged and complex diagnostic process (Table 3), compounding healthcare access issues. Bailey et al. [112] reported that for some, it took up to 16 years to receive a diagnosis for Wilson disease, and Martinussen et al. [81] reported delays of up to 13 years for genetic diagnoses. Azahari et al. [67] identified that the need to outsource tests to overseas laboratories can be a barrier to diagnosis. In turn, PLWRDs and their families described delaying or avoiding healthcare services or treatment following negative encounters/ outcomes or due to symptom discomfort [38, 44, 53, 65, 78, 113]. Often referred to as a “diagnostic odyssey” by either researchers or participants [37, 49, 50, 59, 68, 76, 80, 81, 83, 97], health service access and delivery barriers were typically linked to insufficient frontline clinician knowledge [37, 66, 67, 88–91, 112, 114]. Key aspects of RD health service experiences include service fragmentation; financial and societal marginalisation; and emotional isolation.

**Table 3:** Characteristics of included studies and reported access and delivery experiences

Authors	Study aim	Methods	Sample	Summary of healthcare access/ delivery experiences	Healthcare professionals involved in care delivery	Limitations
Akanuwe et al.	To explore the experiences of people living with Guillain-Barré syndrome in the United Kingdom.	Semi-structured interviews, guided by the Illness Trajectory Framework, were conducted in person or via telephone, depending on participant preference.	Sixteen people with a prior Guillain-Barré syndrome diagnosis. Participants’ ages ranged from 30 to 79 years, with a slight predominance of male participants. The majority were married or cohabitating and had been diagnosed with Guillain-Barré syndrome within the past two years.	Participants highlighted the importance of early diagnosis and timely treatment to prevent complications. Experiences of inpatient care varied, with some participants reporting positive interactions with healthcare professionals, while others described negative experiences, particularly concerning personal care and communication. Delays in treatment were perceived to hinder recovery. The need for ongoing, structured follow-up care post-discharge was emphasised, as many participants felt abandoned once they left the hospital, and others highlighted concerns with accessing disability support.	General practitioners, nurses, physiotherapists, occupational therapists, and speech therapists. The role of these professionals was critical in the initial diagnosis, treatment, and rehabilitation phases. However, participants saw varying levels of support and understanding among healthcare providers, with some professionals demonstrating high levels of care and others being perceived as less supportive.	Small sample size of 16 participants, which may not represent the broader population of people living with Guillain-Barré syndrome in the United Kingdom. The reliance on self-reported data may introduce recall bias, and the lack of objective verification of Guillain-Barré syndrome diagnoses through medical records is noted. The study did not explore the views of healthcare professionals or the impact on family members and caregivers, which could provide a more comprehensive understanding of the experiences of those affected by Guillain-Barré syndrome.
Arora et al.	To understand the impact of insurance delays and denials on hereditary angioedema (HAE) control and healthcare use; well-being, employment, and familial hardship; and steps for people living with HAE to regain access to medication and identify their support needs.	Mixed methods approach, combining an online survey with follow-up focus groups to examine the impact of insurance coverage delays and denials on people with HAE. The survey collected quantitative data on the impact of insurance issues on healthcare utilisation, work/ school attendance, and anxiety levels. The focus groups provided qualitative insights, which were analysed using thematic saturation by three independent reviewers. The participants were recruited from a registry maintained by the United States Hereditary Angioedema Association, and all had experienced insurance delays or denials related to their HAE medication within the past two years.	Twenty participants with HAE, predominantly female (70%), with a mean age of 43.4 years. Most participants had private or employer-based insurance (80%). Nineteen of these participated in subsequent focus groups. The mean age of HAE onset was 10.3 years.	The study found that insurance delays and denials significantly affected healthcare access for people with HAE. These insurance barriers led to increased frequency of angioedema attacks, higher rates of urgent care and emergency department visits, and substantial disruptions to work and school attendance. People with HAE also reported increased anxiety and stress due to the uncertainty of accessing necessary medications. The delays often required participants to undergo step therapy, which involved trying alternative, often less effective medications before being allowed to access their preferred treatment. The study highlighted the frustration and emotional toll associated with navigating the insurance process, including the frequent need for prior authorisations and repeated lab work.	Allergists, primary care physicians, and specialists in HAE noted as supports, but primary focus was on challenges people with HAE faced in interacting with insurance companies rather than the direct role of healthcare professionals in care delivery. The involvement of healthcare providers was often in the context of supporting patients through the insurance process, including providing necessary documentation and engaging in “peer-to-peer” reviews with insurers.	Small sample size based in the United States. The study relied on patient self-reporting, which may be subject to recall bias. Limited applicability to other rare diseases or conditions that also face insurance challenges.
Awada and Holcik	To understand the challenges individuals with lysosomal storage disease (LSD) face in managing their conditions, particularly in navigating the healthcare system, accessing necessary treatments such as orphan drugs, and obtaining social support services.	Semi-structured interviews were conducted with 30 participants across seven Canadian provinces. The interviews were conducted both in person and remotely via video chat, allowing for in-depth discussions. The data were analysed using inductive thematic analysis, following the six phases outlined by Braun and Clarke, to identify key themes and sub-themes related to the experiences of managing LSDs.	Sixteen individuals with an LSD and 14 family members, representing a range of LSDs. Participants varied in age, with some adult patients and others being parents of young children.	Participants reported significant challenges in accessing healthcare services for managing LSD. These challenges included delays in diagnosis, often referred to as the “diagnostic odyssey,” and barriers to accessing specialist care due to the rarity of the diseases. Patients and families frequently encountered a lack of knowledge about LSDs among healthcare providers, which contributed to misdiagnoses and insufficient care coordination. Many participants described the healthcare system as fragmented, requiring them to navigate between various specialists and healthcare providers. Access to orphan drugs was another major challenge, with many participants experiencing long waits or outright denials of treatment due to complex and unclear reimbursement processes. Participants reported significant emotional distress associated with condition management, and limited access to relevant information, disability support, or psychological support.	The study identified a range of healthcare professionals involved in the care of LSD patients, including paediatricians, geneticists, neurologists, and various therapists (e.g., physiotherapists, occupational therapists). However, the involvement of these professionals was often inconsistent, with participants reporting that many healthcare providers lacked the necessary expertise in LSD. The role of treating physicians was particularly crucial in the application process for orphan drugs, but participants noted that some physicians were reluctant to submit drug applications or did not believe the patient, further complicating access to necessary treatments.	Limitations included a relatively small sample size, some Canadian provinces not covered, reliance on self-reported data.
Azahari et al.	To explore and document the knowledge, awareness, and perceptions of genetic testing among parents of children with Primary immunodeficiency disease (PID) in Malaysia. The study sought to understand the challenges these parents face in accessing genetic testing, their understanding of its benefits and limitations, and the overall impact of genetic testing on their families.	Online open-ended, semi-structured focus group interviews, participants were recruited through convenience sampling from the Malaysian Patient Organisation for Primary Immunodeficiencies, a non-governmental organisation dedicated to supporting PID patients and raising awareness about the condition. The focus group discussions were facilitated by a moderator who used a set of predetermined questions to guide the discussions, allowing flexibility for follow-up questions. Thematic analysis was conducted on transcribed interviews to identify key themes related to the participants’ knowledge, awareness, and perceptions of genetic testing.	Fifteen participants (12 mothers and three fathers), all of whom had children diagnosed with PID. The participants were from various regions of Malaysia and had diverse backgrounds in terms of education, ethnicity, and household income. Among the 15 participants, 13 had undergone genetic testing for their children, with five participants also undergoing testing themselves. The reasons for not pursuing genetic testing among some participants included financial constraints, a lack of willingness to know the results, and time limitations.	Participants reported a range of experiences in accessing genetic testing for their children with PID. The study highlighted the limited availability of genetic testing services within Malaysia, necessitating the outsourcing of tests to overseas laboratories for faster more precise testing. This process was often costly and time-consuming, creating significant financial and emotional burdens for families. Additionally, there was a general lack of public awareness and understanding of genetic testing, which further complicated the participants’ ability to access and navigate these services. The interactions with healthcare providers varied, with some participants expressing satisfaction with the support they received, while others faced challenges due to the lack of expertise in PID among local healthcare professionals and described feeling that healthcare professionals lacked empathy and knowledge.	The study identified the role of clinical immunologists in the care of children with PID. These specialists were often the primary source of information and support for families undergoing genetic testing. However, the discussion noted that the limited number of clinical immunologists in Malaysia and the lack of recognition of clinical immunology as a distinct subspecialty by the National Specialist Register posed significant challenges to accessing expert care. The study also highlighted the involvement of other healthcare professionals, including paediatricians and genetic counsellors, in the diagnostic and treatment process. Nonetheless, the need for more widespread knowledge and training in PID among healthcare providers was evident.	The study acknowledged several limitations, including the small sample size of 15 participants, which may limit the generalisability of the findings. The sample was also predominantly composed of mothers, with limited representation from fathers, which may affect the comprehensiveness of the insights gained.
Bailey et al.	To explore and understand the experiences of both patients and physicians in the diagnosis and management of Wilson disease (WD) in the United States. The study specifically aimed to uncover the challenges faced during the diagnostic journey, the role of multidisciplinary care teams, the impact of medication adherence, and the influence of insurance on access to treatment.	Cross-sectional qualitative research design, collecting primary data through one-on-one semi-structured interviews with both United States-based patients diagnosed with WD and physicians specialising in its treatment. The interviews were conducted over the phone, recorded, and then transcribed verbatim. Thematic analysis was employed to analyse the data, with NVivo software used for managing and coding the transcripts.	Twelve patients (seven women and five men) with WD, with an average age of 40 years and an average age at diagnosis of 23 years. The majority of patients were White (83%), and most were receiving care from various hospitals, including Centres of Excellence (COEs) and local medical centres. The physician sample consisted of seven specialists, including hepatologists and neurologists, who had managed patients with WD in the last year. The majority of these physicians were based at COEs.	Patients reported a wide range of experiences with healthcare access, heavily influenced by geographical location, the availability of specialists, the level of their insurance coverage, and family support. The diagnostic journey varied significantly among patients, with those presenting psychiatric or neurological symptoms experiencing the longest delays, sometimes up to 16 years. Access to a multidisciplinary team was identified as crucial for ongoing management, but many patients, particularly those not treated in COEs, faced challenges in accessing such comprehensive care. The study also highlighted issues with medication access, with some patients struggling to afford chelation therapy due to insurance limitations, forcing them to rely on less effective treatment options like zinc without chelators and difficult medication regimens.	The study emphasised the importance of a multidisciplinary approach to managing WD, involving hepatologists, neurologists, psychiatrists, social workers, physiotherapists, and psychologists. While COEs provided access to such teams, many patients treated at local hospitals or by general practitioners reported less comprehensive care. Physicians stressed the importance of regular monitoring and a coordinated care approach to manage the complex symptoms of WD effectively. Patients highlighted issues with clinician knowledge around WD.	The patient sample was not diverse in terms of ethnicity, with a majority being White, which limits the generalisability of the findings across different racial and ethnic groups.
Baptist et al.	To investigate how ageing impacts the frequency and severity of HAE attacks, the challenges associated with managing the condition alongside comorbidities, and the evolving perspectives on life with HAE as individuals age. The research also aimed to identify the specific needs and preferences of older adults with HAE to inform better healthcare practices and support systems.	Focus groups to gather data from participants who were recruited from patient registries at three academic centres. The participants were divided into five focus groups, which were conducted via videoconferencing. The focus group discussions were guided by a pre-developed guidebook, and the sessions were audio-recorded, transcribed, and then analysed using thematic saturation. Three independent reviewers coded the transcripts using NVivo software, with the aim of identifying core themes related to the lived experiences of older adults with HAE.	Seventeen participants, all diagnosed with Type 1 or Type 2 HAE, with a mean age of 65 years. The majority of the participants were female (82%) and White (100%). Participants had been living with HAE for an extended period, with a median age of HAE onset at 14 years and a median age of diagnosis at 35 years. Most participants had experienced multiple emergency department visits due to HAE and were managing their condition with a variety of medications.	Participants reported significant challenges in accessing medications and navigating insurance issues as they aged. Many expressed concerns about the affordability of HAE treatments, especially as they approached or entered retirement and became dependent on Medicare. The study also highlighted difficulties in managing HAE alongside other chronic conditions that commonly affect older adults, such as arthritis and cardiovascular diseases. Some participants noted that their symptoms had worsened with age, requiring more frequent and intensive healthcare interventions.	The study underscored the importance of a multidisciplinary approach in managing HAE, particularly for older adults with multiple comorbidities. Participants interacted with a range of healthcare professionals, including allergists, immunologists, and general practitioners, but the level of expertise and support varied. The study pointed out the need for healthcare providers to be more knowledgeable about HAE and to offer tailored care plans that address the complexities of managing HAE in older adults.	Small, homogeneous sample.
Bogart et al.	To explore the experiences of adults with RDs during the first wave of the COVID-19 pandemic in the United States, focusing on healthcare access, quality of care, psychosocial wellbeing, and coping strategies.	Open-ended questions in a survey distributed through various United States-based RD organisations, social media, and participant contact lists from previous studies. Data collection occurred between May and July 2020. A total of 759 participants responded, representing 231 unique RDs. The data were analysed using conventional qualitative content analysis. English-speaking participants based internationally were also included in the study.	Seven hundred and fifty-nine adults, primarily female (75%) and White (90%), with an average age of 52 years. Participants represented a wide range of RDs, with many having multiple conditions.	Participants reported significant disruptions in healthcare access during the first wave of the pandemic. Many experienced cancelled medical appointments, delayed treatments, and difficulty accessing essential medications due to supply chain issues and changes in insurance coverage. The lack of access to “non-essential” care, such as pain management and mental health services, exacerbated symptoms for many individuals with RDs. Some participants also expressed fear of visiting hospitals due to the increased risk of COVID-19 exposure, further limiting their access to necessary healthcare. However, the expansion of telehealth services was noted as a positive development by some, as it allowed participants to maintain contact with their healthcare providers without travel.	The study highlighted the involvement of various healthcare professionals, including specialists in rare diseases, general practitioners, and mental health providers. However, participants noted that the pandemic exposed gaps in the healthcare system’s ability to support individuals with RDs, particularly in terms of specialist care and coordination between different providers. The reliance on telehealth, while beneficial for some, also presented challenges for those requiring in-person assessments or treatments. The study underscored the importance of ensuring that healthcare professionals are equipped to manage the unique needs of RD patients, especially during crises like the COVID-19 pandemic.	Overrepresentation of White, female, middle-aged, and middle-class individuals in the sample. Data were collected during the early stages of the pandemic, capturing a specific moment in time that may not reflect evolving pandemic experiences.
Bush et al.	To explore the experiences of families caring for children diagnosed with conditions identified through newborn screening programmes. The primary focus was on understanding the implications of these experiences for the future expansion of genomics in population-based neonatal public health programmes. The study sought to inform ethically nuanced policies that could guide the integration of more comprehensive genomic technologies in newborn screening.	This qualitative study utilised in-depth interviews with 169 family members from 77 families. Participants were recruited through clinical studies, family meetings, and advocacy groups related to rare genetic conditions. The interviews were analysed using thematic analysis to identify key themes related to the diagnostic journey, medical management, and psychosocial implications of caring for children with conditions identified through newborn screening.	One hundred and sixty-nine individuals, primarily parents, with an average age of 43 years. Most were female (66.9%) and married (82.2%), with a significant proportion holding a bachelor’s degree or higher. The children in question were on average ten years old, with most diagnosed with organic acidaemia. The demographic diversity of the sample was limited, with the majority of participants identifying as White (84%) and non-Hispanic (90%).	Participants described the diagnostic journey as an “odyssey,” marked by uncertainty and challenges in understanding their child’s condition. The study highlighted the difficulties participants faced in accessing timely and accurate diagnoses, compounded by the rarity and variability of the conditions. There was often a lack of medical knowledge among healthcare professionals, leading to delayed or incorrect diagnoses. Families struggled to navigate a complex healthcare system, requiring them to become advocates for their children. Access to multidisciplinary care was deemed crucial but often difficult to obtain, particularly in geographically isolated areas.	The study identified the need for better training and resources for healthcare professionals involved in the care of children with newborn screening-related conditions. Paediatricians, geneticists, and other specialists were central to the diagnostic and ongoing management process. However, the limited number of specialists and the variability in provider expertise often hindered effective care. The study emphasised the importance of patient-centred communication and the role of healthcare providers in supporting families through the diagnostic and treatment process.	Lack of sample diversity; demographic skew may limit finding generalisability.
Byun et al.	To understand how the rapidly changing environment during the COVID-19 pandemic impacted the lives of individuals living with genetic, undiagnosed, and rare diseases. It focused on assessing the pandemic’s effect on their wellbeing, resilience, and access to health and social care services. The study also sought to identify lessons that could inform future health and social care provisions for this vulnerable population.	Phenomenological, longitudinal qualitative research design, collecting data from open-prompted journals submitted by participants from July 2020 to May 2021. The study was co-designed and led by a patient support network, and participants were recruited through patient support group websites and social media. Data analysis was conducted using a deductive approach based on the Resilience Scale for Adults and an inductive thematic analysis to capture the evolving experiences of participants over time. Participants had flexibility in how they completed their journalling; submissions were handwritten, typed, or sung, and also included photographs or graphics.	Seventeen journals received upon first submission, these participants continued to contribute throughout the study. Participants were individuals living with genetic, undiagnosed, or rare diseases, caregivers, or support-group leaders. Participants were primarily from metropolitan areas, particularly Victoria, which experienced the most stringent COVID-19 restrictions. The sample covered a broad age range, with most participants aged between 46 and 59 years.	Participants reported significant healthcare access disruptions during the pandemic, with many facing delays or cancellations of essential health and social care services. The fear of contracting COVID-19 led some to avoid seeking care, further complicating their health management. The shift to telehealth was beneficial for some but challenging for others, particularly those with specific accessibility needs. The study highlighted the inconsistencies in healthcare delivery across different states, with more severe impacts noted in regions like Victoria that had stricter lockdown measures.	Healthcare professionals involved in the care of participants included a range of specialists and general practitioners. However, the study noted gaps in service provision, particularly in mental health and social care, which were often curtailed due to the pandemic. The need for healthcare providers to be adaptable and responsive to the unique needs of individuals with rare diseases was emphasised, especially in maintaining continuity of care during crises.	Small, homogeneous sample, which may not fully represent the diverse experiences of the broader genetic, undiagnosed, and rare disease community. The focus on a specific timeframe during the pandemic may not capture long-term impacts or variations in experiences across different regions and disease types.
Camic et al.	To explore and understand the lived experiences of people affected by rarer forms of dementia through the use of co-constructed research poetry. It also sought to evaluate the effectiveness of poetry as a tool to convey these experiences to healthcare professionals, with the goal of enhancing empathy, critical thinking, and understanding among those providing care.	Qualitative data collection from 71 individuals living with rarer forms of dementia and their care partners, who co-constructed 27 poems with professional poets. The data were collected through online short qualitative surveys, focus group interviews (with one person living with a rare dementia, and with nine care partners), and the analysis of participants’ contributions to the poetry. Healthcare professionals were also surveyed (online mixed methods) to assess their responses to the poetry and its potential as an educational tool. Thematic narrative synthesis was employed to analyse the qualitative data, and the study followed a stepwise approach to the co-construction of the poems.	Seventy-one participants, consisting of 27 people living with rarer forms of dementia and 44 care partners (including five bereaved caregivers), ranging in age from 36 to 82 years. Most participants were Caucasian. Ninety-three healthcare professionals (including in training) participated in the survey.	This study underscored the brutality and rollercoaster ride of having and navigating a rare dementia. Participants reported significant challenges in obtaining an accurate diagnosis and relevant care for their conditions. The rarity of their dementia types often led to misdiagnosis and misunderstanding by healthcare professionals. The lack of recognition and appropriate support pathways for these rarer forms of dementia contributed to a continuous struggle for patients and their care partners. The study highlighted the emotional and social impacts of these challenges, including feelings of isolation, frustration, and the need to adapt continuously to the changing symptoms and care requirements.	The study underscored the involvement of a range of healthcare professionals, including general practitioners, neurologists, and dementia specialists. However, it also pointed out the gaps in knowledge and understanding among healthcare providers regarding rarer forms of dementia. The research poetry revealed that healthcare professionals often lacked the necessary expertise to provide adequate support, leading to misdiagnoses and insufficient care, which perpetuated a sense of being alone. The study suggested that increased education and training are needed to improve the understanding and management of these conditions.	The subjective nature of poetry interpretation may also introduce potential biases. Furthermore, the study’s focus on a specific set of dementia types and the involvement of participants primarily from Western countries may not fully capture the experiences of individuals with other rarer forms of dementia or those from different cultural backgrounds. Participants needed to have Internet access to participate.
Cardão et al.	To explore the experiences of parents raising children with congenital disorders of glycosylation (CDG), focusing on their needs and challenges in managing care.	Semi-structured interviews conducted via Skype with 33 parents from 11 countries, exploring diagnostic journeys, disease management, emotional and financial challenges, and coping strategies. Participants were recruited through a patients’ association and interviews were conducted in a range of languages (not always native to the participant). Thematic analysis was employed to identify key themes from the transcripts.	Thirty-three parents from 11 countries. The children had a range of CDG subtypes, including Phosphomannomutase 2-CDG and less common forms like CDG of the DPAGT1 gene.	Parents described long delays and uncertainty in receiving a CDG diagnosis due to lack of medical awareness. They frequently encountered misdiagnoses and struggled with finding knowledgeable health providers. Many had to travel to multiple cities and consult several specialists before receiving a diagnosis, or faced care barriers due to insurance access. Post-diagnosis, parents reported difficulties accessing coordinated care and managing the complex needs of their children, they highlighted the value of support from family (and other support networks) and the patients’ association. Health providers often lacked specific knowledge about CDG, leading to fragmented care, and parents had to assume the role of medical experts. Parents also noted barriers to support for themselves, including lack of access to mental health support, or time/ energy to access such services.	Paediatricians, neurologists, geneticists, and various specialists were involved, but there was poor communication and coordination between them. Many healthcare professionals lacked knowledge of CDG, resulting in inadequate guidance and support for families. Some parents reported positive experiences with specific health teams that were patient-focused and well-informed.	The study was limited by the focus on parents who were members of CDG associations, which may not represent the broader population. Most participants were mothers, and some interviews were conducted in non-native languages.
Castro et al.	To explore the day-to-day experiences of families who are caring for children with osteogenesis imperfecta (OI), focusing on managing daily care, challenges, and strategies.	Qualitative descriptive design; the research was conducted at a paediatric orthopaedic hospital affiliated with a university in Montreal, Canada. Data were collected through individual semi-structured interviews, focusing on their daily caregiving routines and the challenges they faced. Interview transcripts were thematically analysed.	Eighteen adult family members (mainly mothers) of children (mainly aged between 6 and 12 years) with OI from 14 families covering 17 children in total. Children had OI types 1, 3, 4, or 6.	Participants reported that their access to healthcare was significantly influenced by the availability of specialised OI treatment centres. Those who were able to connect with such centres felt better supported and more confident in managing their child’s condition. However, accessing these resources often required considerable effort, including navigating long travel distances, coordinating care with multiple providers, and educating local healthcare professionals who lacked expertise in OI. The study highlighted the difficulties faced in managing care for a child with OI during periods of increased need, such as following fractures or surgeries, where the complexity of care delivery intensified.	Paediatricians, orthopaedic specialists, physiotherapists, and occupational therapists. The role of these professionals was critical in acute management of fractures and ongoing care and rehabilitation of children with OI. Participants expressed a need for healthcare providers to be well-informed about OI to ensure that care was appropriate and effective. However, the study also revealed gaps in the knowledge and availability of specialised care, particularly in local healthcare settings, which often required families to educate healthcare providers.	Small sample, primarily consisting of mothers, which may not fully represent the diversity of caregiving experiences. The study was also conducted at a single specialised treatment centre, which may limit the generalisability of the findings to other settings. Additionally, the reliance on self-reported data from participants introduces the possibility of recall bias.
Ch’ng et al.	To explore the impact of living with spinal muscular atrophy (SMA) on the lives and daily activities of SMA patients and their caregivers in Malaysia.	A mixed method, cross-sectional study using a self-administered questionnaire followed by in-depth interviews and focus group discussions. Thematic analysis was used for qualitative data, and grounded theory principles guided coding. Participants were recruited through a local SMA advocacy organisation.	Forty-two participants: 13 people with SMA and 29 caregivers. Participants came from different ethnic groups, with the majority being Malay. The SMA types included Type 2 (69%) and Type 3 (31%) among those with SMA, and Type 1 (28%), Type 2 (38%), and Type 3 (34%) among the people participating caregivers looked after.	Many patients and caregivers experienced delayed diagnosis, misdiagnosis, and insufficient information from healthcare professionals. Most reported limited access to supportive services such as physiotherapy. The psychological burden was substantial, with caregivers particularly noting feelings of stress, anxiety, and depression. People with SMA faced challenges with independent mobility, personal hygiene, and daily tasks. The lack of infrastructure and services for disabilities further compounded these difficulties. Both caregivers and patients expressed frustration with the fragmented healthcare system and limited government support.	Paediatricians, neurologists, and physiotherapists were involved in care. However, communication between specialists was poor, and a lack of knowledge about SMA among healthcare providers contributed to delays in diagnosis and care. There was also a call for better palliative care services and counselling for caregivers.	Small sample size, limited to Malaysia, which may affect generalisability. Participants were recruited via a local SMA advocacy organisation, which may introduce selection bias. There was also a lack of genetic confirmation for some participants, and recall bias could influence the qualitative data.
Costa et al.	To investigate the early feeding-related experiences of parents of children with craniofacial microsomia (CFM), a condition associated with feeding difficulties.	Remote narrative interviews. Participants were recruited through craniofacial clinics, social media, and advocacy events in the United States. Narrative interviews allowed parents to share their experiences with minimal intervention from interviewers, meaning participants could focus on the topics most important to them. The interviews were conducted in English or Spanish, depending on the participant’s preference. Data were then transcribed and analysed using reflexive thematic analysis. Two researchers with psychology backgrounds conducted the coding process, ensuring reliability through regular discussions to refine and clarify themes.	Thirty-five United States-based parents of 34 children with CFM. Diagnoses included hemifacial microsomia, Goldenhar syndrome, and other CFM-associated features.	Parents faced significant emotional and practical challenges related to feeding. Many reported distress when breastfeeding was not possible, leading to negative emotions such as guilt and shame. Some parents had difficulty accessing reliable feeding support or had poor communication with healthcare providers. Feeding adaptations, such as formula or tube feeding, were common but required parents to learn new skills and often led to isolation.	Lactation consultants, paediatricians, neonatologists, and feeding therapists were involved, but there was limited support for alternatives like formula feeding. Many parents felt unsupported when breastfeeding was not successful, and there was a need for better coordination between healthcare professionals.	Small sample size and reliance on parents’ narratives, which may introduce recall bias. The study was limited to the United States and may not generalise to other populations. Fathers were underrepresented.
Currie and Szabo	To explore the experiences of parents caring for children with rare neurodevelopmental disorders (NDDs) and the challenges they face in navigating medical, social, and family systems.	Hermeneutic phenomenology was used. Fifteen parents were recruited via purposive sampling from medical genetic, endocrine, and neuropsychiatry clinics and through parent support groups. Data were collected using semi-structured interviews, which were transcribed and analysed.	Fifteen parents (11 mothers and four fathers) with children aged 11 years and under, all diagnosed with a rare NDD in the first two years of life. The sample included both urban and rural residents from Western Canada, with a range of socioeconomic statuses. Parents were included if their child had a NDD with a prevalence of fewer than 1 in 2,000 live births. All children had two or more comorbidities as a result of their NDDs.	Parents described significant challenges navigating fragmented health and social care systems. They encountered delays in diagnosis, lack of coordination between care providers, and a general lack of understanding about rare NDDs. Many felt overwhelmed by the complexity of their children’s care, which required constant interaction with various medical systems. Social stigma and misunderstanding were common, contributing to isolation. Parents also felt emotionally drained by the continuous demands of caregiving and the lack of sufficient support, both from healthcare systems and their social networks.	A wide range of healthcare professionals were involved, including paediatricians, geneticists, neuropsychiatrists, endocrinologists, nurses, and social care workers. However, parents noted that care was often fragmented due to poor communication between specialists. Paediatricians and geneticists were involved in diagnosing and treating the rare NDDs, while neuropsychiatrists helped manage behavioural and cognitive issues. Nurses and social care workers provided support but were often limited in their ability to offer comprehensive care. The lack of collaboration and coordination across these disciplines was a major barrier to effective care.	The study had a small sample size and was limited to Western Canada, which may affect the generalisability of the findings. In addition, the sample may not fully represent the diversity of experiences across different socioeconomic backgrounds or regions.
Davies-Abbott et al.	To explore the diagnostic pathways experienced by people affected by rare dementia in Wales, United Kingdom, and their family support. The study also examined the practical, emotional, and economic consequences of the diagnostic process.	Mixed methods design, combining semi-structured qualitative interviews with thematic analysis and economic analysis using a bottom-up costing approach.	Ten participants total: (aged 42-85 years) nine family support people and one person living with rare dementia participants included individuals with frontotemporal dementia, primary progressive aphasia, and posterior cortical atrophy.	The diagnostic journey was fragmented, with many participants describing significant delays, inappropriate referrals, and disputes between healthcare providers. Private healthcare was used by some to expedite the diagnosis due to long waiting lists. Many participants felt excluded from the process and noted emotional burdens such as frustration and anxiety. Family members often had to “fight” for appointments, which increased their emotional and financial burden. Miscommunication and disputes about diagnosis were also common.	General practitioners, community mental health nurses, neurologists, psychiatrists, and psychologists were involved in the diagnostic process. The need for clearer referral pathways and better collaboration between specialists and primary care was highlighted.	The small sample size (ten participants) and involvement of family limits the generalisability of the findings. All participants were White British, further limiting the applicability of results to more diverse populations.
de Dios García‑Díaz et al.	To explore the impact of LSDs on the lives of patients in Spain and their journey from diagnosis to follow-up, while identifying unmet needs perceived by patients and healthcare professionals.	Semi-structured, face-to-face in-depth interviews with patients and healthcare professionals. A purposive sampling method was used to ensure diversity in patient backgrounds, treatments, and regions across Spain.	Twenty patients with LSDs (five each with Fabry disease, Gaucher disease, Pompe disease, and mucopolysaccharidosis Type 1) and 25 healthcare professionals from different regions in Spain. The sample included treated and untreated patients from both rural and urban areas.	Patients reported a significant impact on daily routines, including emotional challenges, work/ school difficulties, and social life disruptions. The pre-diagnosis stage was the most negatively perceived due to delayed diagnosis and misdiagnosis, while the follow-up stage had fewer negative perceptions. Family dynamics were often strained, particularly when managing symptoms like fatigue, and patients frequently faced misunderstandings from those unaware of their condition. Hospital dependency was high, and the burden of attending regular medical visits interfered with personal and family life. Uncertainty regarding treatment outcomes was also a source of anxiety.	Internal medicine specialists, nephrologists, cardiologists, haematologists, neurologists, paediatric neurologists, and social care professionals. A multidisciplinary approach was considered crucial due to the multi-organ nature of LSDs.	Small sample size, limited to Spain, which may affect the generalisability of the findings. The qualitative nature may introduce response bias. Patient selection by clinicians could result in bias in the representation of experiences.
Denton et al.	To explore the communication needs of patients with systemic sclerosis and interstitial lung disease (SSc-ILD), specifically highlighting important topics often omitted from consultations.	In-depth individual interviews and observed conversations between patients, caregivers, physicians, and nurses.	Twenty-one patients with diagnosed SSc-ILD, 42 physicians, 16 specialist nurses, and five caregivers participated in the study.	Prognosis, mortality, and disease progression were rarely discussed, leaving patients uncertain about their future. Many patients and caregivers felt afraid or uncomfortable asking about these issues. Patients also refrained from asking about daily life concerns such as family, work, and relationships, fearing these were outside the physician’s scope or due to time constraints during consultations. This created frustration and anxiety. Physicians often focused on immediate medical concerns, avoiding long-term discussions to prevent distress. Specialist nurses were seen as more approachable for discussing daily life and emotional concerns, though they lacked detailed knowledge to provide full support.	Physicians (rheumatologists, pulmonologists), and specialist nurses (rheumatology, dermatology, pulmonology) were involved. Nurses had more time but lacked sufficient knowledge for full support.	Small sample size, limited to Western populations. The findings may not generalise to a broader population. Some participants felt uncomfortable asking certain questions, which may have affected data collection.
Diaz et al.	To assess health insurance literacy and perceived challenges in accessing health services, treatments, and medications among Niemann–Pick disease (NPD) patients and parents/ guardians. The aim was to identify healthcare access gaps and inform supportive initiatives.	Qualitative prospective observational cohort study. Data were collected via an online quantitative survey to measure health insurance literacy, followed by structured telephone interviews exploring perceptions of access challenges.	Seventy-nine respondents from the Niemann–Pick disease community. The sample was composed mainly of parents/ guardians responding on behalf of patients, with subtypes of Niemann–Pick disease, including acid sphingomyelinase deficiency and NPD Type C. Of the 79 participants, 51% represented people with childhood-onset NPD Type C, 19% had adult-onset NPD Type C, and 30% had acid sphingomyelinase deficiency.	The greatest barrier to accessing care reported was the process of obtaining medical care, treatment, and services. Respondents mentioned frequent delays and denials of essential medications and services, contributing to disease progression. Parents/ guardians often experienced burnout, and the uncertainty of future access to experimental treatments exacerbated these challenges. Mental health and physical symptoms were substantially affected by these access barriers, with patients experiencing heightened stress, fear, and worsening physical conditions.	Multidisciplinary teams including hepatologists, neurologists, pulmonologists, endocrinologists, physical therapists, and geneticists. Care delivery involved supportive and palliative care due to lack of curative treatments.	Potential selection bias as participants were mainly members of National Niemann–Pick Disease Foundation. Reliance on parents/ guardians as proxy respondents may not fully capture patient experiences. Self-reported data could introduce recall bias.
Fitzgerald et al.	To explore the parental experience of having a child with a rare genetic condition, focusing on parental interpretation, adaptation, and coping with ambiguous genetic findings.	Semi-structured interviews via telephone or in person exploring the intrapersonal and interpersonal experience of parents of children with a rare genetic diagnosis. Deductive thematic analysis undertaken on interview transcripts. Recruitment via an existing database and via a charitable organisation.	Thirty individuals (24 mothers and six fathers; age range 23 to 62). 21 in a relationship, five separated/ divorced. Children (*n* = 26) ranged from infancy to adolescence. Conditions included chromosomal abnormalities and developmental delays; some families had more than one affected child. Diagnoses not specified, but prevalence < 1% population frequency and less than 1 in 2,000 live births was inclusion criteria (19 children received diagnoses in early childhood). Four children of participants had reached adulthood at the time of interview.	Parents reported significant emotional distress during the diagnostic process, with some experiencing relief and validation after long waits while others faced grief and uncertainty. Participants noted feeling they had a lack of power or control given the permanence of diagnoses. For some there was self-blame, while others felt that they were ‘different’ from the general population. Participants noted the power of clinicians to reassure, but also to lack compassion. The uncertainty of prognosis, lack of a roadmap (juxtaposed against the space to sit with uncertainty), and the need for knowledge in the face of a rare diagnosis was challenging. Participants described personal and professional isolation (although having other families as emotional support), clinicians lacked knowledge/ understanding to support, definitive language caused distress, and lack of connection to others. Further, participants noted priorities changing as the child took focus and parents became hypervigilant.	Paediatricians, geneticists, and other specialists. Parents identified a lack of coordinated care across different medical professionals. Lack of mental health support to cope with the uncertainty of an RD/ diagnosis.	Gender imbalance (most participants were mothers). The study may be affected by recall bias due to its retrospective nature, and the findings may not generalise beyond the Irish healthcare system. The socioeconomic diversity of participants was not fully explored, limiting broader applicability.
Gerstein et al.	To explore decision-making regarding medical management versus liver transplantation for children with urea cycle disorders (UCDs), aiming to identify factors influencing family choices.	Adapted grounded theory approach using semi-structured interviews of parents and clinical service providers. Following this, the researchers conducted two in person parent focus groups. Parent recruitment involved social media contact through national foundations, listservs and one-on-one outreach. Provider recruitment occurred in a similar manner but via a UCDs consortium. Data underwent thematic content analysis.	Parents of children born in the United States after 1996, who had a UCD. 35 interview participants, and 19 in the focus groups. Parents were predominantly female and aged between 30 and 39 years old. Most had children with neonatal onset UCD. Twenty-six clinical providers interviewed.	Participants noted insufficient evidence and poorly defined clinical guidelines impaired the ability to make choices in managing UCDs. Parents identified a tipping point where they could no longer manage their child before seeking liver transplant, but also a sense of mastery if able to manage daily challenges with medical management. Families faced significant challenges, including barriers accessing specialised metabolic care; unpredictability of hyperammonaemic episodes required frequent hospitalisations, placing substantial emotional and logistical burdens on parents. Accessing medical management resources was often hindered by high cost/ bureaucratic obstacles, while liver transplantation, though typically fully insured, presented risks and uncertainties. Interactions with healthcare providers varied; some families experienced supportive care and others encountered lack of expertise or clear recommendations. Parents often relied on peer networks for information to navigate complex decisions under high stress and uncertainty.	Metabolic specialists, physicians.	Study merged views of providers and parents (recruitment occurred through specific advocacy and other networks); underrepresentation of non-English-speaking/ non-affluent families; exclusion of families who lost children to UCD-related complications.
Gill et al.	To explore the patient and caregiver (parent, partner, or close friend) burden of acute hepatic porphyria (AHP) based on frequency, considering acute attacks, chronic symptoms, and impact on quality of life, work, and social and family life.	Mixed methods study involving an online survey of British Porphyria Association and/ or Porphyria United Kingdom members, followed by in-depth interviews (ten patients, three caregivers). Thematic analysis, based on a predefined coding framework, and descriptive statistics were employed to analyse qualitative and quantitative data.	Thirty-eight patients (95% female) and ten caregivers (70% female) participated; patients were aged between 24 and 57 years. All interviewed patients had experienced at least one AHP attack in the past two years. Interviewed participants had experience with acute intermittent porphyria or variegate porphyria.	Some participants reported a lack of recognition of AHP in emergency settings and the need to go elsewhere to specialist support; variable access to treatment (e.g., haeme arginate) and notably, adequate pain relief was not able to be achieved by every patient due to treatment tolerability issues. Many patients expressed reluctance to seek care due to previous poor experiences, and there was a lack of consistency on what constituted a severe attack. Participants noted that AHP affected their daily lives, particularly in those with frequent attacks and the role of family/ relationships. For example, participants noted the need for long or frequent hospitalisation/ sick leave and changes to working ability.	Not specified.	Small sample size for those living with AHP and their caregivers. Self-reported data on type of AHP/ AHP details unverified by medical records; recruitment from advocacy groups may limit diversity of experiences.
Gorman and Farsides	To explore the everyday lives of people living with rare genetic conditions and how their lives are influenced by the evolution of fields such as genomic medicine. To also investigate how participatory-writing can help highlight the impacts of rare genetic conditions.	Participatory qualitative study involving creative writing workshops with families affected by rare genetic conditions. Reflective interviews were conducted following workshops, with a focus on the process and content of writing. Content analysed using dialogical narrative analysis.	Families (people who had served as patient representatives on genomic ethical and governance panels/ who were active in patient-led organisations; initial participants made suggestions for others to join); the group included participants with active caregiving responsibilities and prior involvement in genomics research projects (e.g., 100,000 Genomes Project). The first writing group had five participants; seven participants were in the second group.	Caring for someone with a rare genetic condition came with hidden responsibilities, this could involve a lack of alignment between family experiences and clinicians’ focus. Accessing genomic medicine services could require quickly learning scientific vocabulary to interpret clinical communications and confidence in understanding, participating and obtaining optimum care.	Focused on healthcare professionals in general, with mention of genetic counsellors, and physicians.	Limited explanation of sampling, and limited synthesis of findings; unclear how data were interpreted for presentation and little shared understandings/ analysis presented. Participants recruited via advocacy networks, which may not reflect all family experiences; focus on writing may deter participation from individuals uncomfortable with the medium.
Güeita-Rodriguez et al.	To describe the barriers to accessing care resources experienced by parents of children with Rett syndrome.	Qualitative case study with in-depth interviews (*n* = 19), two focus groups (12 parents), researchers’ field notes, and parents’ personal documents. Thematic inductive analysis was conducted.	Thirty-one parents (17 women, 14 men) of children with Rett syndrome, comprising 19 families.	High cost of genetic tests; limited access to hospital care, services and resources; private costs or differential/ lack of access to public healthcare in the home; delays in diagnosis; poor awareness of Rett syndrome among primary care providers; bureaucratic obstacles when accessing social support and treatments; regional disparities in service provision. Participants also focused on the issue of time to enable care, effective treatment, and continued daily functioning e.g., to work.	Physicians, geneticists, physiotherapists, occupational therapists, speech therapists, and social workers.	Limited generalisability. Children excluded from interviews; both parents not always included; physicians did not participate.
Halbach et al.	To explore male breast cancer (MBC) patients’ perspectives on their healthcare experiences from diagnosis to aftercare.	Mixed methods study. Postal survey (questionnaire) and semi-structured interviews. Analysis used descriptive statistics and qualitative content analysis.	German MBC patients via self-help network for MBC patients “Männer mit Brustkrebs e.V.” and public notices in the media between 2016 and 2017. 100 surveys were analysed (117 recruited), 27 survey participants were interviewed. Participants were aged 39–89 years (interview participants aged 42–89 years), with a mix of treatment histories including surgery (all received), chemotherapy, radiation, and hormone therapy.	Diagnostic phase problematic due to low awareness among primary care physicians; access challenges to gynaecology services due to gender; stigma, delayed diagnosis, and lack of awareness among healthcare providers. Treatment-related uncertainties (e.g., tamoxifen use); lack of clarity in aftercare responsibilities.	Primary care physicians, gynaecologists, oncologists, surgeons, and radiotherapists.	Variability in years since diagnosis; results may be unique to German healthcare system.
Halley et al.	To characterise perceived utility and disutility of genomic sequencing among parents of children enrolled in the Undiagnosed Diseases Network, with a focus on diverse sociodemographic perspectives.	Qualitative, comparative content analysis of in-depth interviews (*n* = 30). Interviews included questions about diagnostic odyssey, experiences with genomic sequencing, and perceived impacts on health, psychological well-being, and social connections.	Thirty parents (90% female, 43.3% Hispanic, 33.3% Spanish-speaking). Children were enrolled in the Undiagnosed Diseases Network for genomic sequencing to investigate undiagnosed rare diseases.	The study highlighted intersectionality of health and sociodemographic disparities in rare disease care. Variability in perceived benefits and challenges accessing genomic technologies. Parents highlighted changes to specialised healthcare support and clear communication. Issues included access to social support and research opportunities, insurance, genomic sequencing helped with access to healthcare; clinical recommendations, insurance reimbursement, communication with healthcare providers and qualification for therapeutic services all influenced by genomic sequencing.	Physicians, geneticists.	Genomic sequencing provided in context of research rather than clinic, recruitment limited to participants already engaged in genomic sequencing studies. Small, non-representative sample; design was exploratory.
Hitchen et al.	To explore the challenges, emotional wellbeing, and lived experiences of parents raising a child with Goldenhar syndrome.	Cross sectional qualitative study involving semi-structured interviews with parents recruited at a United Kingdom Goldenhar syndrome conference. Researchers adopted a direct-realist methodology and analysis followed a reflexive thematic analysis approach.	Ten parents (five mothers, five fathers), aged 34–51 years, with children aged 3–19 years. All parents were married, and children had varying severities of Goldenhar syndrome.	Parents reported lack of information at diagnosis, emotional distress, and medical professionals’ limited knowledge of Goldenhar. Parents experienced long healthcare journeys with multiple appointments and procedures, but saw value in peer support. Participants highlighted different journeys based on gender, and feelings of isolation.	Multidisciplinary teams, noting issues with knowledge around the condition.	Small, retrospective sample; only married parents participated; parental perspectives may change over time, which cannot be fully captured in cross-sectional interviews.
Hoffman et al.	To describe the diagnostic and treatment journeys of patients with adult growth hormone deficiency (AGHD) from their perspective, identifying unmet needs and gaps in diagnosis, treatment, and healthcare processes.	Semi-structured virtual interviews and a virtual advisory board meeting. Nine patients participated (three with childhood-onset AGHD and six with adult-onset AGHD). Authors further observed and recorded patients’ experiences noted during the advisory board meeting.	Nine patients: three with childhood-onset AGHD and six with adult-onset AGHD; patients were identified through patient networks or advocacy groups. Diagnoses for adult-onset AGHD patients included pituitary adenoma, brain injury, Cushing’s syndrome, and other pituitary deficiencies.	Delays in diagnosis due to nonspecific symptoms, multiple consultations with specialists, and lack of awareness among clinicians; testing for AGHD often required advocacy from patients. Treatment delays due to insurance barriers were common. Participants reported having to research and advocate for themselves and described being doubted by medical professionals.	Endocrinologists (primary specialists), general practitioners, neurologists, rheumatologists, and other specialists involved during the lengthy diagnostic process.	Small sample size (*n* = 9); experiences limited to the United States; selection bias due to reliance on advocacy groups for recruitment.
Hoffmann-Vold et al.	To identify unmet needs in the pre-diagnostic, diagnostic, and post-diagnostic phases of the patient pathway for SSc-ILD, as experienced by patients and healthcare professionals.	Semi-structured interviews in eight European countries with physicians, paramedical groups, nurses, patients, and caregivers using four sets of 70 research questions. Pre-diagnostic, diagnostic and post-diagnostic phases of patient pathway were systematically explored.	Ninety-five healthcare professionals (including rheumatologists, pulmonologists, nurses, and paramedicals) and 47 patients and caregivers; patients were recruited from specialised centres. Note descriptions of participant numbers differ throughout the study and variability between countries as to which cohorts participated.	Significant delays in diagnosis; lack of disease knowledge among general practitioners and specialists; poor referral pathways; insufficient supportive care; difficulties obtaining clear, disease-specific information or connection to others (healthcare and support from others with SSc-ILD).	Rheumatologists (primary specialists), pulmonologists, dermatologists, general practitioners, nurses, physiotherapists, occupational therapists, and psychologists. Lack of specialist nursing and paramedical care noted as a limitation to the patient care pathway.	The authors listed the qualitative nature of study as a limitation. Recruitment methods may have resulted in bias. Few general practitioners involved. Underrepresentation of patients.
Ihne-Schubert et al.	To identify treatment-relevant needs of amyloidosis patients, their relatives, and healthcare professionals, and to develop an amyloidosis-specific care approach.	Mixed methods approach with three steps: (1) focus groups with patients, relatives, and healthcare professionals, (2) questionnaire-based quantitative survey, and (3) development of a needs-adapted care concept. Recruitment for steps one and two occurred via the Interdisciplinary Amyloidosis Centre North Bavaria of the University Hospital of Würzburg. Results presented relate to step one only.	Focus groups: seven patients (mean age 67), six relatives, and five healthcare professionals (general practitioners, cardiologists, nephrologists, haematologists). Participants were diverse in age, gender, and amyloidosis subtypes (systemic light chain, hereditary transthyretin, secondary amyloidosis).	Long delays in diagnosis due to lack of awareness and clinical competence among local healthcare professionals; need for a centralised care model where specialised centres coordinate treatment (with improved communication), monitoring, and psychosocial support and companionship during medical visits.	General practitioners, cardiologists, nephrologists, haematologists, need for psychosocial support staff (e.g., psychologists and social workers) described, and specialised amyloidosis centres.	Single-centre study; small focus group size; possible selection bias from recruitment approach.
Inhestern et al.	To investigate patient and caregiver experiences of intersectoral and interdisciplinary care, focusing on collaboration between local healthcare professionals and rare disease expert centres in Germany. Specifically, the study focused on healthcare experiences, perceptions on collaboration and cooperation, and satisfaction.	Mixed methods design, including a cross-sectional survey and semi-structured interviews (amongst those who participated in the survey). Quantitative analysis used descriptive statistics; qualitative analysis employed content analysis. Recruitment initially occurred through rare disease centres.	A total of 299 participants completed the survey: 176 patients and 123 caregivers of children. Overall, 66% were female and caregivers were on average younger than patients. Fifty survey participants subsequently completed interviews. Diseases included rare metabolic, connective tissue, and musculoskeletal disorders, including Marfan syndrome and oesophageal atresia.	Long delays in diagnosis, insufficient knowledge among healthcare professionals, and barriers to accessing specialised care. Patients often responsible for coordinating communication between providers, leading to significant psychosocial strain. Participants reported that changing clinicians (i.e., lack of continuity) resulted in loss of information and lack of clinician responsibility, and that the form of communication (often in writing) led to additional delays.	Specialists at rare disease centres, primary care physicians, and allied healthcare professionals (e.g., physiotherapists).	Recruitment predominantly from pre-selected centres with established practices for intersectoral collaboration; fathers were underrepresented, no check on reliability coding. Overrepresentation of middle-to-high socioeconomic status participants; possible social desirability bias.
Ivarsson et al.	To describe the journey from symptom onset to diagnosis of pulmonary arterial hypertension and chronic thromboembolic pulmonary hypertension from the perspective of patients and their spouses.	Secondary analysis of existing qualitative data (interview transcripts) from two previous studies; analysed using qualitative content analysis.	Seventeen patients (13 women, four men, aged 28–73 years), 14 spouses (five women, nine men, aged 40–87 years); nine of the participating groups were couples including patients and spouses.	Long delays in diagnosis, often exceeding a year, due to misdiagnoses or dismissal of symptoms; frequent visits to primary and specialist care before referral to pulmonary hypertension centres; lack of trust in healthcare professionals, coupled with relief at finally finding a diagnosis.	Primary care physicians, specialists in various fields, pulmonary hypertension specialist teams.	Secondary analysis, recall of patients may have affected statements. Not all spouses took part. Some patients did not take part, but their spouse did.
Jain et al.	To explore patient-physician communication about HAE, including barriers and gaps, and how communication impacts disease management.	Observational, qualitative study; data collected through in-office dialogues, follow-up physician dictations, patient and physician telephone interviews, and netnographic analysis of public social media posts on Facebook, Instagram, Reddit, and Twitter about HAE and related treatment from patients and advocacy groups associated with HAE.	Fourteen physicians, 29 patients, four caregivers; mostly female patients (71%), predominantly white (83%), ages ranging from 18 to 79 years.	Diagnostic delays were significant, and misdiagnosis was common. Treatment challenges included limited access to emergency medications, high costs, and logistical barriers. The burden of educating healthcare providers and advocating for care was frequently placed on patients. HAE rarity made patients feel isolated, reporting long diagnostic odysseys, and feeling tethered to their medicines and treatments.	Primarily allergists (physicians), with emergency and primary care physicians occasionally involved.	Small sample size. Physicians selected patients to include. Study was conducted before availability of subcutaneously administered long-term prophylactic treatments.
Johansen et al.	To explore support needs of parents of children (when their child was aged 0–5 years) with Usher syndrome (Type 1), particularly across social, informational, practical, and emotional domains.	Interviews with parents recruited via personal and professional networks and via an electronic newsletter sent via a support group managed by one of the researchers. Parents (six mothers) were included if their child was between nine and 25 years old. Data analysed using modified reflexive thematic analysis with member checking.	Six mothers of children with Usher syndrome Type 1, aged 46–59 years. Children’s current ages ranged between nine and 22 years; Usher syndrome diagnosis occurred at a minimum age of five years, and a maximum of 15 years old.	Parents reported extensive challenges, including limited professional knowledge of Usher syndrome, delays in diagnosis, and a lack of coordinated care. Many healthcare professionals were unfamiliar with condition-specific management strategies, requiring parents to educate clinicians and coordinate care. Financial burdens, limited access to respite, and lack of peer support groups further exacerbated difficulties.	Audiologists, ophthalmologists, physiotherapists, occupational therapists, geneticists, and allied healthcare professionals.	Small, homogeneous sample from a single support group. Findings may not generalise to fathers, siblings, or caregivers outside Australia.
Johansen et al.	To characterise the diagnostic trajectories of patients with autoimmune rheumatologic disease (such as rheumatoid arthritis, systemic sclerosis, and myositis) associated interstitial lung disease (ARD-ILD) and identify barriers to timely diagnosis based on patient and healthcare professional experiences.	Qualitative design; semi-structured interviews with 16 patients and 12 healthcare professionals (videoconferencing or telephone interviews). Patient recruitment occurred via pulmonologist.	Sixteen patients from three tertiary interstitial lung disease centres in Denmark. 12 females; four males. Aged 41–90 years. Twelve healthcare professionals, including rheumatologists, pulmonologists, and interstitial lung disease nurses.	Diagnostic trajectories for patients with ARD-ILD were characterised by delays due to limited awareness of association between autoimmune rheumatologic diseases and respiratory symptoms among general practitioners and other non-specialists. Early delays determined disease course and duration. Inconsistent disease terminology complicated diagnosis, as did fragmented healthcare provider communication. Patients reported repeated misdiagnoses, such as being treated for unrelated conditions like asthma/ pneumonia, and a lack of timely pulmonology or rheumatology referral. Delays often led to increased uncertainty, anxiety, and symptom worsening before diagnosis. Some diagnostic trajectories were prolonged by patients’ inability to recognise links between symptoms and underlying autoimmune rheumatologic disease. Patients noted frustration with long waiting times and the need to advocate strongly for specialist attention. Healthcare professionals confirmed systemic barriers, including inadequate referral systems and limited interdisciplinary collaboration.	Pulmonologists, interstitial lung disease specialists, interstitial lung disease nurses, rheumatologists.	None stated.
John Cherian et al.	To explore how families manage the complex medical needs of children with MECP2 duplication syndrome (MDS), particularly epilepsy, respiratory, and gastrointestinal issues, and the impact on parents.	Qualitative descriptive study; thematic analysis of semi-structured interviews (conducted via videoconferencing). Recruitment occurred via the international MECP2 Duplication Database.	International sample of 20 families, comprising twenty mothers and two fathers. Children with MDS included 17 males, most aged 11–20; most children were non-verbal, and 80% were unable to walk independently. Most lived in the United States, and others in Australia, Canada and Europe.	Families described significant challenges accessing healthcare for children with MDS, particularly when managing multiple complex conditions such as seizures, recurrent respiratory infections, and gastrointestinal issues. Many families reported a lack of professionals with adequate syndrome-specific knowledge, leading to inconsistent care and delays in receiving appropriate interventions. The responsibility for managing care often fell on parents, who coordinated between various specialists, navigated insurance or financial constraints, and addressed logistical challenges, such as sourcing medical supplies or travelling long distances for care. Parents expressed the emotional toll of advocating for their child’s needs and the frequent necessity to educate healthcare professionals about the syndrome. While multidisciplinary care teams were often involved, the quality of coordination among specialists varied significantly. Families also noted the importance of social networks, such as online support groups, in filling knowledge and resource gaps left by the healthcare system.	Study was about home management of condition by parents.	Limited geographical diversity and potential sample bias from recruiting through the MECP2 Duplication Database. Largely included participants receiving government support.
Johnston-Devin et al.	To better understand the phenomenon of chronic pain from lived experience perspective and how these influence care navigation and treatment.	Heuristic hermeneutic phenomenology, incorporating 17 semi-structured interviews (convenience sample from online forum), blogs, and a book of patient stories. Healthcare professional interviews were conducted to provide additional insights, recruited via a conference.	Seventeen participants aged 22–65 (mean age 44 years), including 14 women and three men, from various countries. Participants had symptoms ranging from four months to 18 years. Four healthcare professionals recruited. Overlapping sample see Johnston-Devin et al. next.	Participants reported delayed diagnosis, inadequate clinician knowledge, fragmented care, and disbelief. They highlighted barriers to accessing specialist care and described distrust in healthcare interactions, they reported battling for care and with their condition, and at times losing. Participants described having no effective treatment, and financial costs. Family and online communities played a significant role in providing support.	Pain specialists, general practitioners, physiotherapists, occupational therapists, and psychologists.	Limited sample diversity (Anglo-Saxon participants from high-income countries); did not include participants from closed online groups, potentially excluding different perspectives.
Johnston-Devin et al.	To explore the knowledge patients with complex regional pain syndrome (CRPS) believe healthcare professionals should have to provide effective care.	A heuristic, hermeneutic phenomenological study involving semi-structured interviews based around the interview question “what do you think health professionals should know about your condition to treat you more effectively?”.	Seventeen participants (14 women, three men; ages 22–65; mean = 44 years). Participants had experienced complex regional pain syndrome symptoms for four months to 18 years, with an average time to formal diagnosis of 2.65 years. Overlapping sample, see Johnston-Devin et al. above.	Participants reported poor understanding of CRPS among healthcare professionals, disbelief of symptoms, delayed diagnoses, and a lack of appropriate care. Many experienced distrust due to uninvited physical touch and ineffective communication.	Pain specialists, surgeons, physiotherapists, nurses, general practitioners, and multidisciplinary teams.	Small sample size, lack of linguistic diversity among participants, and limited geographical diversity in recruitment. Further studies are needed to address experiences of non-English speakers and others with chronic pain conditions.
Kerr et al.	To explore advice for families and clinicians regarding managing care for patients with complex vascular malformations, focusing on care challenges, quality of life, and patient-centred communication.	Semi-structured interviews with 21 adult patients and 24 parents of children with complex vascular malformations. Data were analysed using thematic analysis. Individuals were recruited via two support groups.	Twenty-one adult patients (aged 18–39) with vascular malformations and 24 parents of children with vascular malformations (aged < 18). Most were women. Sample partially overlaps with Sisk et al. (2022).	Participants reported challenges in accessing care due to limited clinician knowledge, poor care coordination, and fragmented healthcare systems. They highlighted unmet support and communication needs, and the importance of advocacy and support networks.	Multidisciplinary teams including primary care physicians, specialists in vascular malformations, and mental health professionals were identified as crucial for care coordination.	Homogeneous sample, mostly with severe disease. Limited diversity in the participant sample and focus on patients already engaged in support groups. Results may not generalise to those without diagnoses or those not benefiting from support groups.
Kiani et al.	To explore the qualitative effects of Iran’s economic sanctions on the perceptions/ experiences of individuals in Iran with rare and chronic illnesses.	Semi-structured interviews, focusing on patients’ lived experiences during the sanctions era. Patients were identified from Iranian Health Insurance Organization. Data were analysed through inductive content analysis.	A convenience sample of 31 patients (18 females, 13 males; aged 20 - 68 years) diagnosed with one chronic and/ or rare disease across four provinces, of differing economic levels.	Participants reported direct and indirect consequences of sanctions on their health conditions and life experiences. Those suffering from chronic and RDs were profoundly affected, grappling with the dual challenges of managing their illnesses and enduring economic hardships and lack of access to medicines. The findings illuminated how reduced affordability and economic adversity undermined an individual’s quality of life and coping mechanisms.	Not specified.	Convenience sampling strategy with limited participant numbers.
Kleinendorst et al.	To explore the impact of 16p11.2 deletion syndrome on family involved in daily care of children with this syndrome, and the availability of information to guide care and support.	Patients, parents, and other family members in a Dutch 16p11.2 deletion syndrome Facebook group were invited to participate in an information event. Based on this event, parents and other family members were invited to a focus group. Three focus group discussions were held. Focus groups were thematically analysed.	Twenty-three Dutch (grand)parents. All children had 550–600 kb 16p11.2 deletion, as did four participating parents.	Families experienced diagnostic odysseys lasting several years and involving many different physicians; participants expressed relief at having a diagnosis, noted their expert position, but also that this diagnosis did not always facilitate access to care and that having a more common condition would be easier to manage. Care was fragmented due to the syndrome’s variability and limited awareness among healthcare providers. Access to social, educational, and financial support was described as inconsistent and participants noted issues with social life for their children and some described their child’s condition as a burden.	Geneticists, physicians, dietitians, speech therapists, and educators; participants reported that many professionals lacked knowledge of the syndrome.	Small sample size of interested participants.
Kline et al.	To explore social media, particularly online health communities as a context, to uncover barriers to accessing genomic medicine among a population of those living with Ehlers–Danlos syndrome (EDS)/ hypermobility spectrum disorders (HSD).	Social media listening methodology was applied across a targeted sample of patient and caregiver conversations to identify barriers to genomic medicine access. The researchers analysed unique social media posts from an online health community (Inspire) over two-years (2019–2021). Key barriers were identified and classified by examining the conversations through analysis applying the modified social-ecological model.	Two hundred and thirty-two members of the EDS and HSD community providing 1949 unique posts with 345 posts specifically discussing barriers to genomic medicine. Posts were authored by patients, caregivers, and healthcare providers.	Participants reported long wait times and excessive travelling to see knowledgeable geneticists, high out-of-pocket costs, dismissive healthcare providers and disagreement between healthcare providers, and limited availability of specialists. Some faced psychological dismissal, where symptoms were misattributed to anxiety. Others commented on issues with individual health literacy, and misinformation/ lack of information around genomic medicine. Similarly, participants reported issues with interpreting their genetic results.	Required interaction with myriad healthcare professionals, although study primarily focused on geneticists.	Social media bias.
Ladores et al.	To explore the lived experiences of African American persons with cystic fibrosis (CF) focusing on potential healthcare disparities.	Descriptive phenomenology involving semi-structured telephone interviews with 12 African American adults with CF. Recruitment occurred through CF centres, social media, and snowball sampling. Data analysed using Colaizzi’s phenomenological method of thematic analysis to identify key themes.	Twelve African American participants living with CF (six men and six women; ages 23–45 years). Half of participants were on CF modulator therapy, while the other half were ineligible for modulator therapy.	Participants reported diagnostic delays due to provider bias (e.g., misconception that CF primarily affects White individuals). Many experienced racism in healthcare, including dismissive attitudes and lack of culturally competent care (including educational material). Access to modulator therapies was also inequitable due to rare genetic mutations making participants ineligible for drug trials.	Participants noted a lack of Black healthcare providers and culturally tailored support.	Small sample size impacting generalisability.
Levedahl et al.	To explore everyday experiences of people diagnosed with systemic mastocytosis (SM; indolent or advanced systemic mastocytosis) focusing on symptom burden, adaptation strategies, and healthcare professional interactions.	Descriptive qualitative study, semi-structured in-person interviews; recruitment occurred at one of the two university hospitals in Sweden with a centre for mastocytosis. Data analysed with inductive content analysis.	Sixteen participants diagnosed with indolent SM (*n* = 9) or advanced SM (*n* = 7). Age range 31–83 years.	Participants described fear of healthcare provider knowledge, inconsistent treatment guidance, and seeking information from other sources. They reported needing to become involuntary experts on their own disease to access appropriate care, and a lack of information or delayed care leading to more complication risk, but also being unable to engage in discussions with medical professionals due to the language medical professionals used. Many faced difficulties obtaining necessary treatments due to provider hesitancy or lack of awareness about SM, and others reported lack of care coordination. Participants reported alternating between feelings of vulnerability and acceptance, with some wishing to take active roles in treatment planning. Trusting relationships with healthcare providers and others promoted wellness perspectives.	Primarily focused on physicians.	Small sample size with unbalanced gender breakdown; results may not capture the full spectrum of SM experiences, particularly for those with milder cases.
Lewis et al.	To explore young people’s (aged 11–19 years) understanding, experiences, and participation in decision-making regarding rare disease genome sequencing, and how this impacts engagement with genomic medicine.	Qualitative study using semi-structured interviews with young people who had undergone genomic sequencing through the 100,000 Genomes Project. Abductive coding and thematic analysis was applied to explore knowledge, motivations, and decision-making. Interviews lasted 15 to 49 minutes; 25 were conducted over the telephone, two were conducted in person.	Twenty-seven young people (aged 11–18 years, mean 14 years; 19 female; 25 were probands and two unaffected siblings). Participants had skeletal (*n* = 8), renal (*n* = 4), dermatological (*n* = 3), and autoimmune (*n* = 2) conditions. Fourteen probands lacked a diagnosis, 11 had no known genetic aetiology, but had a working diagnosis.	Participants, while understanding wider basic genetic concepts, had varied levels of understanding of genetic testing. Participants valued the possibility of receiving a diagnosis but were concerned about uncertainty in the results. Some believed testing might end prolonged uncertainty, while others emphasised practical benefits. One third perceived benefits of a psychological nature, still others saw this as a validation opportunity. Many were unconcerned about data privacy but were anxious about what results might reveal. Noted the capability to make informed decisions, and that involving young people may empower them in taking responsibility for other healthcare decisions.	Primarily focused on physicians.	Study conducted in a single United Kingdom genomic research, programme participants self-selected to participate likely affecting generalisability.
Li and Yacyshyn	To investigate the perspectives and experiences of people affected by Behçet disease (BD) by examining the content shared and discussed on a subforum of Reddit to understand critical patient concerns and unmet needs.	Qualitative study using grounded theory; analysed 196 Reddit threads posted between March 9, 2021, and March 12, 2022 to identify themes and subthemes; parameters such as comments, net upvotes, and post content were extracted.	Members of the r/Behcets subreddit (approximately 1,100 members as of March 2022), predominantly patients (87%), with a mix of diagnosed (22%), suspected (23%), and implied (48%) BD and the rest having an unknown diagnosis (7%). Posters varied widely in age, with a mean age of 25 years when disclosed.	Challenges in diagnosis (lengthy diagnostic odysseys, misdiagnoses), often seeing multiple specialists without resolution; negative and dismissive experiences with healthcare providers; reliance on non-rheumatologists (e.g., dentists, gynaecologists) for initial suspicion of BD. The online community provided significant support and validation, which were often lacking in formal healthcare settings. In turn, the authors identified a key theme around the variety of symptoms thread participants discussed.	Rheumatologists (primary), dentists, dermatologists, gynaecologists, and general practitioners were noted as involved, though often with limited expertise in BD.	Participants’ diagnostic status could not be verified English language only. Reddit users may have been younger and more socioeconomically advantaged than the broader population. Posts on forum are moderated.
Lin et al.	To understand congenital ectopia lentis (CEL) patients’ care-seeking behaviours and factors affecting follow-up visits.	Grounded theory informed semi-structured interviews conducted with patients suffering from CEL and their parents.	Thirty-five participants (patients and parents) aged between four to 35 years, predominantly male. Participants were recruited from a single hospital and included sporadic and familial CEL cases.	Participants reported insufficient awareness of CEL. Participants indicated that they did not realise that CEL is life-threatening and interpreted the absence of discomfort as an absence of the disease. Physicians did not inform participants that CEL may be life-threatening. Many ophthalmologists were unfamiliar with CEL and were focused on patients’ ocular symptoms only, limiting management of systemic complications. Participants also felt shame due to the hereditary nature of CEL, potentially delaying treatment seeking. Many participants used search engines or social media to learn about CEL, information online was unreliable. Participants reported underestimating complications because of satisfactory treatment of ocular symptoms during childhood. Earning a living was often prioritised by parents and follow-ups were often postponed.	Ophthalmologists as point of entry.	Study conducted following sampling in one hospital. No consideration of other perspectives.
Litzkendorf et al.	To explore patients with rare diseases' and their relatives' use of rare disease information sources over time, identifying key challenges and changes in information-seeking behaviours.	Qualitative study using semi-structured interviews (55 patients, 13 relatives), after assessing interview transcripts, the team then undertook four focus groups, three with interview participants and one with patient representatives/ members of the chronic rare disease alliance as part of finding discussion and validation. Data were analysed using content analysis to explore themes related to information-seeking behaviours.	Fifty-five patients (aged 18–74 years) with a variety of rare diseases (e.g., genetic skin, eye, or kidney diseases, congenital metabolic diseases, skeletal dysplasia) and 13 close relatives participated in qualitative interviews.	Participants reported long diagnostic journeys and lack of adequate information at diagnosis. The Internet was the most commonly used initial source but was often overwhelming or insufficient. Patient organisations and close personal contacts made to support condition management (i.e., self-help), and specialised physicians gained importance over time. Self-help was seen as meeting demand for specific information not available online, but other participants noted that due to differences in illness trajectories, self-help/ engagement with patient organisations was seen as unhelpful. Participants criticised the information provided by general practitioners who did not appear to know a lot about their RD. Notably, participants highlighted that printed information, while high quality, was not up to date and sparsely used at later stages.	General practitioners, specialists, and specialised rare disease centres played roles in diagnosis and management.	Potential recall bias due to retrospective interviews and limited sample diversity in terms of disease progression stages. Sampling occurred at one rare disease centre, with a particular specialisation on rare skin diseases.
Livermore et al.	To explore the experiences of young people with juvenile dermatomyositis (JDM) coping with their disease, and understand the psychosocial effects and needs.	Interpretive phenomenological study that used qualitative interviews. Not specified if semi-structured, however, some interviews aided by creative methods, such as drawing; interviews occurred at home, in clinic, or on wards. The researcher then created poems out of the interview transcripts based on participant words.	Fifteen children and young people aged between eight and nineteen years with JDM.	Health experiences included frustration arising from difficulties getting a diagnosis, general practitioners not being aware of the condition, altered body images arising from treatment, and medical professionals were reportedly blasé about the impact of the treatment on the individual, feeling different and the invisibility and the potential long term implications of the disease.	General practitioners and specialists.	Small sample size: participants recruited from single site, limiting generalisability. Poetry as a method may not resonate with all audiences. Lack of member checking or involvement of participants in creating poetry.
Loizidou et al.	To investigate experiences of people with primary progressive aphasia (PPA) and their communication partners (families and friends) to understand how speech and language therapy can be improved to address their needs.	People with PPA and their communication partners (friends or family) were recruited for one of four focus groups (online video conferencing), via Rare Dementia Support PPA group newsletters. Participants were asked about communication difficulties, and how speech language therapy may address needs. Data were analysed using reflexive thematic analysis.	Seven people with PPA and 14 communication partners. Participants experienced symptoms for an average of 4.71 years (range: 1–10 years) and had been diagnosed for an average of 2.81 years (range: 0–7 years). Most communication partners (79%) were the partners of the people with PPA, and 57% of participants were female. Self-reported diagnoses included nearly half (41%) with non-fluent variant PPA. The largest group (47%) reported symptom onset and diagnosis within three to six years.	Participants faced challenges from symptom onset to diagnosis, often marked by delays and misdiagnoses. Communication partners took on roles to maintain communication, adapt strategies as symptoms progressed, and prepare for future needs, such as voice banking. Shifting responsibilities in caregiving strained relationships, requiring patience and coping mechanisms to manage behaviours and frustrations. Access to quality speech language therapists was inconsistent, with positive outcomes dependent on therapists’ familiarity with PPA. Participants emphasised the need for education on PPA, tailored speech language therapy strategies, and engaging people with PPA in meaningful activities to boost confidence. Challenges also stemmed from inconsistent service provision and funding constraints, making it difficult for some people to receive ongoing therapy. Unique needs and overlapping cognitive issues, like decision-making difficulties and fatigue, highlighted the importance of individualised approaches.	Speech language therapists, general practitioners, and neurologists.	Reliance on online meetings may have excluded individuals with limited digital access but benefited those with mobility challenges. The small, non-diverse sample size, limited to England and Wales, may not fully represent the socioeconomic, ethnic, and linguistic diversity of people with PPA and their communication partners. The predominance of communication partners without communication difficulties could have influenced discussions. Emotional impacts of PPA may have been underexplored due to the group setting. Potential biases from the senior author’s clinical background were mitigated through non-clinical facilitation and collaborative analysis.
Long et al.	To explore mitochondrial respiratory chain disorder (MRCD) management (healthcare, social support, management advice) for people living with a diagnosed MRCD, or the parents of children with a diagnosed MRCD.	This mixed methods exploratory study used surveys and focus groups to explore care experiences for individuals with genetically diagnosed MRCD and their parents, focusing on advice about nutrition, exercise, social care, and mental health. Recruitment, facilitated by the Mito Foundation, included virtual focus groups held via Zoom during COVID-19. Pre- and post-focus group surveys collected demographic data and feedback on recommendations. Transcribed focus group data were thematically analysed using NVivo, identifying “negotiation of care” as a central theme across five stakeholder groups, with visualisations created using UCINet. Survey data provided descriptive context for qualitative insights.	Twenty focus group participants (14 adults with MRCD and nine parents of children with MRCD, amongst these, three also had an MRCD diagnosis), with 70% having an MRCD, 45% being parents of a child with MRCD, and 15% fitting both categories. Most participants (75%) lived in metropolitan areas. Of 20 pre-focus group survey respondents, the average time engaging with the health system for MRCD care was 9.8 years, with 70% diagnosed over three years ago and 45% attending multidisciplinary MRCD services. Post-focus group survey feedback (*n* = 13) showed 85% found the new guidelines “extremely” or “very” useful, and 92% deemed them relevant to their situation.	Healthcare access and delivery for individuals with MRCD was characterised by constant negotiation across multiple stakeholders, including specialist and general health services, social care providers, and informal networks. Patients and parents often bore the burden of self-advocacy, navigating complex systems and advocating for appropriate care amidst uncertainty and limited evidence. Frustrations with social care systems, such as the National Disability Insurance Scheme, were prevalent, citing inadequate support and bias toward common conditions. Cross-cutting themes included high costs, trial-and-error management, trust issues, and challenges in transitioning from paediatric to adult care. Advice on supplements and exercise was common but often lacked evidence, while mental health support was rarely addressed, highlighting significant gaps in care provision. Transitioning from paediatric to adult services was challenging, often marked by a loss of holistic care and resources like specialist dietitians. Access to mental health support was minimal, often overshadowed by physical health priorities.	MRCD specialists, general practitioners, dietitians, exercise physiologists, psychologists, social workers. MRCD specialists and general health providers played a key role in care delivery, though interactions often required extensive negotiation. MRCD specialists generally supported management decisions despite uncertainties due to limited evidence, while generalists frequently lacked familiarity with MRCDs, requiring patients or parents to advocate for care. Trust was mixed, some providers fostered confidence through collaboration, while others withdrew support or disregarded patient expertise.	Online focus groups may have limited participation by those less digitally confident. People who participated may not be representative of the general population, the authors hypothesised the participants may be more proactive and informed.
Martinussen et al.	To explore parents’ experiences receiving diagnostic/ non-diagnostic genomic sequencing results for their child through the Victorian Undiagnosed Disease Program (UDP-Vic).	The study used a phenomenological framework to explore how parents/ guardians experience genomic sequencing through the UDP-Vic. Participants were purposively sampled from the UDP-Vic cohort and hospital patient records, with 57 invitations sent to eligible parents. Semi-structured interviews focused on the parents’ experiences with their child’s undiagnosed condition, participation in genomic investigations, and receiving results. Interviews lasted 25 to 105 minutes, and all participants chose to be interviewed individually. The interviews were audio-recorded, transcribed, and analysed using inductive thematic analysis. Data were coded using a codebook and analysed by multiple researchers.	Twenty-one parents (18 mothers, three fathers), including ten parents of children who received a diagnostic genomic test result and 11 parents of children with non-diagnostic results. Children’s ages ranged from three to 22 years, with ages at the first contact with genetics ranging from pregnancy to nine years, and the age at diagnosis ranging from three months to 18.5 years.	Parents faced long waits, averaging 13 years, for a genetic diagnosis, driven by a need for answers and hope that genomic sequencing might provide clarity (diagnostic odyssey). Parents of children with non-diagnostic results viewed the process as part of their child’s ongoing medical journey, while those with a diagnosis experienced a shift in understanding and how they approached family life. Emotions ranged from relief, especially for those ruling out severe conditions, to disappointment and continued uncertainty. Many parents sought connection with others in similar situations for emotional support, and most worked towards accepting their child’s condition, believing future advancements in genetics may provide further insights.	Healthcare professionals involved were not specified, but they facilitated access to the UDP-Vic genomic testing for children with undiagnosed conditions, helping parents navigate the diagnostic process. Parents were motivated by the hope that genomic sequencing could provide answers, but the impact of receiving results varied.	Exclusion of perspectives from those who declined genomic sequencing, which may be important for future research. As a retrospective study, the responses may have been influenced by participant recall bias.
Maxfield et al.	To explore lived experiences of adults with congenital disorders of the corpus callosum (DCCs) to inform development of adequate policy and service responses.	Phenomenological approach to understand the lived experiences of Australian adults with a DCC. Participants were purposively sampled from support groups and neurology departments, and were interviewed using semi-structured, face-to-face interviews. The study analysed the data using thematic analysis and interpretive phenomenological analysis, allowing for both descriptive and interpretive insights into participants’ experiences. Reflexivity was incorporated to address researcher subjectivity, especially given the lead researcher’s ‘insider’ status as a parent of an adult with DCC. The analysis involved multiple researchers and resulted in the identification of three key themes: reactions to the diagnosis, access to supports and life domains, and identifying as an adult.	Eight participants aged between 23-72 years, all received a DCC diagnosis in adulthood (one at age 16 years). The participants had undergone magnetic resonance imaging scans due to incidental clinical presentations such as seizures, injury, chronic headaches, anxiety, and autism. Educational backgrounds varied, with four participants completing undergraduate education, three having completed 10–11 years of mainstream schooling, and one attending 12 years in a special education setting.	Participants highlighted significant challenges in the diagnostic process and subsequent support. Participants were often unaware of their condition until it was discovered incidentally through magnetic resonance imaging scans. The delivery of the diagnosis was typically insensitive and unsatisfactory, with many participants experiencing shock, confusion, and frustration at the lack of clinician knowledge and guidance. Reactions from family and peers were mixed, with some offering support, while others used the diagnosis to humiliate or dismiss them. Access to healthcare and social supports, including mental health and disability services, was often hindered by insufficient understanding of DCC and a lack of appropriate resources. Many participants struggled with social inclusion, educational challenges, and employment barriers, with some facing bullying or discrimination. Despite these difficulties, some participants were able to persevere, achieving academic success and maintaining employment, though often with significant emotional and social costs.	Neurologists, general practitioners, and mental health professionals were generally mentioned, though none were reported as having substantial knowledge of DCCs. Many healthcare professionals had no prior experience with DCCs and provided little to no information. Lack of informed guidance left many participants feeling unsupported, as they struggled to access adequate care and support systems. The failure of healthcare professionals to provide appropriate follow-up or information on managing DCC-related challenges, such as mental health and disability services, exacerbated the sense of isolation and frustration among patients.	The study primarily focused on participants’ perceptions of barriers to education, employment, and access to services, and it may not fully capture the experiences of all adults with DCCs.
McLaughlin et al.	To explore experiences of people with severe haemophilia and healthcare professionals on pain management approaches, and views on exercise as a part of management.	Explorative study involving recruitment of people with haemophilia through social media and on posters in haemophilia centres. Healthcare professionals were recruited via professional haematology clinical interest groups of haematologists, nurses, physiotherapists, and psychology professionals. Recruited participants engaged in focus groups and semi-structured interviews, with two focus groups conducted in person with people with haemophilia before the COVID-19 pandemic and interviews carried out by phone or face-to-face (healthcare professionals) during the pandemic. Data were analysed using reflexive thematic analysis.	Fourteen people with haemophilia (11 attended two focus groups, three interviewed) and six healthcare professionals (two physiotherapists, one haemophilia nurse, two haematologists, and one psychology professional). People with haemophilia were aged 23 to 73 years.	People with haemophilia experienced healthcare delivery disconnection, particularly regarding pain management, but noted that feeling trust in care delivery was important to ongoing management. Barriers to care delivery from non-specialists were expected. Pain was often treated as a sign of bleeding, leading to reliance on clotting factor therapy, but this approach became less effective as bleeding episodes decreased. People with haemophilia reported frustration with healthcare consultations, feeling pain concerns were not fully addressed. Use of pain scales was often seen as unhelpful, and many avoided pain medications due to past experiences with addiction or concerns about long-term health. Exercise was viewed cautiously, with people with haemophilia reluctant to push their bodies due to fear of injury, but they were more likely to engage when supported by professionals who understand haemophilia. People with haemophilia prioritised maintaining function and reducing pain interference in daily life over complete pain relief.	Healthcare professionals involved in the care delivery included physiotherapists, haematologists, a haemophilia nurse, and a psychologist. The physiotherapists and haematologists primarily addressed pain management, bleeding, and exercise, while the nurse provided ongoing care and advice on treatment regimens. The psychologist helped with the emotional and psychological aspects of living with haemophilia, particularly the mental impact of chronic pain. Despite their specialised roles, healthcare professionals faced challenges in effectively managing pain, particularly chronic pain, due to a lack of coordinated multidisciplinary approaches and the unique needs of people with haemophilia.	Focus on people with severe haemophilia, which may not fully reflect experiences of those with moderate or mild haemophilia, and the potential influence of pain and bleeding concerns on pain management decisions in these groups.
Mills et al.	To explore experiences of patients with lymphoma in pregnancy (i.e., during pregnancy and up to 12 months postpartum). This included their needs, barriers to care delivery, and impact of heterogeneous healthcare systems.	The study involved women diagnosed between 2009 and 2020, who met specific inclusion criteria. Participants were recruited via telephone by their treating haematologist, and interviews were conducted by four physicians who had not previously met the participants in a clinical setting. The interviews, which lasted 15–20 minutes, were conducted by telephone and followed a standardised interview guide. Analysis of the transcribed data was performed employing Braun and Clarke’s method.	Thirty-two women with median age 32 years (range 24–42 years), diagnosed during pregnancy (63%) or postpartum (37%). Most participants (91%) received chemotherapy, with 69% starting treatment during pregnancy, and 28% received radiation therapy, all postpartum. The median time from diagnosis to interview was 48 months, and 78% had a public model of care. Only two participants were based in New Zealand.	Many women’s primary concerns were the welfare of their child and fear of dying, with some feeling their symptoms were dismissed due to pregnancy. Effective communication, particularly clear and empathetic discussions about treatment, was valued, though some experienced breakdowns between healthcare providers, leading to distress. Educational materials and patient advocacy were crucial, with most participants appreciating the resources available, though some felt unsupported by generic cancer groups. Psychosocial support, including financial assistance and access to counselling, was highly valued, though access to childcare and lactation support was limited. Oncofertility planning was often delayed, with many women suggesting more timely referrals and discussions about fertility preservation.	Healthcare professionals involved in the care of women diagnosed with lymphoma during pregnancy or postpartum included haematologists, obstetricians, maternal-foetal medicine specialists, paediatricians, psychologists, midwives, social workers, and radiation oncologists (some noted in discussion). Key aspects of their involvement included clear and empathetic communication, collaboration between specialists, and addressing psychosocial and oncofertility needs. Women valued the multidisciplinary approach to their treatment but reported challenges in communication, particularly between haematology and obstetrics. Psychological support, including social work, clinical psychology, and spiritual care, was also highly valued, alongside practical support such as financial and childcare assistance. The timely referral for oncofertility planning was identified as an area for improvement.	Potential impact of the median 48-month elapsed time between diagnosis and interview on participants’ recall of their experiences. Selection bias may have occurred, as some women declined participation due to psychological trauma, and those who participated may have had particularly positive or negative experiences. The exclusion of women with insufficient English proficiency limited the study’s external validity for non-English speaking populations. The study may have excluded patients from regional or remote areas due to the focus on tertiary referral hospitals, and those who had died before the interview were not included.
Ngoumou and Djouda Feudjio	To highlight how grassroots patient organisations can inform decision-making to address the needs of persons living with rare diseases and their families.	The study employed a mixed-methods research design combining a systematic scoping literature review and semi-structured interviews with parents of children with rare diseases who are members of patient organisations in Cameroon. The literature review aimed to assess the impact of patient organisations on research development, patient literacy, empowerment, and advocacy in Cameroon, resulting in 16 relevant articles after applying inclusion and exclusion criteria. The in depth in person semi-structured interviews focused on understanding the challenges faced by rare disease patients and their families in accessing healthcare and social support, and the impact of stigma. Data were analysed using content analysis to identify and group recurring themes.	Ten mothers of children with a rare disease who accessed patient organisations.	Mothers of children living with rare diseases faced significant challenges that severely affected their quality of life. These challenges included a lack of information and proper diagnosis, catastrophic medical expenses, stigma, and exclusion from social life. Many mothers struggled with the uncertainty of their child’s condition, as proper care and treatment options were often unavailable. The high costs of medications and medical care, compounded by limited resources and infrastructure, placed an overwhelming financial burden on families. The stigma attached to rare diseases often led to social isolation, as children with these conditions were frequently excluded from education and social opportunities due to physical abnormalities or misperceptions. As primary caregivers, mothers also endured emotional and physical exhaustion, exacerbated by societal discrimination and the lack of support services. In many cases, the role of patient organisations became crucial, providing vital support, information, and advocacy to empower families and improve their access to healthcare and social inclusion.	Not specified.	The study investigates the centrality of patient organisations but does not define what this is. There is no discussion within the study about any limitations.
Nguyen et al.	To understand the experiences of men living with breast cancer, specifically their experiences with the disease, supportive care needs, and experiences with MBC support groups.	Participants were recruited from a MBC patient support group and a university hospital, with eligibility based on a MBC diagnosis and written consent. Data collection involved open-ended semi-structured telephone interviews, guided by a developed interview framework, with topics adjusted according to patient narratives. Interviews were audiotaped, transcribed verbatim, and anonymised. Data analysis was performed using qualitative content analysis, with initial coding done by one researcher and refined through discussion with others.	Eighteen male patients, aged between 53 and 83 years, with a mean age of 68.5 years. The average time since diagnosis was 4.5 years (range: 2–8). Thirteen patients were retired, primarily due to age, while five were still working. The interviews lasted between 18 and 85 minutes, with an average duration of 41 minutes. Fourteen participants were married.	Men with MBC experienced significant challenges in healthcare access and delivery, often feeling marginalised due to the perception of breast cancer as a “women’s disease.” Many expressed frustration with being treated according to female-focused guidelines, such as hormonotherapy with medications primarily tested on women, and reported side effects like hot flashes and reduced libido. There was a lack of awareness and sensitivity among healthcare providers, with some professionals unsure about the responsibility for MBC care, and patients often faced difficulties finding appropriate physicians. Standard procedures, including administrative aspects like being addressed as “Mrs.,” also failed to accommodate men. Despite these challenges, emotional and social support from wives and MBC support groups played a crucial role in coping, although patients from the hospital were more sceptical about support groups. Patients expressed a need for male-specific psychosocial support, better information tailored to men, and more opportunities to share experiences with others affected by MBC.	Healthcare professionals, including general practitioners and gynaecologists, were unprepared to handle MBC in men, leading to misdiagnosis, inappropriate referrals, or a lack of proper care. Patients also indicated that standard procedures and guidelines often overlooked the needs of male patients, and some experienced stigma when treated in predominantly female settings. However, some professionals’ willingness to refer patients to specialists and offer medical care was appreciated.	Homogeneity of the sample, which may limit the generalisability of the findings to all men with breast cancer. As the participants were already receiving aftercare, those in earlier stages of diagnosis or with worse health might have different perspectives. The findings may not be applicable to other countries due to differences in healthcare systems. The potential impact of the interviewer’s sex on responses could not be determined.
Nguyen et al.	To investigate healthcare professionals' and parents’ perspectives, experiences and expectations with advanced therapies for rare neurological disorders (RND), and to identify treatment delivery and decision-making concerns, strategies and information/ support needs.	This study used a convergent parallel mixed methods design, collecting qualitative and quantitative data simultaneously. Participants included parents of children with RNDs and healthcare professionals, selected through purposive sampling to capture diverse experiences. Semi-structured Zoom interviews explored participants’ perspectives, with findings validated through member-checking, while online questionnaires assessed coping strategies, social supports, and emotional distress using tools like the DASS-21 and COPE Inventory. Qualitative data were thematically analysed using NVivo, and quantitative data were analysed with descriptive statistics in SPSS, with results integrated to align themes and quantitative findings.	Twenty parents and 20 professionals involved in the care of children with RNDs. Eighty percent of parents were mothers, 15% fathers, and 5% a female foster parent. The professionals were predominantly female (60%) and aged between 40 and 50 years (40% of clinicians). Most were located in New South Wales, with 45% of clinician participants being paediatric neurologists. The children all had genetic diagnoses, and 45% of parents had children who had accessed advanced therapies, with 67% of these therapies provided by the Australian healthcare system. The rest of the parents, although aware of their child’s genetic diagnosis, had no current therapies available for their children’s condition.	Stakeholders highlighted significant challenges regarding healthcare access and delivery, with a particular focus on the disconnect between the demand for advanced therapies and the limited knowledge and resources available to parents. While parents expressed interest in advanced therapies, many lacked understanding of clinical trial processes and had limited access to reliable information. The “therapeutic gap” and “therapeutic odyssey” were prominent, with parents facing substantial emotional distress and coping challenges. Information about advanced therapies was difficult to navigate, and there was a need for better, more accessible resources. Carers were highly motivated to pursue clinical trials, though concerns about informed consent and safety remained. The healthcare system’s inequities, particularly around accessing therapies within Australia and internationally, further complicated care delivery. There was a call for multidisciplinary, person-centred care approaches, integrating health and psychosocial support to help families navigate the RND treatment and decision-making.	Neurologists, geneticists, scientists, and allied health staff associated with neurology and clinical genetics clinics provided multidisciplinary care that integrated medical and psychosocial support, addressing both health and psychological needs of the child and family. These professionals guided decision-making around advanced therapies and clinical trials, often facing challenges in managing expectations and explaining risks and benefits to parents. They emphasised the importance of a biopsychosocial model to support parents through the emotional distress and complex treatment pathways, recognising the necessity of collaboration and ongoing care as part of a comprehensive therapeutic approach.	20% did not respond to the psychosocial surveys, which may introduce selection bias. The results differed from other studies on rare disease support, as many parents reported positive coping strategies and strong support networks, potentially skewing the findings. Findings may not fully capture the experiences of those outside specialised paediatric services. The study’s Australian focus limited generalisability, though the findings resonate with global challenges faced by families of PLWRDs, as highlighted by the United Nations.
Nixon et al.	To investigate primary haemophagocytic lymphohistiocytosis (pHLH) impact and treatment on patient and parent/ primary caregiver health and wellbeing.	Participants were identified through haemophagocytic lymphohistiocytosis patient advocacy groups in the United States and United Kingdom and were selected if they were parents/ caregivers of children under 18 or young adults aged 18–30 who had required bone marrow transplant for haemophagocytic lymphohistiocytosis in the last ten years. Semi-structured interviews were conducted in person or by phone. Interviews focused on the physical, emotional, and social impacts of haemophagocytic lymphohistiocytosis on patients and caregivers. Thematic analysis was conducted using both inductive and deductive coding approaches, and data saturation was reached when no new information emerged. Interviews were transcribed, and quotes were used to illustrate the themes identified.	Twenty-five participants: 21 parents/ primary caregivers of children with pHLH and four young adult survivors, primarily from the United States (81% of caregivers and all young adults). The caregivers had a mean age of 41.1 years (range 26–58), and most (81%) were female, with 76.2% being mothers. The majority (85.7%) had a college or university degree, and 52.4% were employed full-time. The 20 people represented by caregivers had a mean age of 12.75 years (range 5–31), with 85% alive at the time of the study. On average, symptoms were noticed at age 3.95, and diagnosis occurred at 6.1 years, with a mean diagnostic delay of 25.9 months. Over 90% of children received therapies such as corticosteroids, chemotherapy, immunotherapy, or haematopoietic stem cell transplant (HSCT). The young adult survivors (mean age 23.3, range 21–30) included three males and one female, all underwent stem cell transplantation.	Participants reported significant challenges in accessing and navigating healthcare for pHLH, marked by delayed diagnoses, prolonged symptom duration, and misdiagnoses (e.g., viral infections or leukaemia). The average time to diagnosis was 25.9 months for children and six years for young adults, with delays attributed to a lack of pHLH awareness among healthcare providers, repeated referrals, and difficulties finding stem cell donors. The physical and emotional toll of pHLH and its treatment, including isolation, disrupted education, and financial stress, heavily impacted both patients and caregivers. Caregivers often experienced mistrust of healthcare systems, exacerbated by perceived gaps in medical knowledge, which contributed to strained relationships and feelings of isolation. Long-term effects persisted post-treatment, with ongoing health monitoring, delayed development, and anxiety over recurrence, highlighting the enduring burden of pHLH.	Significant delays in diagnosis were often attributed to a lack of awareness and knowledge about pHLH among professionals, leading to frequent misdiagnoses, prolonged referrals, and inappropriate treatment approaches. Many caregivers expressed concerns about the medical profession’s ability to recognise and address the condition, which decreased trust in healthcare providers. Despite these challenges, some caregivers highlighted the importance of seeking care at specialised hospitals with experience in pHLH, emphasising the value of expertise in improving treatment outcomes. Post-treatment, ongoing outpatient monitoring by healthcare professionals was vital to managing long-term effects, such as compromised immunity, fertility issues, and psychological impacts, illustrating the enduring reliance on medical support in the journey of both patients and caregivers.	Challenges in recruiting patients with rare diseases, which may have limited the diversity of participants. Online recruitment methods introduced potential biases, favouring engaged individuals and those connected to advocacy or support groups. A survival bias was noted, as the study disproportionately represented patients with higher survival rates, potentially skewing the findings toward more positive experiences. There was also gender bias among caregivers, with interviews primarily involving mothers despite efforts to include both parents. The use of telephone and face-to-face interviews varied in effectiveness, with only a small number conducted in person despite the geographical spread of participants. Misdiagnosis was a concern, as distinguishing pHLH from secondary haemophagocytic lymphohistiocytosis at presentation is difficult, and both require similar treatments. The study lacked clinical data from medical professionals, limiting insights into the broader clinical impact of pHLH.
Palanisamy et al.	To identify the social networks of families within resource-constrained and marginalised Indigenous communities, and to explore how these networks support thalassaemia management.	This study employed a phenomenological qualitative research design. Using snowball sampling, the research was conducted at participants’ homes, with the first author establishing initial contact through a non-governmental organisation supporting affected families. The study involved in-depth interviews with parents whose children were diagnosed with β-thalassaemia major or intermedia. Data collection occurred between February and October 2017 in Tamil, with ‘saturation’ guiding the sample size. Interviews were transcribed, analysed, and coded, with meaningful themes emerging through open coding. A network map of social relations was created using NodeXL software, and triangulation was used to enhance the study’s credibility.	Forty-nine parents from 41 families. The parents were aged between 24 and 53 years, while their affected children were aged one to 17 years. Of the 46 children, 17 were girls and 29 were boys. Among the parents, 28 were female and 21 were male. Thirty-six parents had three or fewer children. Over half of the parents (*n* = 26) had no formal education, and 32 were employed in agriculture. In terms of family structure, 28 participants lived in nuclear families, and 13 lived in extended families.	Parents of children with thalassaemia relied on informal and formal social relations for healthcare access and support. Informal networks, including (extended) family, neighbours, and friends, played crucial roles in providing emotional, financial, and practical assistance, such as helping with transportation and blood donation. Due to limited resources within these networks, healthcare professionals, public health facilities, non-governmental organisations, and religious organisations were essential for securing regular disease treatment and information. Healthcare professionals in public and non-governmental organisation facilities were particularly valued for their compassion, providing medical treatment and emotional and informational support. Religious organisations and health education programmes helped parents navigate healthcare systems, offering financial support and advice on treatment options, including bone marrow transplants. Despite challenges, access to free blood transfusions and support from formal networks enabled families to manage their children’s condition more effectively.	Healthcare professionals were involved in managing thalassaemia in affected children, providing essential medical care, emotional support, and education. Physicians and nurses at public and non-governmental organisation health facilities offered routine check-ups and specialised care, often going beyond their duties to support families. These professionals also helped increase awareness about the disease, treatment options, and available resources, ensuring that families were well-informed and supported throughout the treatment process.	There is no discussion within the study about any limitations.
Pasquini et al.	To explore how parents of children with rare diseases experience health insurance engagement for their child and identify barriers to care and opportunities to address these barriers.	The study selected parents based on the similarities between the diseases, both of which involve genetic mutations and varying degrees of severity that affect motor functions and may require specialised care. Semi-structured interviews were conducted with eligible participants recruited through patient organisations. The study applied modified grounded theory methods, with iterative coding and constant comparative analysis to identify themes around healthcare accessibility, availability, and quality. Interviews were transcribed and analysed using NVivo 12, and coding reproducibility was checked with a research assistant. Data collection continued until saturation was reached, with no new themes emerging.	Fifteen parents participated in the study: four had one child diagnosed with metachromatic leukodystrophy, ten had one child living with SMA, and one parent had two children living with SMA. Among the participants, 14 were mothers and one was a father. The mean age of the parents was 39 years, while the mean age of the children was six years. The average time to diagnosis was 13.53 months. Six parents were from the southern regions, and five were from the Midwest region. Fourteen parents had at least a college degree, with 11 currently employed. All children had health insurance through at least one parent’s employer, and nine children were additionally covered by public insurance programmes such as Medicaid.	Healthcare access and delivery experiences for families navigating insurance were shaped by themes like involvement, support, obtaining insurance, interacting with insurance companies, and financial assistance. Families described the need for self-education and constant involvement in navigating insurance, with many facing confusion over coverage details. They often relied on disease-specific organisations, social support, and peer communities for guidance. Challenges included obtaining insurance, especially secondary coverage, and dealing with complex documentation and reauthorisations. Financial assistance, including copay programmes and personal fundraising, helped meet care needs. Despite frustrations with insurance systems, many families remained proactive, though the process was often time-consuming and emotionally taxing. Some families experienced fewer issues after successful treatment, but overall, access to specialised care and necessary equipment remained significant barriers.	Healthcare professionals assisted families with navigating complex insurance systems to ensure children with specialised medical needs received necessary care. They helped by submitting accurate claims, addressing coverage issues, and providing guidance on documentation. These professionals, including nurses and therapists, also offered direct support, such as therapy sessions and respite care for parents. They worked closely with insurance representatives to clarify coverage for treatments, equipment, and therapies, often coordinating with caseworkers to reduce financial burdens and ensure that care needs were met.	Small sample and potential lack of diversity may limit its findings. Self-selection bias could have skewed participation towards those more comfortable discussing insurance issues, and individuals not connected to patient organisations may be underrepresented. The sample was also biased towards females, higher-education individuals, and those from the Southern United States, which may not reflect the broader population. The absence of uninsured participants or those relying solely on public insurance limits applicability, and the study’s focus on equipment and therapy may overlook the needs of other rare disease populations.
Piercy and Nutting	To understand the experiences of parents of children who have a cerebral adrenoleukodystrophy diagnosis.	Participants were recruited through ALEX The Leukodystrophy Charity, ensuring variability across family characteristics and disease specifics. Data were collected via semi-structured interviews of 49 to 97 minutes, conducted remotely via Zoom or telephone, and transcribed verbatim. Thematic analysis was used to identify patterns and themes, with both researchers independently coding the data and refining themes through discussion. The final thematic structure was reviewed by an advisory group for validation.	Twelve parents from 11 families participated in the study. These families had a total of 25 children, 16 of whom had been diagnosed with adrenoleukodystrophy. Among the children with adrenoleukodystrophy, seven exhibited neurological symptoms, six experienced adrenal failure, three were diagnosed after their siblings were identified with the condition, and seven had received haematopoietic stem cell transplant (HSCT). The time since diagnosis varied, ranging from one to 20 years.	Parents of children diagnosed with childhood cerebral adrenoleukodystrophy described a challenging journey to treatment, with initial neurological symptoms often misattributed to other causes, such as the pandemic or a family situation. The eventual diagnosis was devastating, especially for those whose children were too far along for effective treatment. For those whose children were still candidates for treatment, the process was a “race against time”, as rapid deterioration meant that transplants had to be completed before the disease progressed too far. While some children underwent transplants and showed improvement, others faced further deterioration, leading to complex emotions about the treatment’s success. Parents also experienced prolonged periods of uncertainty while waiting for diagnoses or treatment opportunities, often facing the anxiety of not knowing whether their child would deteriorate. Despite the challenges, many parents emphasised maintaining normalcy in their family life and managing the care demands with a sense of resilience.	Physicians were involved in identifying early signs of cerebral damage through regular magnetic resonance imaging and making critical decisions about the timing of HSCT treatment. Social care providers helped manage increasing care needs, though delays in support were common due to the rapid deterioration of patients. Therapists were sometimes involved, but families preferred to maintain a sense of normality by limiting medical interventions. In cases where treatment was considered, healthcare teams supported families through the complex process of donor matching and the risks associated with HSCT. Despite treatment, healthcare professionals continued to monitor long-term outcomes, especially regarding potential risks like adrenomyeloneuropathy.	Restricted diversity of the sample as the study was limited to participants engaged with specific community-based organisations. Findings may not be fully representative of the broader adrenoleukodystrophy community. The majority of participants were mothers, which may have skewed the perspectives. The passage of time since the experiences described by parents varied, which may have influenced the data and the recollection of events.
Ragan et al.	To understand the education needs of families of children with intestinal failure and how online technology might effectively address these needs.	The study was conducted from November 2018 to February 2019 at a tertiary paediatric hospital in Western Canada. The study initially recruited 14 parents, and interviewed ten from the Children’s Intestinal Rehabilitation Program using purposeful sampling. Semi-structured interviews, lasting 45 to 80 minutes, were conducted primarily via phone. Data analysis followed Braun and Clarke’s six-phase framework, with themes refined through consultation with clinical experts and member checking. An audit trail ensured credibility, and findings were shared with other paediatric programmes to enhance validity and transferability.	Nine females and one male, with an average age of 35 years. Their children, evenly split between five males and five females, had an average age of four years and six months. On average, each child used two medical devices and received three treatments, including parenteral nutrition, enteral nutrition, modified diets, anticoagulant therapy, and oral medications. The duration of parenteral nutrition or active follow-up by the rehabilitation clinic programme varied: <6 months (*n* = 1), 13–24 months (*n* = 2), 3–5 years (*n* = 3), and >5 years (*n* = 4).	Parents of children with paediatric intestinal failure faced significant challenges in accessing and managing healthcare information, highlighting four key themes. First, they often lacked reliable, tailored informational resources and struggled with overwhelming medical terminology, cognitive load, and the distress of unknowns, leading them to rely on trusted but limited access to their medical teams. Second, hands-on, in-person learning during transition programmes was highly valued, though better mental health support and expanded training for staff were recommended. Third, practical, day-to-day information and connections with other families were prioritised over medical knowledge, with social media being a “double-edged sword” for support and misinformation. Finally, participants expressed a need for curated electronic resources, such as instructional videos and shared tracking templates, to enhance their understanding, reduce stress, and improve communication, though these should complement, not replace, personal interactions with their healthcare providers.	Healthcare professionals, particularly nurses and physicians, provided essential support, education, and communication to parents. Nurses led hands-on training for home care, such as paediatric home parenteral nutrition management, helping parents build confidence in managing their child’s condition at home. Rooming-in days allowed parents to take full responsibility for care before discharge. Parents also suggested more staff training and digital resources, like instructional videos and tracking platforms, to improve accessibility and ongoing support.	Participant variability in age, with some having been involved in the programme for over two years, potentially influencing their retrospective observations and recall. The sample may be biased toward families of children with more complex cases, which could differ from those with better prognoses. The study was skewed towards female perspectives and did not account for cultural or economic differences among parents. Responses were limited to a single centre, so generalisability to other programmes may be limited.
Respondek et al.	To understand clinical (healthcare) and emotional journeys of patients with progressive supranuclear palsy (PSP), caregivers, and healthcare professionals from early symptom onset to mortality, and to identify how needs could be met, and care delivery could be improved.	A literature review was conducted to map the clinical journey and identify barriers to optimal care, followed by interviews with six PSP experts to refine and validate the clinical pathway. Opinion leaders from seven countries reviewed findings for further validation. To investigate the emotional journey, research involved 112 participants (patients, caregivers, healthcare professionals, and patient organisation representatives) across the same countries. Methods included 60-minute interviews and seven-day smartphone diaries for patients and caregivers, capturing daily experiences and emotions through photos, videos, and surveys. Participants’ emotional responses were classified using a simplified version of Plutchik’s psychoevolutionary theory of emotion.	One hundred and twelve participants representing diverse perspectives on PSP. This included 21 patient-caregiver pairs, seven representatives from patient organisations, 42 key opinion leaders, and 21 nurses. Key opinion leaders were selected based on their roles as neurologists or movement disorder specialists involved in treatment decision-making, with 3–40 years of clinical practice experience, spending at least 50% of their time in direct patient care, and no affiliation with the pharmaceutical industry. Nurses were included if they had 3–40 years of professional experience and regularly cared for individuals living with PSP.	Healthcare access and delivery for patients with PSP was characterised by significant challenges, including delayed diagnoses, limited healthcare professional knowledge, and inadequate support systems. The diagnostic process often spanned 1.5–3 years due to the rarity of PSP and its symptom overlap with other conditions, causing frustration for patients and caregivers. Many patients felt that primary care physicians and neurologists lacked adequate understanding of the disease, providing insufficient information or empathy during diagnosis. While Japan offered better support with access to care managers and home visits, other regions relied on inconsistent multidisciplinary care. Caregivers faced escalating physical, emotional, and financial burdens as patients became increasingly dependent, with limited resources available to support their mental health.	Healthcare professionals’ contributions were often limited by gaps in knowledge, resources, and systemic support. Neurologists, particularly movement disorder specialists, were central to diagnosing PSP, although delays were common due to disease rarity and symptom overlap with other conditions. In some regions, multidisciplinary care existed informally, relying on ad hoc collaboration between healthcare providers rather than structured systems. Physiotherapy and speech therapy were increasingly used to address PSP symptoms, though access to these services was inconsistent. Nurses, while identified as underutilised, have the potential to enhance education and awareness about the disease.	Inability to account for geographical differences in care pathways, patient evaluation, and treatment approaches in countries not included in the research. It does not differentiate between emotional changes caused by PSP symptoms and those arising from the diagnosis and disease progression.
Rintell et al.	To address the lack of published data on patient and family experiences with transthyretin amyloidosis (ATTR) and explore diagnostic processes, symptoms, and quality of life impacts.	Focus groups were conducted to explore the experiences of patients and family members affected by ATTR. Collaborating with patient organisations in the United States and Argentina, researchers convened a transthyretin amyloid cardiomyopathy (ATTR-CM) group in San Francisco and a transthyretin amyloid polyneuropathy (ATTR-PN) group in Buenos Aires. Discussions, facilitated by an experienced psychologist using semi-structured guides, focused on diagnosis, symptoms, and quality of life impacts. Transcripts were analysed using a structured coding process to identify patterns and themes, ensuring participant anonymity and leveraging simultaneous translation for the Spanish-language group.	Two focus groups recruited through patient organisations: one in San Francisco, United States, for ATTR-CM and another in Buenos Aires, Argentina, for ATTR-PN. The San Francisco group consisted of seven patients (four with wild-type ATTR-CM and three with hereditary ATTR-CM) and three family members, while the Buenos Aires group included ten patients and five family members, several of whom had immediate or extended family members with ATTR-PN.	Participants in ATTR focus groups described the challenging healthcare experiences associated with accessing and receiving a diagnosis and treatment. The journey, often referred to as a “diagnostic odyssey,” involved enduring lengthy delays, misdiagnoses, and inappropriate treatments due to a lack of awareness about ATTR among healthcare providers. Patients with ATTR-CM and ATTR-PN reported a range of debilitating symptoms affecting multiple organ systems, which significantly impaired their quality of life and ability to engage in daily activities. Both groups highlighted the emotional toll of the disease, particularly on family relationships, with family members playing a vital role in managing care and coping with the disease’s impact. Despite the challenges, some participants took active roles in seeking information and specialists, which occasionally led to accurate diagnoses and better care outcomes. The profound emotional and physical effects of ATTR underscored the importance of improving diagnostic processes and healthcare support for these patients.	Primary care physicians, specialists, and even non-medical healthcare workers could miss the diagnosis due to the rarity of the condition and its nonspecific symptoms, causing delayed treatment. Patients and families often took an active role, using resources like online research to find specialists when physicians lacked knowledge.	Small sample size of ATTR patients who were primarily known to patient organisations, which may not be representative of all ATTR patients. The lack of participant age information further limits the diversity of the sample, potentially skewing results towards a more informed and disease-educated cohort. The study relied on self-reported data without independent validation of health claims or experiences, and some patients were more vocal than others, meaning not all experiences were equally represented.
Rodríguez-Laguna et al.	To describe patient and caregiver journeys during PIK3CA-related overgrowth spectrum (PROS) diagnosis and treatment and raise awareness of these RDs.	The research used qualitative and quantitative methods. A literature review was conducted with PubMed and data from advocacy and institutional websites. Ethnographic research involved interviews with ten adult patients, 14 caregivers, and healthcare providers from the United States, Germany, and France to explore diagnosis, treatment, and challenges. Quantitative data were collected on diagnosis rates, treatments, and referral patterns. Social media listening was also employed to track trends. Findings were validated through feedback from advisory boards and PROS advocacy groups, mapping patient journeys and key themes related to disease experience and management.	Twenty-four patients, caregivers, and advocates, with data from 223 patients reviewed. A visual map of the PROS patient and family journey was created to highlight personal health and system challenges, as well as opportunities for improvement throughout patients’ lives. Separate maps were also developed for Klippel–Trénaunay syndrome (K-T), congenital lipomatous overgrowth, vascular malformations, epidermal nevi, scoliosis/skeletal and spinal anomalies (CLOVES syndrome), and megalencephaly-capillary malformation (M-CM).	The path to diagnosis for PROS conditions could be long and challenging due to their rarity and overlapping symptoms. Patients often saw multiple specialists, delaying diagnosis, and many physicians were unfamiliar with PROS, leading to misdiagnoses and unnecessary testing. Genetic testing, though key for diagnosis and treatment, faced barriers like cost, accessibility, and confusion around mosaic mutations. Patients and families also faced substantial challenges with treatment, including limited access to specialised centres and therapies, the need for ongoing surgeries, and managing emotional and financial pressures. Support from allied health services was crucial, but often fragmented. The transition from paediatric to adult care was difficult, and many families experienced social and emotional strain. Advocacy groups played a vital role, but patients often struggled to connect with others due to the diverse nature of PROS conditions.	Patients were often first seen by family paediatricians, general practitioners, or dermatologists, with referrals to specialised centres when needed. In Germany, patients were promptly referred to geneticists at rare disease centres, while in the United States, a majority of patients received a diagnosis at birth or in utero, though misdiagnosis was common. A multidisciplinary care team, often led by paediatricians, haematologists, oncologists, or surgeons at specialised vascular anomaly centres, was essential for managing complex cases. Caregivers may also engage with physical, occupational, feeding, and speech therapists, although these services were often not well coordinated with the primary care team. During transitions to adult care, patients may face challenges in finding specialists experienced in treating PROS in adulthood.	The research was limited to healthcare professionals in the United States, Germany, and France, reflecting practices in these countries only. The ethnographic research focused on United States-based patients with specific conditions (K-T syndrome, CLOVES syndrome, and M-CM), which may not fully represent the broader PROS patient population. The findings may not capture the full diversity of patient experiences or be universally applicable to all PROS disorders.
Rosenfeld et al.	To understand patient and caregiver perspectives on Undiagnosed Diseases Network engagement, whether this resulted in a diagnosis or not.	Participants were recruited via email, newsletters, and social media to represent diverse characteristics. Discussions, guided by a structured framework, focused on the impact of Undiagnosed Diseases Network evaluations on medical care, health, interactions, and outlook. Data were collected through recorded sessions, transcriptions, and field notes, with thematic analysis conducted using content analysis and a grounded theory approach. Themes were validated through iterative review and consensus among coders and the research team.	Thirty focus group participants, divided into adult undiagnosed (*n* = 6), adult diagnosed (*n* = 7), paediatric undiagnosed (*n* = 8), and paediatric diagnosed (*n* = 9) groups. Most participants identified as female (66.7%) and White (56.7%), with 16.7% identifying as Asian, and 13.3% as Black or African American. Household income varied, with 33.3% reporting incomes under $20,000, 20.0% over $80,000, and 33.3% preferring not to answer.	Participants in the study highlighted diverse experiences with healthcare access and delivery through the Undiagnosed Diseases Network. Adults with undiagnosed conditions valued the Undiagnosed Diseases Network evaluation for validating their medical concerns, improving provider interactions, and facilitating referrals. Those with diagnosed conditions emphasised the need to be their own health advocates due to system gaps, and described the healthcare system as not being set up for rare disease. Paediatric participants appreciated the Undiagnosed Diseases Network’s role in ruling out syndromes, managing care, and fostering empathy among their communities. Across focus groups, both diagnosed and undiagnosed participants expressed gratitude for the comprehensive evaluations and reported a positive future outlook, finding new strategies for care management and a sense of validation in their diagnostic journeys.	Healthcare professionals provided comprehensive evaluations that considered patients’ varied health concerns. Their involvement was particularly appreciated for ruling out potential diagnoses, validating patients’ and caregivers’ experiences, and offering referrals to relevant providers. Participants valued the willingness of Undiagnosed Diseases Network teams to listen holistically, which contrasted with fragmented care experiences in other healthcare settings. This comprehensive approach helped guide care prioritisation and management strategies while fostering trust and hope among patients and families.	Exclusion of non-English speakers due to time and funding constraints, which limited the diversity of perspectives. The study relied on a 60-minute time limit for focus groups, which may have constrained in-depth discussion. The virtual format may have led to the presence of other individuals, potentially influencing responses. The inclusion of a now-adult participant who was evaluated as a minor in the Undiagnosed Diseases Network could have affected the relevance of her contributions, potentially limiting the generalisability of the findings.
Simpson et al.	To explore how rare disease patients, and parents/ spouses/ partners of patients are impacted by how their care is or is not coordinated and explore the factors which might influence effective care coordination.	Participants were sampled from charity networks to capture diverse perspectives on care coordination, including diagnosed and undiagnosed conditions, and varied coordination approaches. Fifteen semi-structured interviews were conducted via phone or Skype between October 2018 and January 2019. Interview questions explored care organisation, preferences, and associated costs and benefits. Transcripts were recorded, anonymised, and analysed using a systematic coding framework in NVivo 12. Themes were developed through iterative categorisation, with input from clinical and voluntary sector experts.	Fifteen participants: seven patients with rare diseases, four parents of diagnosed children, two parents of undiagnosed children, and two spouses/ partners of diagnosed adult patients. Participants ranged in age, with the majority (*n* = 12) aged 26–59 years. The majority of people receiving care (*n* = 6) were under 18 years. Participants resided across various United Kingdom regions, including East Midlands, Greater London, and Scotland. Five patient participants had a diagnosis and two were undiagnosed; six participants were caregivers of diagnosed patients and two of undiagnosed patients. Regarding care coordination, nine participants coordinated care themselves, while professionals coordinated care for six participants.	Patients with rare diseases often faced fragmented healthcare experiences due to uncoordinated care, which manifested in frequent and scattered appointments, ineffective communication among stakeholders, and reliance on patients and caregivers to manage their own care. These challenges led to delays in accessing timely and appropriate care, exacerbating physical health issues, financial burdens, and emotional stress. Patients reported significant time and effort spent on coordinating their care, including tracking medical records, facilitating communication, and navigating complex healthcare systems. Uncoordinated care also affected broader family dynamics and disrupted daily life, work, and school activities.	Healthcare professionals’ effectiveness in patient care was often limited by uncoordinated systems. Some professionals in specialist centres served as key points of contact, managing appointment schedules and facilitating information sharing between stakeholders, including local and specialist services. However, ineffective communication across care teams was a recurring issue, with delayed information sharing and limited collaboration hindering timely decision-making and access to care. In many cases, patients and caregivers took on the burden of facilitating communication and ensuring professionals had accurate and up-to-date information.	Lack of a representative sample, and the study may not capture all factors influencing care coordination, such as institutional barriers like resource availability, structural limitations in National Health Service information systems, or cultural resistance to change.
Sisk et al.	To explore parental insights into the barriers and enablers to accessing specialist care for their children with vascular malformations.	Parents were recruited from two patient support groups, and semi-structured interviews of 39–73 minutes were conducted via phone or video from June to October 2021. The interview guide, developed with input from parent advocates, focused on disease characteristics, care barriers, and communication experiences. Data were analysed using thematic analysis, and descriptive statistics were used for demographic data. The study aimed for thematic saturation with at least 20 participants, and coding was verified for agreement.	Twenty-four parents, the majority were White (92%), female (87%), and held college or professional degrees (62%). Parents’ ages ranged from 21 to 54 years, with a mean of 41 years. They cared for children aged from infancy to 16 years (median age: 11 years), with an equal representation of male and female children. All children had health insurance, and parents reported high disease severity and interference in their child’s life. Participants came from 14 states across various United States regions. Sample overlaps with Kerr et al. (2023).	Families navigating care for children with complex vascular malformations and overgrowth disorders faced challenges accessing and maintaining effective care. Parents highlighted the importance of clinician knowledge, commitment, and advocacy but often encountered unprepared/ dismissive providers, necessitating their own relentless advocacy to coordinate care and seek expert input. Accessing multidisciplinary teams was crucial but frequently required travel, prolonged wait times, and navigating complex healthcare systems, including insurance challenges and financial burdens. Social networks and patient advocacy groups provided valuable guidance, reassurance, and financial support, though misinformation and emotional distress were risks. Scientific gaps, limited information, and lack of tailored research added to parental difficulties, leaving families reliant on fragmented, outdated resources. Many parents credited privilege/ luck for achieving better care, while access to accurate, comprehensive information emerged as cross-cutting needs.	Healthcare professionals provided referrals, advocated for the child, and coordinated care across specialists. However, many parents reported that non-specialist physicians lacked knowledge, requiring parents to take an active role in educating clinicians. The involvement of healthcare professionals, including facilitating access to multidisciplinary teams, was essential for accurate diagnosis and treatment, but gaps in knowledge and care coordination often created significant challenges for families.	Sample predominantly consisting of White, female, well-educated, and high-income participants, which may not fully represent the diverse challenges faced by families with varying racial, socioeconomic, or geographical backgrounds. The participants had already accessed patient support groups and received a diagnosis, potentially underrepresenting barriers to diagnosis and ongoing care. The study may have been influenced by recall and conformity biases and lacked access to clinical information that could provide deeper insights. The perspectives of patients themselves were not considered, which could have highlighted unique challenges as they transition to self-management.
Soto Jansson et al.	To gain a comprehensive understanding of the experiences and needs of caregivers of children with Dravet syndrome (DS) regarding diagnosis, treatment, and supports for neurodevelopmental problems in a population-based sample via semi-structured interviews.	Semi-structured interviews with caregivers of children diagnosed with DS in Sweden. Participants were recruited through hospitals, networks, and associations, with interviews conducted in-person or via telephone between October 2018 and April 2020. Data were analysed using inductive reflexive thematic analysis, emphasising patterns of shared meaning without predefined coding frameworks. Two researchers conducted iterative coding and theme identification, resolving discrepancies through consensus with the research team. Interviews were transcribed verbatim, and themes were developed using a semantic approach, prioritising explicit meanings within the caregivers’ responses.	Caregivers of 36 children with DS in Sweden, predominantly biological mothers (*n* = 30) and fathers (*n* = 13). The children had a median age of 8.09 years at assessment and were diagnosed with DS at a median age of 2.20 years, with most being male (56%) and 97% carrying a known SCN1A variant. Epilepsy-related milestones included a median age of 0.42 years for the first seizure, 0.8 years for epilepsy diagnosis, and 0.85 years for the first episode of status epilepticus. Children used a median of three current anti-seizure medications and had tried six overall, starting at a median age of 0.83 years. Learning disability was present in 92%, with mild levels in 33%, severe in 31%, and moderate in 28%. Caregivers were typically in their late 30s (mothers) to early 40s (fathers), with mothers working fewer hours (13.33 hours/ week) than fathers (36.65 hours/ week).	Caregivers reported mixed healthcare access and delivery experiences. Diagnostic delays often occurred due to initial misinterpretations of symptoms, or requiring caregiver persistence to secure genetic testing. Communication of the diagnosis was described as overwhelming, with some caregivers finding medical professionals knowledgeable, while others relied on the Internet/ patient organisations for information. Support following diagnosis was inconsistent, with many emphasising the value of connecting with DS experts/ organisations. Epilepsy treatment experiences varied, with caregivers expressing frustration over medication side effects, uncertainty about efficacy, and a heavy responsibility in managing treatment decisions. Emergency care was anxiety-inducing, with caregivers guiding medical teams due to insufficient provider expertise. Developmental and behavioural support access was hindered by system fragmentation, geographical inequities, and difficulties securing diagnoses critical for unlocking services.	Healthcare professionals were involved in diagnosing epilepsy and DS, although delays and a lack of proactive genetic testing were noted. During treatment, some professionals demonstrated strong knowledge of DS, developing effective management plans and listening to caregivers, while others relied heavily on caregivers for treatment decisions, particularly in emergencies. In emergency care, professionals sometimes sought advice from caregivers, highlighting expertise gaps. After diagnosis, access to specialised DS experts was valuable for some families, but caregivers felt the follow-up support from healthcare providers was limited. Integration between hospital-based epilepsy care and disability services was often lacking, contributing to fragmented care delivery.	Not all caregivers participated in interviews. Interviews conducted both in-person and via telephone may have influenced the responses’ content and length, and using two interviewers could have affected reliability. The study’s findings are specific to Sweden, limiting generalisability to other countries. Data were collected before updated diagnosis and management guidelines, so some experiences may not reflect current practices. Limited information on participants’ socioeconomic status restricts understanding of its impact on caregiver experiences.
Stamou et al.	To explore and gather in-depth insights into services perceived as helpful by individuals with young-onset dementia (YOD) and their family caregivers and to enhance the provision of effective YOD services by employing an adapted appreciative inquiry approach to collect and analyse examples of supportive service experiences shared by these individuals and their caregivers.	This study used a qualitative design based on the discovery and dream phases of appreciative inquiry to explore the experiences of individuals with YOD and their family caregivers. Data were collected via a nationwide survey and semi-structured interviews, capturing socio-demographics, service use, and examples of helpful support. Themes from survey responses were analysed inductively, while interviews provided deeper insights into emerging themes. Recruitment included diverse diagnostic pathways through National Health Service sites, research registries, and charities. Quality assurance involved reflexivity, stakeholder feedback, and iterative team discussions to ensure credibility and confirmability.	Twenty-six participants, comprising ten individuals with YOD and 16 caregivers. Among those with YOD, the mean age at diagnosis was 57.4 years, and the mean age at participation was 60.6 years. Most (70%) were female, with Alzheimer’s being the most common diagnosis (40%). Other diagnoses, each accounting for 10%, included frontotemporal dementia, vascular dementia, mixed dementia, and posterior cortical atrophy, while 20% were unsure of their diagnosis. Half had lived with the condition for 5–6 years. Caregivers had a mean age of 52.63 years at the time of the YOD diagnosis and 56.13 years at study participation, with 56.2% being female. Most caregivers (81.1%) were spouses, while 6.3% were daughters, sons, or partners. The majority (87.5%) lived with the person with YOD.	Participants highlighted the importance of integrated, consistent, and specialist services for YOD. Integrated care pathways and collaboration between services, such as memory clinics and general practitioners were highly valued for their tailored, coordinated approaches. Specialist YOD services provided emotional and practical support, offering consistency over time and fostering trust. Age-appropriate, holistic, and responsive care was emphasised, addressing both patient and family needs, with activities and support tailored to younger individuals. Accessibility, including local availability, affordability, and suitable scheduling, was crucial, particularly for working caregivers. Person-centred care was characterised by respect, compassion, attentive listening, and flexibility, with in-person support and user-friendly materials fostering meaningful engagement and collaboration with professionals.	Dementia nurses collaborated with diabetic nurses to ensure coordinated care for individuals with multiple health concerns. The involvement of designated professionals, such as a link worker, was highlighted in guiding patients and caregivers through tailored care pathways. Additionally, caregivers expressed appreciation for the emotional support provided by YOD specialist services, which improved their experience significantly.	Potential selection bias, efforts to recruit people from racially and ethnically minoritised groups were limited, with only a few responding. The use of briefing notes may have influenced participants’ recall, though most responses were specific and detailed. No participants under 50 were interviewed.
Verger et al.	To analyse the coordination between healthcare and education professionals involved in supporting children and adolescents with rare diseases.	Applying a social-critical paradigm, participants, including parents, healthcare professionals, and educators with relevant experience, were recruited through schools and a local rare diseases association. Data were collected via six semi-structured focus groups grouped by professional role or family background to encourage open discussion. Sessions were recorded, transcribed, and analysed using discourse analysis, with triangulation against prior research to ensure rigour and methodological consistency.	Fifty participants: eight parents of children with rare diseases (six women, two men), three nurses (women), five school physiotherapists (three women, two men), eight special education assistants (women), six primary school teachers (four women, two men), six primary school assistant teachers (five women, one man), three hearing and language teachers (women), two primary school educational psychologists (one man, one woman), secondary school teachers and two secondary school educational psychologists (women). As for parents, families with children with different rare physical and cognitive disorders attending primary (four) or secondary education (five) were selected. Participants’ ages ranged from 32 to 56 years old.	This study highlighted significant challenges and opportunities in the coordination and communication between schools and healthcare services for children with rare diseases. Coordination was often limited to health emergencies, with scarce ongoing collaboration and a lack of established protocols, particularly in larger secondary schools. Barriers included insufficient knowledge about rare diseases, limited interprofessional collaboration, and an over-reliance on families to bridge communication gaps, which could impose additional burdens on them.	Nurses provided health promotion talks and training on managing health crises, and physiotherapists performed interventions in schools to promote diversity and inclusion. They collaborated with educational staff to align healthcare support with school programming and ensure children’s safety and well-being. Healthcare providers from primary care centres and specialised care facilities were engaged in providing decentralised and continuous care, reserving hospital care for more specialised needs.	Potential influence of specific rare disease characteristics on participant experiences and the lack of heterogeneous groups combining families, educators, and healthcare professionals, which may have limited insights into coordination challenges.
Walton et al.	To develop and refine a care coordination taxonomy for individuals with RDs.	In stage one, 30 interviews with healthcare professionals, charity representatives, and commissioners, along with four focus groups with patients/ caregivers with rare or undiagnosed conditions, were conducted. Audio-recorded data were professionally transcribed, anonymised, and thematically analysed to identify coordination models and create the taxonomy. In stage two, two workshops were held to refine the taxonomy using pre-shared materials and structured discussions. Participants were recruited to ensure diverse representation, and the taxonomy was iteratively developed following a six-stage framework, emphasising flexibility and applicability across care contexts.	Seventy-seven participants, with data from 52 individuals (30 interviews and 22 focus group participants) used for the taxonomy development and input from 27 workshop attendees refining it. Interviewees included 15 healthcare professionals and five charity representatives, geographically distributed across England, with some holding national roles. Focus groups primarily involved patients with rare or ultra-rare disorders (*n* = 16), most aged 26–59 years, and 14 accessing specialised services, predominantly in London. Workshop participants included patients, caregivers, healthcare professionals, charity representatives, and commissioners, many aged 26–59 years and with RDs. A notable proportion coordinated care themselves, reflecting diverse perspectives in shaping the taxonomy.	Healthcare access and delivery for RDs can be organised through various models, ranging from centralised national care centres to hybrid systems that combine specialist hubs with local care, and purely local provision. Coordination relies on collaborative efforts across professionals, patients, and caregivers, with multidisciplinary teams and joint clinics playing a key role. Responsibilities include administrative support, clinical leads, and care coordinators, with patients and caregivers often taking on coordination tasks themselves. Appointments may be regular, on-demand, or a hybrid of both, and access to health records ranges from full to restricted, with options for patients and providers. Communication uses digital, written, and face-to-face methods, with COVID-19 driving increased adoption of virtual care delivery and coordination. However, challenges persist, including gaps in collaboration, accessibility, and information sharing.	Healthcare professionals are involved in multidisciplinary team meetings, one-stop clinics, and joint appointments, where specialists work together or in conjunction with local providers to ensure comprehensive care. Professionals also serve as coordinators, clinical leads, or collaborators, managing responsibilities such as arranging appointments, implementing care plans, and overseeing service quality. They provide administrative and clinical support, such as maintaining care pathways, facilitating communication, and ensuring care continuity. Effective collaboration among professionals is vital, but gaps in multidisciplinary coordination and communication can hinder the seamless delivery of care.	Underrepresentation of certain groups and the exclusion of some RDs, making the taxonomy potentially incomplete and not universally applicable. As the study was conducted in the United Kingdom, findings may not generalise to other healthcare systems. The sample primarily included medical professionals, potentially overlooking social or mental health perspectives.
Weaver et al.	To explore barriers to timely diagnosis and referral for treatment of sarcoma.	Social constructionist framework for semi-structured interviews with 22 patients, 17 caregivers, and 21 healthcare professionals, recruited through a tertiary hospital and the Abby Basson Sarcoma Foundation (Sock it to Sarcoma!). Data were collected face-to-face, by phone, or electronically. Thematic analysis was applied to the interview data using NVivo software, following Braun and Clarke’s six phases. Data saturation was achieved when no new themes emerged. Coding and theme verification performed by two researchers.	Twenty-one healthcare professionals, 22 sarcoma patients, and 17 caregivers. Patients had a mean diagnosis age of 43 years and a median of 32 months since diagnosis, with most being female (*n* = 13), living in metropolitan areas (*n* = 16), and having high socioeconomic status (*n* = 13). Treatments included resection (*n* = 13), chemotherapy (*n* = 10), and radiation (*n* = 10). Caregivers, predominantly mothers (*n* = 9), had a mean age of 51 years, caregiving for an average of 33 months, with most from high socioeconomic backgrounds (*n* = 10). Healthcare professionals (11 females and ten males), had a mean age of 44 years and an average of nine years of experience, including orthopaedic surgeons (*n* = 3) specialising in sarcoma treatment and youth workers (*n* = 2) focusing on post-treatment care.	The study highlighted several healthcare access and delivery challenges in sarcoma diagnosis. Patients often delayed seeking help due to misperceptions about symptoms, attributing them to lifestyle factors or underestimating their severity. Diagnostic delays were also exacerbated by a lack of experience among healthcare professionals, particularly general practitioners and specialists unfamiliar with sarcoma, leading to misdiagnoses and extended referral times. Limited availability of healthcare services, especially in rural areas, further complicated timely consultations, as patients struggled to access primary care and experienced poor continuity of care. These factors contributed to prolonged diagnostic intervals and, in some cases, incorrect treatments that worsened outcomes.	Healthcare professionals assessed, diagnosed, and referred patients for further testing. In the context of sarcoma, they played a critical role in the appraisal and help-seeking intervals. Initially, patients might visit primary healthcare professionals who assess their symptoms, but delays often occurred due to misdiagnosis, with some healthcare professionals attributing symptoms to benign causes or lifestyle factors. In cases where sarcoma was suspected, referrals to specialist centres were needed, but miscommunication or lack of expertise in sarcoma could lead to additional delays.	Inclusion of only one general practitioner participant limits insights into general practitioner perspectives on diagnosing sarcoma. Patient recall bias may also affect the accuracy of the data. Reliance on self-nominated participants from the Sock it to Sarcoma Foundation may have reduced the diversity of experiences, leading to a higher socioeconomic status among participants. Some patients were recruited soon after diagnosis, and the study included a mix of sarcoma types, histologies, and demographics.
Willems et al.	To evaluate caregivers’ perspectives on providing and coordinating care for children with SMA.	This study used semi-structured interviews with 21 caregivers of children with SMA types 1 and 2, from three neuromuscular centres in Germany. Maximum variation sampling was used, based on characteristics such as patient age, illness stage, and family situation. Interviews were conducted in-person or via telephone by a psychologist between August 2019 and April 2020. Audio-recordings were transcribed and analysed using qualitative content analysis. Coding used a deductive-inductive approach supported by MAXQDA Plus 2020. Descriptive statistics were also computed using IBM SPSS Statistics.	Twenty-one caregivers of children with SMA Type 1 (62.0%) and SMA Type 2 (38.0%), from three neuromuscular centres in Germany. 71.4% of caregivers were female. Fifty-seven percent were aged between 20 and 40 years. Education: 28.6% had a university degree, 28.6% had alternative education. 76.1% of caregivers were married. 38.1% worked part-time, and 33.3% were unemployed. Children were between two to 15 years, with most being four (33.3%), three (19.0%), two (14.3%), and 11 (14.3%) years. Diagnosis of SMA occurred between five and 12 months for 47.6% of children, and one-third began drug therapy between one and three years old. 47.6% of children required ventilatory support, 33.3% needed enteral nutrition, and 71.4% relied on cough assist. 23.8% of children had no motor function milestones.	Caregivers often felt a mix of self-efficacy (confidence due to support networks and experience), and uncertainty (powerlessness). They experienced responsibility for care coordination, with some suggesting they could not rely on/ access healthcare professionals. Treatment delays and poor care coordination negatively impacted well-being. A shortage of local providers, difficult to navigate healthcare organisations, long wait times and insurance challenges were additional barriers to care. There was varying expertise amongst caregivers and healthcare professionals sometimes lacked in-depth knowledge of SMA.	Paediatricians, physiotherapists, occupational therapists.	Lack of clear theoretical framework for care coordination, a small sample due to the rarity of SMA, and exclusion of caregivers with limited German proficiency. The sample may have been biased, as it included only families from specialised academic neuromuscular centres. Focusing on caregivers of children with SMA types 1 and 2 may overestimate care coordination needs.
Withers et al.	To understand the psychosocial challenges and benefits of a diagnosis of Rubinstein-Taybi syndrome (RTS) experienced by parents.	Forty families registered with the RTS Australia support group were invited to participate. 13 parents participated in semi-structured telephone interviews, using open-ended questions, lasting 15–90 minutes. Audio-recorded interviews were transcribed, de-identified, and analysed following Miles and Huberman’s framework. Two researchers independently coded transcripts. Themes were identified iteratively using deductive and inductive coding, alongside a codebook.	Thirteen parents (ten mothers and three fathers) of 11 children (six males and five females) registered with RTS Australia support group. The average age of diagnosis was eight months (from birth to three years).	Parents experienced uncertainty before diagnosis, trying to understand why their child was different. Diagnosis provided validation, empowered communication, and access to services (allied health, education, and financial aid). There was ongoing uncertainty about long term prognosis. There was a perceived need for tailored counselling for families.	Participants reported involvement from paediatricians and allied health professionals; no other healthcare professionals were specified.	Low recruitment, self-selection bias, and the potential bias from recruiting participants from a support group. Poorly generalisable. Lack of data on the birth order of the affected child may have limited the interpretation of responses, and the retrospective study method introduces further limitations.
Witt et al.	To identify pathways to psychosocial care and the daily experiences and needs of patients and parents navigating the healthcare system, to inform tailored implementation strategies for families.	Semi-structured telephone interviews with 15 children with RDs and 74 parents of children with RDs. Interviews were conducted between June 2019 and February 2021, using an interview guide, and focused on healthcare journeys, living with an RD and access to psychosocial care. Participants were recruited via cooperating centres and online calls. Interview transcripts were analysed using deductive and inductive coding, facilitated by MAXQDA. 20% of interviews were assessed for intercoder reliability to improve rigour.	Seventy-four parents: 63 mothers and 11 fathers. Age range was 23 to 58 years old (mean = 37.5). 81% were in a relationship. Most parental interviews were for children aged 0–4 years (47%), but the target of ten interviews for the 13–17 age group was not met (*n* = 7). 15 children: Age range from 8 to 21 years (mean = 13.6). Nine boys and six girls. PLWRDs had a variety of RDs, with congenital abnormalities, malignancies, and metabolic disorders being more commonly reported in this study.	The study revealed five main themes. Living with an RD and caring for someone with an RD negatively affected mental wellbeing, everyday activities and social interactions. Diagnostic processes could be stressful and lengthy. Professional knowledge gaps could hinder care. There was a lack of awareness of psychosocial support services, which were very helpful for those able to access them. Families identified challenges with socio-legal issues, time constraints, and administrative issues hindered access to support services. They proposed that education and support with socio-legal issues would improve access to support services/ care.	Physicians for diagnosis, medical procedures, and ongoing therapy. Lack of interdisciplinary support. Families wanted better access to psychosocial services.	Uneven age group representation and under-recruitment of children and adolescents, and heterogeneity of rare diseases. Mothers were overrepresented, which may limit the generalisability of findings.
Xiao et al.	To investigate the experiences of PLWRDs and their families in accessing and receiving disease-modifying therapies for SMA.	Caregiver recruitment occurred via an SMA clinic in Ontario, Canada, between January and June 2022. Semi-structured interviews were conducted virtually, recorded and transcribed, then uploaded to NVivo to assist analysis. An inductive content analysis approach was used, consisting of four stages. Codes were developed and refined through team consensus. Participant feedback, peer debriefing and prolonged engagement were used to improve rigour.	Fifteen parents of children with SMA from an SMA clinic in Ontario, Canada. PLWRDs were: SMA Type 1 (*n* = 5), Type 2 (*n* = 5), and Type 3 (*n* = 5). 67% were male, 33% were aged 7–10 years. Treatment: 93% used nusinersen, 27% used onasemnogene abeparvovec, 13% used risdiplam. Caregiver characteristics: 73% female/ mother of the child. 53% aged 30–39 years. 73% had a university or college degree. 33% were unemployed, 27% were employed full-time, and 17% were full-time caregivers.	Two main themes: “Inequalities in access to disease-modifying therapies” (including varying regulatory approvals, prohibitively expensive treatments, and inadequate infrastructure). Caregivers navigated inequalities by relocating internationally to access treatments, and enrolling in clinical trials, lotteries grants, fundraising, and regional reimbursement programmes. Despite this, many still faced challenges with eligibility, therapy delivery, systemic delays preventing timely intervention. The second theme related to the experiences of families with disease modifying therapies, which related to hope for improved outcomes, fear of ineffective long-term treatment, urgent decision-making based on limited information and uncertainty of access/ future treatment availability.	Multidisciplinary team providing comprehensive care. Roles not specified.	Small sample size and not focused on decision-making regarding the initiation of disease-modifying therapies.

#### Service fragmentation

General practitioners or other non-specialist providers were often positioned as facilitating (or not) initial diagnoses (selected examples [47, 48, 58, 98, 108, 111]) with authors commenting on the lack of information offered [46, 54, 77, 82], involvement of multiple clinicians as part of obtaining a diagnosis/ seeking input [60, 69, 70, 88, 97, 108], misdiagnosis/ symptom dismissal, disputed diagnosis, and healthcare provider disbelief [37, 39–41, 49, 50, 52, 55, 57, 59, 60, 65, 69, 89, 95, 97, 99, 106, 107, 109, 111]. Healthcare access and delivery journeys commonly involved disconnected islands of knowledge [57, 106], which might be expected as the diseases were sufficiently rare for practitioners to have not previously encountered the disease [51]. Clinician knowledge gaps were particularly evident in RDs where symptoms were non-specific, such as adult growth hormone deficiency [108], or systemic sclerosis with interstitial lung disease, where PLWRDs found healthcare professionals failed to engage in disease progression, prognosis, or long-term management discussions [42]. Similar issues arose in male breast cancer, where men reported stigma and delays due to gender biases and limited awareness from their primary care physicians and oncologists [98, 99], or in lymphoma in pregnancy, where symptoms were dismissed due to pregnancy [96]. Fragmented care may be perceived as provider bias, racism, [100] or lack of appropriate education [65, 91]. Further, diagnostic trajectory delays and care fragmentation followed from challenges linking symptoms together and inconsistent disease terminology [111], having symptoms incorrectly attributed to anxiety [52], lifestyle or benign causes [65, 91], or symptoms overlapping with more common conditions or non-specific symptom management [49, 52, 111].

Studies [45, 54, 58] emphasised the frustration over communication challenges with healthcare providers, due to the technicality of information provided or lack of information, with long-term requirements or disease progression not always discussed or known [42, 53, 73, 92, 93]. Medical professionals were at times seen as being blasé over the impact of specific treatments and long-term outcomes [110]. These studies emphasised navigation-related struggles and the need for consistent personal or organisational advocacy [50, 51, 56, 60, 61, 68, 79, 82, 88, 96, 106, 108, 111]. Due to poor health system integration or communication gaps, families, including those living with the RD, reported adopting the role of intermediaries, care coordinators, advocates, and experts [51, 56, 59, 61, 62, 64, 68, 69, 78–80, 88–92, 94]. Where coordinated care models, such as joint clinics, disease-specific hubs, or COEs were in place, participants reported better outcomes, reinforcing the need for system-level strategies to support multidisciplinary collaboration and information sharing [59, 60, 63, 70]. In other studies, fragmented healthcare systems required those with RDs and those caring for them to navigate multiple unconnected providers [60, 72, 73, 114], including during pandemics [101] or economic sanctions [102].

Depending on condition and care setting, the reviewed studies included perspectives on the roles of a range of healthcare professionals, including paediatricians, geneticists, neurologists, clinical immunologists, physiotherapists, occupational therapists, and psychologists. While multidisciplinary care models were identified as important for managing complex conditions [69, 70, 105, 112], service provision was compromised by a lack of access to specialist or knowledgeable multidisciplinary care, or to care outside the hospital [37, 43, 44, 48, 55, 64, 68, 74, 77, 80, 87, 91, 106, 113, 114]. When considering the transition to adult services for children with RDs, structural and organisational coordination/ integration barriers impaired access to relevant and timely services [56, 60, 107], or was met with fear of losing relationships with important clinicians [107]. Compounding this, primarily in the United States, insurance restrictions resulted in significant concerns about compromised care coordination and access [76, 79, 85, 91, 104, 105, 108, 112]. Notably, despite the wide range of healthcare professionals identified, there was only limited commentary on the roles of nurses and particularly pharmacists in supporting PLWRDs. Hoffmann-Vold et al. [46] and Respondek et al. [58] suggested that lack of/ underutilisation of specialist nursing and paramedical care impaired healthcare pathways/ health education, while Denton et al. [42] identified nurses as having more time, and being more approachable, but having insufficient knowledge for full support.

Where participants reported positive experiences, healthcare professionals were described as empathetic, communicating clearly, and operating within a coordinated care environment. Parents, describing their experiences managing thalassaemia, highlighted how doctors and nurses at public and non-governmental organisations went beyond their duties in efforts to support families, increasing disease and treatment awareness, leading families to feel well-informed [84]. Cardão et al. [69] provided commentary on both positive and negative experiences, highlighting that positive experiences with specific healthcare teams were described as patient-focused and arising from within well-informed teams who could explain conditions. Mills et al. [96] suggested positive experiences are contingent upon clear communication, empathy, collaboration, and the addressing of wider needs beyond the treatment of, in this case, lymphoma. In turn, research participants commonly cited ‘luck’ (either in having knowledgeable clinicians or in personal circumstances) or ‘privilege’ (generally finances) as means of achieving better outcomes [41, 54–57, 63, 67, 88, 91, 95]. Overall, these findings align with Levedahl et al. [94], who suggested that confidence in healthcare delivery can be built by understanding what PLWRDs want from their care.

#### Financial and societal marginalisation

Individuals living with RDs incur substantial health service costs. Direct medical expenses included costs for (private) specialist consultations, genetic testing, assistive devices, and treatment. Insurance or national healthcare systems did not always fully cover these costs, resulting in significant financial barriers to accessing essential care [48, 67, 101, 105, 106, 112]. Financial exclusion limited PLWRDs’ access to medicine, health and disability services, and in the case of insurance access delays, worsened health and social/ educational outcomes [104]. In Iran, noted economic sanctions meant PLWRDs chose to postpone treatment, borrow money from others, or sell belongings/ property to meet costs while also acknowledging the poor quality of medicines available [102]. In some cases, families incurred out-of-pocket costs and delays when genetic samples had to be sent to overseas laboratories [67] or when they travelled to access diagnostic or specialist services unavailable locally [69, 70]. In the United States, insurance delays and denials impeded access to medication, equipment, and services [43, 85, 104], while step-therapy requirements forced some patients to try alternative medications that were ineffective or poorly tolerated [104]. Budgets available to state, insurers, and private individuals were finite, resulting in potential discontinuities in transitioning from paediatric to adult care, where some of the services provided were reduced [56]. Families and PLWRDs pursuing treatments unavailable in their home country relocated internationally. They might have also engaged in various fundraising efforts [93].

Broader societal RD costs included lost productivity, increased healthcare expenditures, and the economic impact of chronic illness and potential disability. Parents of children with RDs/ PLWRDs reported employment disruptions and a lack of job stability, with some forced to leave the workforce to provide full-time care or engage in treatment [37, 44, 62, 69, 79, 82, 104, 106, 107]. Several studies involving predominantly maternal caregiver samples described substantial caregiving responsibilities, including coordinating complex care, educating healthcare professionals, navigating funding and treatment access, and reducing participation in paid employment [69, 70, 77-79, 89, 93], with some identifying a gendered care-related burden [77] that left women feeling unsupported and undervalued by health systems. Children’s social lives, an important part of their development, were adversely impacted [80]. Self-imposed societal isolation was also widely reported, in some cases, shame was felt through the hereditary nature of diseases [53, 70, 75, 77, 82, 89, 94, 104, 109].

PLWRDs and their families reported that many of their health service experiences focused on immediate medical care, but providers did not consider follow-up or offer wider daily life and social support (e.g., mental health, social functioning, disability, and financial burdens) [37, 40, 56, 58, 66, 73, 75, 90, 109, 114]. PLWRDs and families turned to patient-led organisations and social networks for guidance on condition management. Shared lived experiences (identified through the creation of research poems) helped families of those with rare dementias to feel less isolated [39]. These groups often provided emotional support, practical advice, and up-to-date medical information that was otherwise difficult to access. However, some individuals struggled to find peers with similar experiences, while others identified that while living with or supporting someone with an RD, familial support changed or was not always available [107], or described abuse at the hands of family, friends, or society [82, 109].

#### Emotional isolation

Physical health needs related to RDs were reportedly prioritised over mental health or emotional needs [56, 57, 66, 71, 73, 94]. Emotional and informational needs for PLWRDs and their families varied depending on diagnostic clarity and treatment availability. However, studies often reported on the extreme stress associated with living with an RD (either through the treatment/ diagnosis process or as a direct result of the condition), including anxiety, depression, isolation, confusion, abandonment, and distress directly related to RD management or pathology [37, 39, 42, 44, 49, 83, 89, 94, 101, 104, 110, 111, 114]. In addition, in some cases, an RD diagnosis was reported to negatively impact peer and familial relationships [39, 44, 94, 107, 109, 110]. In the absence of family support, those living with an RD gained support from online communities [89, 97] or others in similar circumstances [81]. Unfortunately, online information was not always seen as reliable [53, 54], and while advocacy organisations were widely supported, engagement with these groups could also be emotionally demanding or distressing [54, 80].

Families described exhaustion, guilt, anxiety, and limited trust in health systems that were unprepared/ unwilling to provide adequate support (including where people felt clinicians lacked knowledge about social issues), often worsened by limited respite and social support [40–42, 57, 66, 71, 72, 82, 92]. For time-sensitive/ life-threatening conditions, such as primary haemophagocytic lymphohistiocytosis and cerebral adrenoleukodystrophy, families described being emotionally overwhelmed, with treatment marked by rapid deterioration, high-stakes decisions, and distressing delays [57, 86]. Families managing progressive, multi-system conditions, like spinal muscular atrophy, rare dementias, or rare neurodevelopmental disorders, emphasised sustained and evolving care, challenges in service continuity, and psychosocial strain associated with long-term needs [39, 63, 72, 91]. For conditions where genetic diagnoses could be obtained, such as primary immunodeficiency disease or Rubinstein-Taybi syndrome, families reported relief and anxiety, with diagnoses often providing gateways to services and bringing new uncertainties [67, 92]. However, contributing to distress was difficulty accessing/ receiving prognostic information [42, 73, 82].

### Impact

Geographically, studies originated from diverse healthcare contexts. Fifteen studies explored care in more than one country [39, 42, 46, 52, 57–60, 69, 79, 95–97, 101, 106]; three did not specify the countries where the research was conducted [52, 97, 101]. Most studies were conducted in countries with established RD policy instruments in place either before the study under review was conducted or at the time of the present review, namely Australia [38, 65, 69, 78, 79, 81, 83, 92, 95, 96, 106, 109], Canada [37, 39, 69, 70, 72, 79, 87, 93], Germany [42, 47, 48, 54, 58, 60, 66, 91, 98, 99], and the United Kingdom [39, 41, 42, 44, 45, 55, 57, 58, 62–64, 77, 86, 95, 103, 106, 110, 113, 114], though some studies were published before policy instruments were in place. Despite the preponderance of research from high-income countries, upper-middle income countries included Argentina [59], China [53], Iran [102], and Malaysia [40, 67], and lower-middle income countries Cameroon [82] and India [84]. Amongst these countries, Argentina, India, and Iran had RD strategies or legal frameworks in place. Many of the remaining studies were conducted in the United States [42, 43, 50, 51, 57–61, 68, 69, 71, 74, 76, 79, 85, 88, 95, 100, 101, 104–106, 108, 112]. Despite not having a national RDs strategy, the United States’ Orphan Drug Act incentivises development of RD drugs and, as a likely consequence, RD research.

Despite consistent messaging, direct policy citation was uncommon, and where identified, was often limited in depth or relevance (Table 4). At the time of review, eight studies were identified as having been referenced in literature identified through our Overton database search (Open Policy Ltd.) [43, 46, 56, 62, 63, 65, 70, 72]. Of these, five referred to the study and the impact of the disease on health service access and delivery [43, 56, 62, 63, 72]. Amongst these five studies, citations largely did not extend beyond the country in which the study was conducted; only two of the policy documents were prepared outside the home country/ region [43, 72]. The remainder of the identified policy documents cited a reviewed study and either noted the reviewed study’s exclusion from another review or that the study was published.

**Table 4:** Study country characteristics, including income level, health expenditure, rare disorder policies or strategy context, and evidence of policy impact/ citation of reviewed studies beyond academic literature

Authors	Year	Countries/ regions	Country income level at time study was published	Health expenditure as percentage of GDP (Total: Government schemes and compulsory contributory healthcare financing schemes: Voluntary healthcare payment schemes: Household out-of-pocket payments) at time study was published	Government rare disorder/ disease strategy or other policy instrument in place?	Citation identified via Overton search?
Akanuwe et al.	2020	United Kingdom	High	12:10:0:2	Yes, since 2013 (separate plans for parts of the United Kingdom published after this date)	No
Arora et al.	2023	United States	High	17:14:1:2	Yes, but not a national plan	No
Awada and Holcik	2024	Seven of ten Canadian provinces	High	11:8:2:2	Yes, since 2023 (national strategy for drugs)	No
Azahari et al.	2024	Malaysia	Upper-middle	Unknown. 2023 values are: 4:2:1:1	No	No
Bailey et al.	2023	United States	High	17:14:1:2	Yes, but not a national plan	No
Baptist et al.	2024	United States	High	Unknown. 2023 values are 17:14:1:2	Yes, but not a national plan	No
Bogart et al.	2024	Global	Not applicable	Not applicable	Not applicable	No
Bush et al.	2022	United States	High	17:14:1:2	Yes, but not a national plan	No
Byun et al.	2022	Victoria, New South Wales, Queensland, Western Australia (Australia)	High	10:7:1:1	Yes, since 2020	No
Camic et al.	2024	England, Wales, Canada, Switzerland, Singapore	High	United Kingdom: 11:9:0:2 Canada: 11:8:2:2 Switzerland: Unknown. 2023 values are 12:8:1:3 Singapore: Unknown. 2023 values are 4:3:1:1	England: Yes, since 2013 (action plan 2022); Wales: Yes, since 2013 (action plan 2022); Canada: Yes, since 2023 (national strategy for drugs); Switzerland: Yes, since 2014; Singapore: Yes, but not a national plan	No
Cardão et al.	2021	United States, Portugal, France, Spain, Australia, United Arab Emirates, Canada, Ireland, Israel, Sweden, Switzerland	High	United States: 17:15:1:2 Portugal: 11:7:1:3 France: 12:10:1:1 Spain: 10:8:1:2 Australia: 11:8:1:2 United Arab Emirates: 5:4:0:1 Canada: 12:9:2:2 Ireland: 6:5:1:1 Israel: 8:6:1:1 Sweden: 11:10:0:1 Switzerland: 12:8:1:3	United States: Yes, but not a national plan; Portugal: Yes, since 2015; France: Yes, since 2004; Spain: Yes, since 2009; Australia: Yes, since 2020; United Arab Emirates: No; Canada: Yes, since 2023 (national strategy for drugs); Ireland: Yes, since 2014; Israel: No; Sweden: No, ‘transmitted’ to government in 2012, expected to come out in 2025; Switzerland: Yes, since 2014.	No
Castro et al.	2020	Montreal, Quebec (Canada), including three families who resided outside Canada	High	13:10:2:2	Yes, since 2023 (national strategy for drugs)	Noted as an excluded study in the Cochrane meta-ethnography on children and young people with non-cancer chronic pain.
Ch’ng et al.	2022	Malaysia	Upper-middle	4:2:0:1	No	No
Costa et al.	2024	United States	High	Unknown. 2023 values are 17:14:1:2	Yes, but not a national plan	No
Currie and Szabo	2020	Western Canada (Canada)	High	13:10:2:2	Yes, since 2023 (national strategy for drugs)	UNESCO journal with a paper on the narratives of mothers about the process of their children becoming ill. The focus of reference is on routines limited by social isolation and exclusion; the external perception of difference with the social taboo and stigma of the disease; the lack of understanding and comprehension of family and friends in caring for the child, and a demand for multiple support services in contrast to fragmented governments that do not offer therapeutic support.
Davies-Abbott et al.	2024	Wales (United Kingdom)	High	11:9:0:2	Yes, since 2013 (action plan 2022)	No
de Dios García‑Díaz et al.	2022	Spain	High	10:7:1:2	Yes, since 2009	No
Denton et al.	2021	Germany, Italy, Spain, United Kingdom, United States	High	Germany: 13:11:0:1 Italy: 9:7:0:2 Spain: 10:8:1:2 United Kingdom: 12:10:0:2 United States: 17:15:1:2	Germany: Yes, since 2013; Italy: Yes, since 2001 - implemented in 2013; Spain: Yes, since 2009; United Kingdom: Yes, since 2013 (separate plans published for the different regions after this date); United States: Yes, but not a national plan	No
Diaz et al.	2022	United States	High	17:14:1:2	Yes, but not a national plan	Identified as part of a review of NPD types A, B and A/B treatment and diagnosis from national treatment and diagnosis protocols, published by the Ministry of Health in Saint-Denis, France.
Fitzgerald et al.	2021	Ireland	High	6:5:1:1	Yes, since 2014	No
Gerstein et al.	2020	United States	High	19:16:1:2	Yes, but not a national plan	No
Gill et al.	2021	United Kingdom	High	12:10:0:2	Yes, since 2013 (separate plans for parts of the United Kingdom published after this date)	No
Gorman and Farsides	2022	United Kingdom	High	11:9:0:2	Yes, since 2013	No
Güeita-Rodriguez et al.	2020	Spain	High	11:8:1:2	Yes, since 2009	No
Halbach et al.	2020	Germany	High	12:11:0:1	Yes, since 2013	No
Halley et al.	2022	United States	High	17:14:1:2	Yes, but not a national plan	No
Hitchen et al.	2024	United Kingdom	High	11:9:0:2	Yes, since 2013 (separate plans for parts of the United Kingdom published after this date)	No
Hoffman et al.	2022	United States	High	17:14:1:2	Yes, but not a national plan	No
Hoffmann-Vold et al.	2021	Belgium, Denmark, Finland, Greece, Norway, Portugal, Sweden, the Netherlands	High	Belgium: 11:8:1:2 Denmark: 11:9:0:1 Finland: 10:8:0:2 Greece: 9:6:0:3 Norway: 10:8:0:1 Portugal: 11:7:1:3 Sweden: 11:10:0:1 the Netherlands: 11:9:1:1	Belgium: Yes, since 2013; Denmark: Yes, since 2014; Finland: Yes, since 2014; Greece: Yes, since 2008; Norway: Yes, first year of implementation not available; Portugal: Yes, since 2015; Sweden: No, ‘transmitted’ to government in 2012, expected to come out in 2025; Netherlands: Yes, since 2013	Listed in an annual report of publications by the University Hospital of São João.
Ihne-Schubert et al.	2024	Germany	High	12:11:0:1	Yes, since 2013	No
Inhestern et al.	2024	Germany	High	12:11:0:1	Yes, since 2013	No
Ivarsson et al.	2021	Sweden	High	11:10:0:1	No, ‘transmitted’ to government in 2012, expected to come out in 2025	No
Jain et al.	2021	United States	High	United States: 17:15:1:2	Yes, but not a national plan	No
Johansen et al.	2024	Australia	High	Unknown. 2023 values are 10:8:1:2	Yes, since 2020	No
Johansen et al.	2023	Denmark	High	10:8:0:1	Yes, since 2014	No
John Cherian et al.	2023	United States, Australia, Canada, Europe	Europe: depends on country; Others: High	United States: 17:14:1:2 Australia: 10:8:1:2 Canada: 11:8:2:2	United States: Yes, but not a national plan; Australia: Yes, since 2020; Canada: Yes, since 2023 (national strategy for drugs)	No
Johnston-Devin et al.	2021	Australia, England, Wales, Singapore, United States	High	Australia: 11:8:1:2 United Kingdom: 12:10:0:2 Singapore: 5:3:1:1 United States: 17:15:1:2	Australia: Yes, since 2020; England: Yes, since 2013 (action plan 2022); Wales: Yes, since 2013 (action plan 2022); Singapore: Yes, but not a national plan; United States: Yes, but not a national plan	No
Johnston-Devin et al.	2022	Australia, England, Wales, Singapore, United States	High	Australia: 10:7:1:1 United Kingdom: 11:9:0:2 Singapore: 4:3:1:1 United States: 17:14:1:2	Australia: Yes, since 2020; England: Yes, since 2013 (action plan 2022); Wales: Yes, since 2013 (action plan 2022); Singapore: Yes, but not a national plan; United States: Yes, but not a national plan	No
Kerr et al.	2023	United States	High	17:14:1:2	Yes, but not a national plan	No
Kiani et al.	2024	Tehran, Sistan and Baluchestan, Hamedan, Yazd (Iran)	Upper-middle	Unknown. 2023 values are 6:3:1:3	Yes, since 2021	No
Kleinendorst et al.	2020	The Netherlands	High	11:9:1:1	Yes, since 2013	No
Kline et al.	2023	Online global health community; participant location not reported.	Not applicable	Not applicable	Not applicable	No
Ladores et al.	2023	United States	High	17:14:1:2	Yes, but not a national plan	No
Levedahl et al.	2022	Sweden	High	11:9:0:1	No, ‘transmitted’ to government in 2012, expected to come out in 2025	No
Lewis et al.	2020	United Kingdom	High	12:10:0:2	Yes, since 2013 (separate plans for parts of the United Kingdom published after this date)	No
Li and Yacyshyn	2023	Online forum	Not applicable	Not applicable	Not applicable	No
Lin et al.	2020	China	Upper-middle	6:3:1:2	Yes, list of rare diseases released in 2018	No
Litzkendorf et al.	2020	Germany	High	12:11:0:1	Yes, since 2013	No
Livermore et al.	2020	London (England)	High	12:10:0:2	Yes, since 2013 (action plan 2022)	No
Loizidou et al.	2023	England and Wales (United Kingdom)	High	11:9:0:2	Yes, since 2013 (separate plans for parts of the United Kingdom published after this date)	No
Long et al.	2021	New South Wales, Victoria, Queensland, South Australia, Western Australia (Australia)	High	11:8:1:2	Yes, since 2020	The Centre for International Economics published a report on the preventable burden of mitochondrial disease; cited for content around self-advocacy commitment, ongoing negotiation for advice, services, joint decision-making, and non-specialist services; and for noted lack of mental health support available for those with mitochondrial disease.
Martinussen et al.	2022	Victoria (Australia)	High	10:7:1:1	Yes, since 2020	No
Maxfield et al.	2021	Australia	High	11:8:1:2	Yes, since 2020	No
McLaughlin et al.	2022	South-East and North-West England	High	11:9:0:2	Yes, since 2013 (action plan 2022)	No
Mills et al.	2023	Australia, New Zealand	High	Australia: 10:8:1:2 New Zealand: 10:8:1:1	Australia: Yes, since 2020; New Zealand: Yes, since 2024	No
Ngoumou and Djouda Feudjio	2024	Yaoundé and Douala (Cameroon)	Lower-middle	Unknown. 2023 values are 5:1:0:3	No	No
Nguyen et al.	2020	Germany	High	12:11:0:1	Yes, since 2013	No
Nguyen et al.	2024	Australia	High	Unknown. 2023 values are 10:8:1:2	Yes, since 2020	No
Nixon et al.	2021	United States, United Kingdom	High	United States: 17:15:1:2 United Kingdom: 12:10:0:2	United States: Yes, but not a national plan; United Kingdom: Yes, since 2013 (separate plans for areas published after this date)	No
Palanisamy et al.	2024	Tamil Nadu (India)	Lower-middle	Unknown. 2023 values are 3:1:1:1	Yes, since 2021	No
Pasquini et al.	2021	United States	High	17:15:1:2	Yes, but not a national plan	No
Piercy and Nutting	2024	United Kingdom	High	11:9:0:2	Yes, since 2013 (separate plans for parts of the United Kingdom published after this date)	No
Ragan et al.	2021	Western Canada (Canada)	High	12:9:2:2	Yes, since 2023 (national strategy for drugs)	No
Respondek et al.	2023	France, Germany, Italy, Japan, Spain, the United Kingdom, United States	High	France: 12:10:1:1 Germany: 12:10:0:1 Italy: 8:6:0:2 Japan: 11:9:0:1 Spain: 9:7:1:2 United Kingdom: 11:9:0:2 United States: 17:14:1:2	France: Yes, since 2004; Germany: Yes, since 2013; Italy: Yes, since 2001 – implemented in 2013; Japan: Yes, since 1972 national programme; Spain: Yes, since 2009; the United Kingdom: Yes, since 2013 (separate plans published for areas after this date); United States: Yes, but not a national plan	No
Rintell et al.	2021	California (United States), Buenos Aires (Argentina)	United States: High; Argentina: Upper-middle	United States: 17:15:1:2 Argentina: 11:6:2:3	United States: Yes, but not a national plan; Argentina: Yes, legal framework, since 2011	No
Rodríguez-Laguna et al.	2022	United States, Germany and France	High	United States: 17:14:1:2 Germany: 12:11:0:1 France: 12:10:1:1	United States: Yes, but not a national plan; Germany: Yes, since 2013; France: Yes, since 2004	No
Rosenfeld et al.	2023	United States	High	17:14:1:2	Yes, but not a national plan	No
Simpson et al.	2021	United Kingdom	High	12:10:0:2	Yes, since 2013 (separate plans for parts of the United Kingdom published after this date)	Referenced as part of a wider study in England’s Rare Disease Action Plan.
Sisk et al.	2022	United States	High	17:14:1:2	Yes, but not a national plan	No
Soto Jansson et al.	2024	Sweden	High	11:10:0:2	No, ‘transmitted’ to government in 2012, expected to come out in 2025	No
Stamou et al.	2022	United Kingdom	High	11:9:0:2	Yes, since 2013 (separate plans for parts of the United Kingdom published after this date)	Healthcare Improvement Scotland’s national clinical guideline for dementia assessment, diagnosis, care, and support gives reference to the need for commissioning of specialist services for people with young-onset dementia, and the importance of stability of these services for continuity.
Verger et al.	2021	Spain	High	10:8:1:2	Yes, since 2009	No
Walton et al.	2022	United Kingdom	High	11:9:0:2	Yes, since 2013 (separate plans for parts of the United Kingdom published after this date)	No
Weaver et al.	2020	Australia	High	11:8:1:2	Yes, since 2020	List of Sir Charles Gairdner Osborne Park Health Care Group staff publications for 2020 from the Government of Western Australia North Metropolitan Health Service.
Willems et al.	2023	Germany	High	12:10:0:1	Yes, since 2013	No
Withers et al.	2021	Australia	High	11:8:1:2	Yes, since 2020	No
Witt et al.	2023	Germany	High	12:10:0:1	Yes, since 2013	No
Xiao et al.	2023	Ontario (Canada)	High	11:8:2:2	Yes, since 2023 (national strategy for drugs)	No

Note: Values are rounded independently to whole percentages; component values may therefore not sum exactly to reported total

The reviewed studies collectively emphasised the need for change to support PLWRDs and their families and noted that change requires collaboration between government, health workforce, PLWRDs, and advocacy organisations. While challenges vary across RDs and contexts, recommendations converge around key areas for attention and sustained investment. These include a focus on coordinated and equitable health service access; professional and public RD education; and the need for psychological support, peer support, and advocacy.

#### Financial and health service access

Across countries where the reviewed studies were conducted, health expenditure as a proportion of gross domestic product (GDP) varied, though was generally high; ranging from approximately 3% in India to 19% of GDP in the United States; and characterised by higher government and compulsory contributory financing schemes and low, though variable, out-of-pocket payments. Upper- and lower-middle-income settings identified in the reviewed studies [40, 53, 67, 82, 84, 102] had lower overall health expenditure as a proportion of GDP and greater reliance on out-of-pocket payments relative to pooled financing, indicating reduced financial protection for the population. These cross-country comparisons should be interpreted descriptively, as neither the included studies nor our review were designed to support formal cross-country causal comparisons between income level, health system financing, and reported access barriers.

When considering financial and health service access for PLWRDs in the reviewed studies, increased funding and equitable treatment access, particularly for costly or novel therapies, emerged as important requirements [40, 53, 55, 58, 74]. Studies suggested that health systems should implement standardised or clearer RD care pathways or tools, ensuring physicians follow best practices for timely diagnosis, coordinated/ integrated care, and follow-up [37, 49, 74, 89, 91], although noting that long-awaited diagnoses may not always resolve uncertainties [81]. These pathways should involve national guidelines, (potentially controversial) RD registries [91, 96], and streamlined funding and insurance authorisation processes [85, 104, 107]. When considering diagnosis, studies supported expanding access to genetic testing for earlier and more accurate diagnoses [67, 89]. However, Martinussen et al. [81] provide a nuanced perspective, suggesting that the emotional value of genomic results must be balanced against clinical utility and the possibility of de-medicalising treatment.

Digital strategies such as telehealth, integrated standardised digital records, and multidisciplinary team approaches were proposed to streamline health service delivery and reduce caregiving burden [47, 52, 62, 64, 68, 69, 87, 88, 96, 101]. Others caution that digital health solutions may require nuanced approaches to adequately meet the needs of all PLWRDs, particularly those with complex care requirements, limited digital literacy, or requirements for in-person assessment [38, 63, 101].

#### Education

Authors consistently recommended targeted healthcare professional training to improve symptom recognition, reduce diagnostic delays, and promote person-centred, empathetic care [39, 44, 46–49, 55, 59, 68–70, 76, 77, 83, 94, 105, 108, 111, 112, 114], particularly for early diagnosis and multidisciplinary management [43, 60, 96, 111]. The reviewed studies were less consistent about which healthcare professionals should hold knowledge of RD management (often primary care and emergency medicine clinicians) or whether this education should be at an undergraduate or postgraduate level [95]; however, implicitly for many, the focus appears to be on diagnosis management [41, 44, 57, 59, 68, 69, 78, 107, 112]. Some studies emphasised the importance of integrating general practitioners into RD care through increased awareness and training [46, 49, 59, 114]. These studies argued that improved primary care knowledge is essential for earlier diagnosis and management, especially in settings with limited access to specialists. In contrast, others advocated for centralised specialist-led models, such as COEs or disease-specific teams, to ensure high-quality, accurate care [58, 80, 83, 96]. When considering curriculum development and formal training, studies highlighted the need for inclusive education and ongoing professional development to support early condition management and recognition [46, 48, 108, 111]. The integration of creative approaches to education, such as poetry in dementia training [39], also potentially reflected an evolving appreciation of empathetic and emotionally resonant learning.

Parallel to health workforce education, the reviewed studies recommended improved education for PLWRDs, their families, and the public [46, 58, 60, 83, 98, 99, 103, 111]. Part of this education pathway involves co-designed educational tools, information portals (including separately for long-term care needs), empowerment programmes, and care models to ensure interventions reflect RD lived experiences and empower RD populations [68, 72, 87, 88]. Others stressed the need for public awareness campaigns, especially for lesser-known conditions [58, 65, 67, 101], and tailored, up-to-date disease-specific information and support systems for those managing complex or misunderstood conditions or conditions that are additionally uncommon in subpopulations [99, 109, 113].

#### Psychological support, peer support, and advocacy

Given the lack of focus on psychosocial and mental health needs, studies recommended the integration of psychological and mental health support for PLWRDs and families into RD care models. Such integration varied from counselling services [43, 57, 66, 68, 72], respite [40, 57, 58, 70, 78], and palliative care [40], to mentoring/ mental health support tailored to specific life stages, diagnoses, and uncertainty [37, 38, 58, 86, 87, 89], and play therapy for paediatric PLWRDs [57]. While recommending services, barriers were identified, including the time commitment of attending being prohibitive [69], low uptake [112], potential skill gaps in delivering services to PLWRDs and their families [78], potential cultural biases towards receiving psychological support [40], or lack of service awareness [66]. Studies noted the value of culturally safe, family-centred emotional support (with or without an emphasis on informal social support networks) that recognised the unique experiences of additionally marginalised or underrepresented populations [68–70, 84, 87, 100]. These populations included Indigenous communities and rural residents, but also populations in which a particular RD is especially uncommon [66, 84, 100]. Notably, while studies encouraged co-design and shared decision-making with PLWRDs and families [50, 55, 56, 59, 60, 68, 72, 73, 75, 85, 103], Simpson et al. [62] emphasised that acting in an expert role brings with it anxiety.

Incorporating advocacy organisations into standard care practices and enhancing their role were suggested as ways to bridge informational gaps and empower PLWRDs to navigate health services and influence policy more effectively [59, 60, 82, 85, 104, 114]. In turn, authors recommended investment in peer/ community support networks to facilitate engagement, reduce isolation, and support individuals through their RD journeys [39, 43, 66, 73, 81, 82, 87, 89, 105, 106, 109].

## Discussion

While contexts, conditions, and health systems varied, findings from our scoping review of 78 publications highlight several persistent issues: limited specialised care availability, diagnostic delays, inconsistent communication and coordination across services, and disproportionate burdens placed on PLWRDs and families to navigate care systems and manage structural areas of service neglect. We further document consistent recommendations on the need for clear diagnosis, management, monitoring, and support pathways; health workforce and service-user education; and embedding psychological and peer support into care delivery. Despite this consistency, the reviewed studies, though conducted using a variety of data collection/ analysis methods, often did not apply inclusive research practices, and had limited identifiable policy citation. Publication was also concentrated within a small number of journals and publishing houses, while the studies covered only a narrow subset of rare disorders. Together, these findings indicate limitations in the breadth, inclusivity, and policy visibility of the published evidence base.

Lack of policy traction in addressing pervasive RD care access barriers may, in part, stem from how experiences of PLWRDs are framed, often as individualised care journeys, as seen in many of the reviewed papers. In response, given condition rarity, policymakers and system leaders may respond with sympathy but not enact structural change, and clinicians may see their lack of learning about a condition as unimportant. If this parallels our own research into other vulnerable populations [115], peer reviewers may even consider these narratives to be “atrocity stories”. While accounts in the RD community powerfully convey suffering and systemic failure, they also render structural issues anecdotal, rather than indicative of broader, repeated inequities. These stories become tragic outliers rather than drivers of reform. Consequently, without intentional translation mechanisms for RD research, studies, such as those we reviewed, risk becoming cyclic in documenting gaps rather than closing them. Embedding implementation science, co-governance, or targeted dissemination strategies into research could be one way to ensure findings move from publication to practice.

Alternatively, the rarity, complexity, treatment costs, and heterogeneity of RDs may contribute to their placement in the “too hard basket” (as seen in the current review through participants' diagnostic odysseys and fragmented access to health services), where the perceived scale of the issue does not justify targeted investment, despite profound costs [7, 8, 116], and ongoing impacts on those affected and financially dependent on a functioning health system. PLWRDs have protracted diagnostic odysseys [37, 49, 50, 59, 68, 76, 80, 81, 83, 97] and inconsistent (often expensive) access to specialist care. Notably from our review, much of the focus of health service delivery occurs at contact with specialist and medically-centred services that adopt a largely biomedical focus. This points to a significant gap in cohesive condition management, particularly given the lack of consideration of other healthcare professional roles and the need to upskill healthcare professionals who may be more involved in condition management rather than diagnosis and treatment changes. Framing of RDs as resource-intensive undermines health system commitments to fairness and reinforces structural discrimination against already marginalised populations. Genomic medicine, data integration, and personalised care model advancements show that meaningful solutions are possible when system priorities align with need.

Insufficiencies arising from RD healthcare delivery extend beyond clinical encounters to psychosocial impacts at individual, familial, and community levels. Although emotional strain, financial hardship, stigma, societal marginalisation, and fragmented social support were frequently documented, these consequences remained deprioritised within health systems, while the recognition and support needed to address them were conspicuous by their absence. Several studies in this review described the cost of time off work, loss of income, and the strain created by fragmented social support, highlighting that RDs affect the wider family [44, 59, 72, 104, 107, 109]. While studies did not directly examine how boundaries between health, education, and social sectors overlap in shaping support for families living with RDs (see exceptions [89, 90, 109]), the identification of clinical service gaps and unmet psychosocial needs suggests that these boundaries are consequential. Without recognising how these sectors intersect to shape everyday experiences, responses to RDs risk remaining confined to clinical care, overlooking systemic and relational dimensions required for coordinated, equitable support [21].

The 78 included articles were distributed across 47 journals; publication was notably concentrated in the Orphanet Journal of Rare Diseases, which accounted for 21 articles, and among a small number of major commercial publishers. While these journals provide valuable knowledge consolidation platforms, as suggested in our review, only a narrow subset of recognised rare disorders appear to be covered. As a result, health services experiences for individuals diagnosed with thousands of other disorders remain largely unexamined. Such knowledge gaps likely affect RDs that are ultra-rare, clinically complex, poorly resourced, or with poorly defined symptomatology. This narrow focus may have contributed to repeating patterns of recommendations in extant literature reviews [117, 118] around care coordination, psychosocial support, and equitable access without substantial policy or system impact. The persistence of these patterns highlights structural problems within the research and publishing ecosystem: evidence generation and dissemination are concentrated, exclusive, and poorly connected to implementation pathways for translating findings into cross-sectoral action.

Although the role of health system architecture, and specifically funding and economics, was not the primary focus of this review, it remains important in influencing outcomes for PLWRDs. How health systems are financed, whether through public provision, private insurance, or mixed models, determines who receives timely diagnosis, treatment, and coordinated care. In systems reliant on insurance, coverage gaps and exclusions may leave families facing substantial out-of-pocket costs for genetic testing, specialist consultations, and ongoing management [52, 56, 76, 82, 85, 104, 105, 108, 112]. Limited or inconsistent funding streams can entrench fragmentation and result in PLWRDs having to navigate multiple agencies that operate under separate eligibility and reimbursement frameworks [37, 74, 79, 88, 93]. Economic pressures may also determine which RDs attract research investment or service prioritisation, privileging those with stronger advocacy or commercial viability of treatment. These funding dynamics highlight the importance of developing sustainable, equity-oriented service models that reduce financial vulnerability and enable coordinated, integrated, cross-sectoral RD responses [119].

### Recommendations

Marginalising the narratives of PLWRDs, systemic avoidance, and health system structural impediments perpetuate inertia and underscore the value of better formal service-experience reporting and, although not reported on in this scoping review, data on costs avoided through optimal management of RDs. In addition to recommendations reported earlier in the results (see “Impact” section), Table 5 outlines health workforce, policy, research, and advocacy recommendations arising from our review. While individual countries have developed policies and strategies, this review supports moves towards creating a coordinated global action plan, as requested under WHA78.11, to align national efforts under a common framework with the aim of securing equitable access, data sharing, and sustainable funding. Given the disproportionate impact of RDs on children, initial focus on this population is pertinent and would advance relevant Sustainable Development Goals and support commitments established under WHA78.11, including development of the ten-year global action plan and coordinated, cross-sectoral action.

**Table 5:** Recommendations arising from the scoping review

Health workforce
• Invest in training that builds awareness and knowledge of all clinicians around early RD detection and management, particularly those working in primary healthcare and specialist services. • Support interdisciplinary collaboration between clinicians working in different parts of RD condition management. • Recognise and fund the time required for clinicians to engage in learning about and connecting with specialist networks (for clinician peer support and care delivery) and PLWRD/ RD community-led support.
Health policy
• Develop and implement a global action plan on RDs, as requested under WHA78.11, which includes, as part of the plan, strategies for care delivery that embed the skills of clinicians globally and maintain platforms that enable clinicians and policymakers to engage with current research. • Strengthen data systems and equitable access to diagnostic tools. • Co-design policies with RDs communities. • Invest in funding models that prioritise value-based care, including investment in workforce capability, interdisciplinary coordination, and culturally safe relationship-oriented care models.
Research
• Conduct research that reflects the interdependency of RDs in health, disability, education, and social systems in shaping the experiences of PLWRDs. Translate qualitative insights into service design principles, such as care coordination models, workforce enablers, and funding pathways. • From the outset, partner with PLWRDs, their families and other caregivers, and advocacy groups as co-researchers, not just participants. • Require publicly accessible outputs and publish accessible summaries of research for participants, advocacy groups, and policymakers alongside academic outputs. • Advocate for special issues on RDs in broader journals to normalise inclusion within the wider health research discourse.
Advocacy
• Challenge narratives of rarity that lead to policy and research neglect by reframing RDs as a collective public health issue requiring a coordinated national response. • Advocate for equity-focused research investment, including studies on diagnostic delay, treatment access, and long-term outcomes among underserved populations.

### Limitations

While extensive, the databases we searched were limited to those available through the primary author’s institution, and the restriction to English-language, peer-reviewed publications may have excluded relevant evidence. The search covered studies published from January 2020 to May 2024. Although further research will have emerged since the search was completed, the review remains relevant because it identifies recurring system-level barriers across rare disorders, populations, and health-system contexts. Its contribution lies in synthesising persistent patterns in health-service access and delivery, research inclusivity, and policy citation. These challenges continue to be recognised in recent international literature [5, 25].

In addition, the broad focus on RDs may have obscured condition-specific nuances and context-dependent insights; in turn, the previously acknowledged limitations in RD naming (see “Methods”) further perpetuate issues with identifying differences in individual RDs. Finally, while the review aimed to highlight the policy impact of included studies, policy takes time to change; one study suggests it takes, on average, seven to 19 years for research to impact society [120]. The identified impact of reviewed studies will hopefully grow with time and at present demonstrate impact only as it relates to being cited or mentioned in documentation; further work is required to understand how these studies informed recommendations, policy rationale, or any programme design. However, temporal considerations require cautious optimism given international changes in access to grant funding for equity-focused (predominantly qualitative social science) research [121–123]. Although the cited examples do not demonstrate a consistent international trend, we hypothesise that such funding gaps will disproportionately affect research in the RD, disability, and equity space. This concern is supported by findings from our previous scoping review, which showed the dearth of funding for qualitative disability research [13].

### Future research

Advancing the RD health services research field requires focused attention to key areas, including population diversity and intersectional experiences; variation across health system and service models; development of inclusive and developmentally appropriate qualitative methods; and understanding the role of policy in influencing qualitative research.

#### Population diversity and intersectional experiences

Despite consistent findings on health service access and delivery fragmentation, the reviewed studies showed only limited insight into population-specific characteristics and how intersecting characteristics shape experiences, highlighting the need for research on the compounding effects of being rare within the RD community. This gap is reinforced by the limited number of qualitative studies across many disorders, meaning the current evidence base covers only a narrow range of RDs despite over 10,000 identified RDs worldwide. Future research should first focus on intersecting populations at higher risk of poor health outcomes, such as disabled people, Indigenous populations, and those without a specific named disorder. This research base should, therefore, extend towards more explicit participation-focused, rights-based, social model, and lived experience-informed work. Conducting such research could support more nuanced understanding of how access challenges are differentially experienced, informing targeted health service and policy responses.

#### Variation across health system and service models

Furthermore, future research should prioritise understanding how different health system models shape PLWRDs’ experiences. The current dearth of research in low-income countries and in countries without established RD strategies limits insight into how health service access and delivery vary across contexts, particularly where healthcare infrastructure is less developed. Comparative research across jurisdictions is, therefore, needed to examine how variations in healthcare models influence access, care coordination, and outcomes (amongst other things), and to identify transferable lessons that may inform effective and equitable policy development.

#### Inclusive and developmentally appropriate qualitative methods

Few studies directly captured the experiences of children living with rare disorders or PLWRDs who have a learning disability. This absence is concerning, given that about half of all RDs are found in children [2]. This restricts insights into: (1) early-life trajectories where diagnosis, service delivery, and care coordination are most consequential for effective support and RD management [5], and (2) key system interfaces such as transitions between paediatric and adult services that are important for understanding service delivery barriers [56, 60, 107]. Further, for paediatric populations, evidence is often mediated through caregiver perspectives, raising questions about whose voices are represented and how these voices shape identified priorities.

Future research should prioritise inclusive and adaptive methods that support participation of a broader range of RD populations; a significant step in this process is building supported decision-making practices into research. Such practices begin with the adoption of Plain Language and other accessible materials (which some of our reviewed studies used) but go further by considering alternative communication tools, co-design and participatory approaches, flexible data collection methods, culturally and linguistically responsive designs, and, particularly in the case of RD research, sampling strategies facilitating engagement beyond advocacy groups and healthcare professional recruitment. These approaches are increasingly adopted outside health services research, including disability studies and education research [124–127], offering valuable models for RD studies.

#### Policy within qualitative research

Studies inconsistently linked health service access and delivery to specific interventions or policy responses. Where studies referenced health system policies or state interventions, they typically presented them as background context rather than examining how these policies shaped lived experiences for PLWRDs. Conversely, there was limited evidence that the reviewed research informed policy. Taken together, the studies suggest a cyclical disconnect in which research has not meaningfully informed policy, and policy has not systematically informed research, resulting in documentation of persistent challenges without corresponding system-level change. This reflects not simply a reporting gap, but a more fundamental limitation in how associations can be understood and studied, particularly when considering differences in RD reimbursement approaches, care coordination models, and other problems of service complexity within established multi-priority health systems. This disconnect can be seen as shifting responsibility for bridging system gaps onto individuals and families, with equity implications, as those with greater social, cultural, and material capital may be better positioned to navigate and compensate for fragmentation.

Although established health services and access to care conceptual frameworks [128, 129], including those in the RD space [129], provide useful starting points, they do not fully capture the depth and breadth of this conceptual gap, nor the complexity, fragmentation, and condition-specific variability characteristic of RDs as an economically impactful component within a larger health system. It can be argued thus that the field lacks conceptual clarity necessary for a complete mapping of these associations, and further work is needed to develop frameworks grounded in RD care realities and health system functioning.

## Conclusion

This scoping review underscores an urgent need to hero the voices of PLWRDs; an especially pressing need given the narrow scope of conditions typically represented in qualitative research. Our review suggests that study design choices, including a lack of participatory approaches, constrain the meaningful involvement of PLWRDs in research. Such a constraint limits the extent to which people’s experiences inform RD support, treatment, and policy development. Despite consistent messages around poor health service access and delivery, and a host of structural and systemic biases that negatively impact the individuals’ emotional, financial, and societal engagement, the perspectives of PLWRDs are often marginalised, within study design and had limited identifiable citation in Overton-indexed documents. RD care must be redesigned to be equitable, coordinated, and responsive to the realities of PLWRDs. One part of this, given overwhelmingly comparable accounts, is to consider an RDs global action plan. Such a plan should address the need for coordinated care that is responsive to the lived realities of PLWRDs, including support beyond biomedical health service delivery. The reviewed studies consistently emphasised the value of supported decision-making, peer support, and holistic care to address emotional and social burdens. RD systems must move beyond fragmented, specialist-driven models toward inclusive, relationship-based approaches grounded in the priorities of the communities they serve.

## Data Availability

Not applicable.

## Abbreviations

AGHD: Adult growth hormone deficiency
AHP: Acute hepatic porphyria
ARD-ILD: Autoimmune rheumatologic disease–associated interstitial lung disease
ATTR: Transthyretin amyloidosis
ATTR-CM: Transthyretin amyloid cardiomyopathy
ATTR-PN: Transthyretin amyloid polyneuropathy
BD: Behçet disease
CDG: Congenital disorders of glycosylation
CEL: Congenital ectopia lentis
CF: Cystic fibrosis
CFM: Craniofacial microsomia
CLOVES syndrome: Congenital lipomatous overgrowth, vascular malformations, epidermal nevi, scoliosis/skeletal and spinal anomalies
COE(s): Centre(s) of excellence
CRPS: Complex regional pain syndrome
DCC(s): Disorder(s) of the corpus callosum
DOI: Digital object identifier
DS: Dravet syndrome
EDS: Ehlers–Danlos syndrome
GDP: Gross domestic product
HAE: Hereditary angioedema
HSCT: Haematopoietic stem cell transplant
HSD: Hypermobility spectrum disorders
JDM: Juvenile dermatomyositis
K-T: Klippel–Trénaunay syndrome
LSD(s): Lysosomal storage disease(s)
MBC: Male breast cancer
M-CM: Megalencephaly-capillary malformation
MDS: MECP2 duplication syndrome
MRCD: Mitochondrial respiratory chain disorder
NDD(s): Neurodevelopmental disorder(s)
NPD: Niemann–Pick disease
OI: Osteogenesis imperfecta
pHLH: Primary haemophagocytic lymphohistiocytosis
PID: Primary immunodeficiency disease
PLWRD(s): Person/ people living with a rare disorder(s)
PPA: Primary progressive aphasia
PROS: PIK3CA-related overgrowth spectrum
PSP: Progressive supranuclear palsy
RD(s): Rare disorder(s)
RND: Rare neurological disorders
RTS: Rubinstein-Taybi syndrome
SM: Systemic mastocytosis
SMA: Spinal muscular atrophy
SSc-ILD: Systemic sclerosis–associated interstitial lung disease
UCD(s): Urea cycle disorder(s)
UDP-Vic: Victorian Undiagnosed Disease Program
WD: Wilson disease
YOD: Young-onset dementia

## Footnotes

1 In Aotearoa New Zealand and the United Kingdom, the term learning disability is increasingly preferred by self-advocacy organisations, including People First. Internationally, and within much of the health services (research) literature, the term intellectual disability remains dominant. For the purposes of this scoping review, the currently preferred term in Aotearoa New Zealand, where most of the research team is based, has been applied.
